# Phylogenetic Interrelationships of Ginglymodian Fishes (Actinopterygii: Neopterygii)

**DOI:** 10.1371/journal.pone.0039370

**Published:** 2012-07-11

**Authors:** Adriana López-Arbarello

**Affiliations:** Bayerische Staatssammlung für Paläontologie und Geologie, München, Germany; Biodiversity Insitute of Ontario - University of Guelph, Canada

## Abstract

The Ginglymodi is one of the most common, though poorly understood groups of neopterygians, which includes gars, macrosemiiforms, and “semionotiforms.” In particular, the phylogenetic relationships between the widely distributed “semionotiforms,” and between them and other ginglymodians have been enigmatic. Here, the phylogenetic relationships between eight of the 11 “semionotiform” genera, five genera of living and fossil gars and three macrosemiid genera, are analysed through cladistic analysis, based on 90 morphological characters and 37 taxa, including 7 out-group taxa. The results of the analysis show that the Ginglymodi includes two main lineages: Lepisosteiformes and †Semionotiformes. The genera †*Pliodetes*, †*Araripelepidotes*, †*Lepidotes*, †*Scheenstia,* and †*Isanichthys* are lepisosteiforms, and not semionotiforms, as previously thought, and these taxa extend the stratigraphic range of the lineage leading to gars back up to the Early Jurassic. A monophyletic †*Lepidotes* is restricted to the Early Jurassic species, whereas the strongly tritoral species previously referred to †*Lepidotes* are referred to †*Scheenstia*. Other species previously referred to †*Lepidotes* represent other genera or new taxa. The macrosemiids are well nested within semionotiforms, together with †Semionotidae, here restricted to †*Semionotus*, and a new family including †*Callipurbeckia* n. gen. *minor* (previously referred to †*Lepidotes*), †*Macrosemimimus*, †*Tlayuamichin*, †*Paralepidotus*, and †*Semiolepis*. Due to the numerous taxonomic changes needed according to the phylogenetic analysis, this article also includes formal taxonomic definitions and diagnoses for all generic and higher taxa, which are new or modified. The study of Mesozoic ginglymodians led to confirm Patterson’s observation that these fishes show morphological affinities with both halecomorphs and teleosts. Therefore, the compilation of large data sets including the Mesozoic ginglymodians and the re-evaluation of several hypotheses of homology are essential to test the hypotheses of the Halecostomi vs. the Holostei, which is one of the major topics in the evolution of Mesozoic vertebrates and the origin of modern fish faunas.

## Introduction

**Figure 1:**
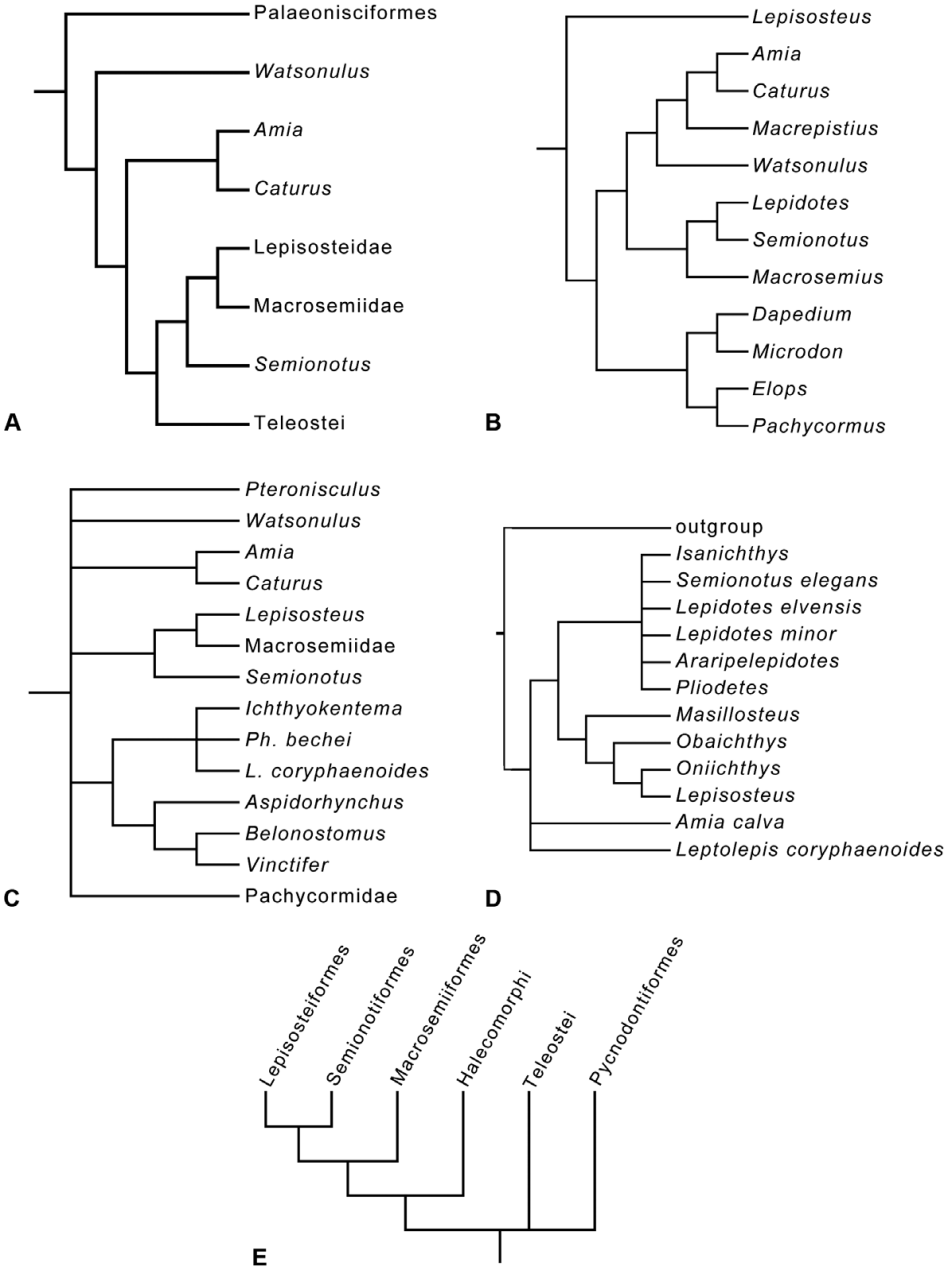
Previous hypothesis of “semionotiform” phylogenetic relationships. **A**, Olsen & McCune, 1991 [8]; **B**, Gardiner et al. 1996; **C**, Brito, 1997 [9]; **D**, Cavin & Suteethorn, 2006 [10]; **E**, Grande, 2010 [13].

**Figure 2:**
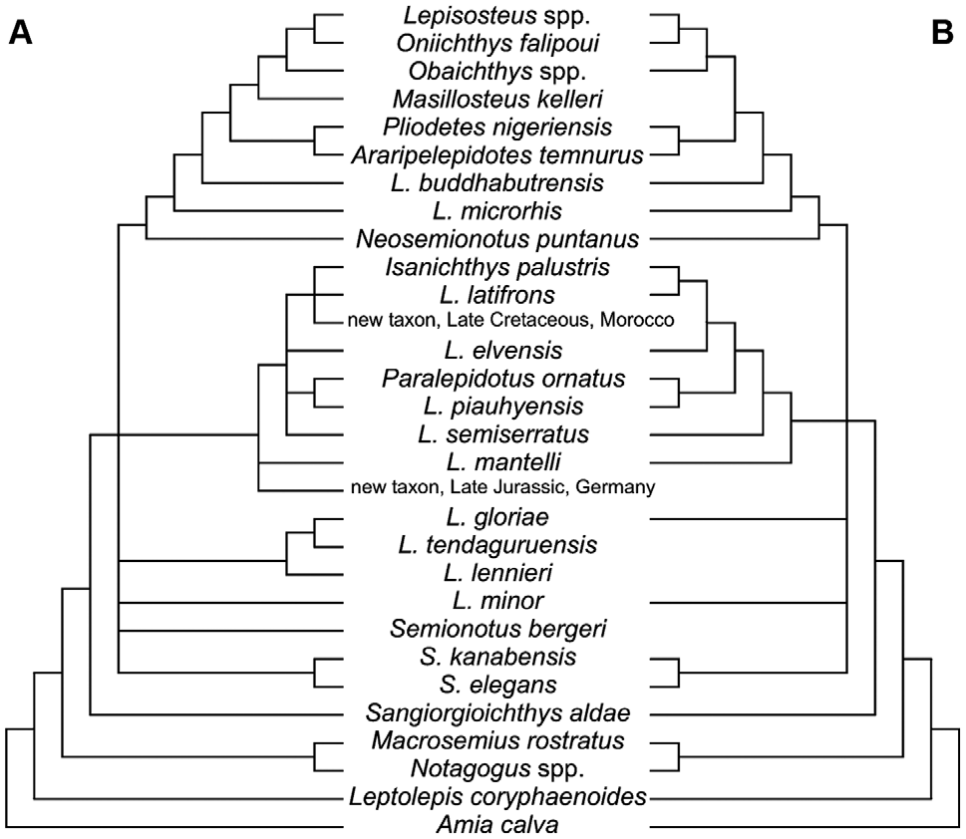
Hypotheses of “semionotiform” phylogenetic relationships of Cavin [**12**]. **A**, Strict consensus tree of 71 most parsimonious trees after the first cladistic analysis based on 31 taxa and 42 informative characters. **B**, strict consensus tree of 26 most parsimonious trees after the third analysis based on 25 taxa (excluding taxa with 35% or more missing data) and 45 informative characters (three multistate characters were split).

A very important step in the evolution of the actinopterygian fishes is the origin of the Neopterygii, with the acquisition of a better control of the movements of both dorsal and anal fins, resulting in an improvement in their swimming capabilities. They additionally acquired several modifications in the skull, which allowed the evolution of different feeding mechanisms and consequently the colonization of new ecological niches. All of these characters represented major improvements, so that the Neopterygii became the dominant group of fishes (and, thus, taxonomically of vertebrates in general), and they also include the vast majority of the modern fishes, the teleosts. Among basal neopterygians, the family †Semionotidae has played a critical role when trying to understand the origin and relationships of the other neopterygian lineages. Regan [1] considered †Semionotidae to represent the ancestral stock from which all other neopterygian lineages, including teleosts, had evolved. Brough ([2]: p. 108) proposed that most, if not all holosteans arose from the families †Semionotidae and †Eugnathidae independently. Danil’chenko [3] and McAllister [4] classified the †Semionotidae within an order Amiida or Amiiformes distinct from the Lepisosteiformes, but Gardiner placed them together with the Lepisosteidae in a superfamily Semionotoidea [5] or order Semionotiformes [6]. Patterson [7], after including the dapediids in †Semionotidae, concluded that semionotids represent a grade-group (para- or polyphyletic) and placed them as basal halecostomes of uncertain relationships. Recent phylogenetic analyses have demonstrated the monophyly of a major clade including †Semionotidae, Lepisosteidae, and †Macrosemiidae (Figs. 1, 2) [8]–[13].

Originally based on the Upper Triassic genus †*Semionotus* from central Europe, the family †Semionotidae has become a “wastebasket” taxon for many taxa of basal neopterygians that cannot be confidently assigned to any of the other groups, spanning from the latest Permian to the late Cretaceous. However, although it turned into one of the most diverse taxon of fossil neopterygians, semionotid monophyly, the interrelationships of the taxa included and even their alpha taxonomy has not been satisfactorily established so far. The family †Semionotidae was created by Woodward [14] to include †*Semionotus*, †*Dapedium*, †*Tetragonolepis*, and the perleidiforms †*Pristisomus* and †*Cleithrolepis*. †*Lepidotes*, which was previously considered to represent its own family †Lepidotidae Owen, 1860 [15], was later added to †Semionotidae by Woodward [16], together with a variety of genera, including some other perleidiforms like †*Colobodus*. With the time, †Semionotidae became even larger, containing about 20 genera of diverse basal neopterygians, and both the concept of the family as well as its phylogenetic relationships became more and more confused. Lehman [17] and Wenz [18] first revised the family and separated the deep-bodied genera †*Dapedium*, †*Tetragonolepis*, †*Heterostrophus* and †*Dandya* in the family †Dapediidae. Olsen & McCune [8] restricted the †Semionotidae to †*Semionotus* and †*Lepidotes* and diagnosed the family by the presence of dorsal ridge scales and a large, posteriorly directed process on the epiotic. However, the distribution of the characters given by Olsen & McCune [8] within and outside the family is not clear [19]–[23]. The most recent taxonomic hypothesis for †Semionotidae is that of Wenz [21], who proposed a new arrangement of the taxa included according to the number and disposition of suborbital bones, though she did not provide a new formal diagnosis for the family. Therefore, the family †Semionotidae has still neither been satisfactorily defined, nor diagnosed.

Eleven genera are currently ascribed to the family †Semionotidae (in alphabetic order): †*Araripelepidotes* Santos, 1990 (Early Cretaceous of Brazil) [24], †*Lepidotes* Agassiz, 1832 (Early Jurassic of Europe, though numerous species have been referred to this genus worldwide) [25], †*Neolepidotes* Chang & Chou, 1977 (Early Cretaceous of China) [26], †*Neosemionotus* Bocchino, 1973 (Early Cretaceous of Argentina) [27], †*Paralepidotus* Stolley, 1920 (Late Triassic of Italy) [28], †*Pliodetes* Wenz, 1999 (Early Cretaceous of Niger) [21], †*Semionotus* Agassiz, 1832 (Late Triassic of Germany) [25], †*Semiolepis* Lombardo & Tintori, 2008 (Middle Triassic of Italy) [29], †*Sinolepidotus* Wei, 1976 (Early Cretaceous of China) [30], †*Tianfuichthys* Su, 1996 (Late Jurassic of China) [31], and †*Tlayuamichin* López-Arbarello & Alvarado-Ortega, 2011 (Early Cretaceous of Mexico) [32]. †*Sangiorgioichthys* Tintori & Lombardo, 2007 (Middle Triassic of Monte San Giorgio, Italy) [33] has recently been removed from †Semionotidae and was placed as incertae sedis within Semionotiformes [34]. The present study is aimed to explore the phylogenetic relationships between the semionotid genera mentioned above and other closely related taxa including lepisosteids and macrosemiids. The homology and evolution of several characters are discussed, and diagnoses and a classification scheme are provided for all monophyletic groups.

### Institutional Abbreviations

**AMNH**, American Museum of Natural History, New York, USA; **BGS.GSM**, British Geological Survey, London, UK; **BSPG**, Bayerische Staatssammlung für Paläontologie un Geologie, München, Germany; **GMPKU**, Geological Museum of Peking University, Beijing, China; **IGM**, Instituto de Geologi?a, Universidad Nacional Auto?noma de Me?xico; **JME**, Jura-Museum Eichstätt, Germany; **MB**, Museum fur Naturkunde, Leibniz-Institut für Evolutions- und Biodiversitätsforschung an der Humboldt-Universität, Berlin, Germany; **MNHN**, Muséum National d’Histoire Naturelle, Paris, France; **NHMUK**, Natural History Museum, London, UK; **SMF**, Senckenberg Forschungsinstitut und Naturmuseum, Frankfurt am Main, Germany.

## Materials and Methods

It was explained in the Introduction that the monophyly of the family †Semionotidae, currently including 11 genera, has never been demonstrated and recent phylogenetic analyses indicate that this assemblage is polyphyletic (Fig. 2) [12]. Therefore, I will refer to this assemblage of 11 genera, as listed in the Introduction, under the informal term “semionotids”, avoiding any assumption of monophyly.

Similarly, the name Semionotiformes has variably been used to refer to different assemblages of genera classified in the family †Semionotidae at different times (e.g. [13], [35]), to a monophyletic clade including such an assemblage plus the families †Macrosemiidae and Lepisosteidae (e.g. [8]–[10], [12], [23], [32], [34], [36], [37]), or to more vaguely defined assemblages of families recalling the original definition of Arambourg & Bertin [38] (e.g. [19], [22], [29], [33]). Pending the definition of a monophyletic Semionotiformes at the end of this study, I will use the term “semionotiforms” to informally refer to all taxa included in the in-group, which are not lepisosteiforms (Lepisosteiformes sensu Grande [13]) or macrosemiids (†Macrosemiidae sensu Bartram [39]).

Grande [13] reorganized some of the genera previously classified in the family Lepisosteidae in a new family †Obaichthyidae. Therefore, I will use the informal name of gars in reference to both lepisosteids and obaichthyids.

### Cladistic Analysis

Phylogenetic relationships are explored through parsimony analysis. A data matrix with a total of 90 characters and 37 taxa was assembled using Mesquite Version 2.73 [40] (see list of material examined in Text S1 and data matrix in Text S2). The data matrix is also availabe in Morphobank (http://www.morphobank.org/). Tree search was performed with PAUP* Version 4.0 beta version [41] and TNT version 1.1 [42]. All characters were considered unordered and given equal weight. All of the studied taxa have been included independently of the amount of missing information (missing data due to lack of information or inapplicable characters varying between 34% for †*Isanichthys* and 3% for *Lepisosteus* or †*Dentilepisosteus*). Most parsimonious trees were obtained both in PAUP* and TNT through heuristic search with random addition sequence, 10000 replicates and tree bisection and reconnection branch swapping. Furthermore, the data matrix was analysed in TNT with the “new technology approaches” (ratchet, sectorial searches, tree drifting, and tree fusing). The number of trees held at each iteration was set at 1 and 10 for different runs with both programs, but the results were identical. Distribution of characters and character changes have been analysed in PAUP* through accelerated and decelerated transformations (ACCTRAN and DELTRAN respectively; see list of synapomorphies in Table S1). Branch support was evaluated through decay indexes for each node (Bremer support) and Bootstrap and Jackknife methods. Both Bootstrap and Jackknife analyses were also run in PAUP* and TNT through heuristic search with 10000 replicates and simple addition sequence.

Based on the results of the cladistic analysis, taxonomic decisions were made within the framework of Phylogenetic Systematics and, thus, the taxa defined herein represent monophyletic groups. All generic diagnoses are based on unambiguous synapomorphies only. To facilitate identifications, additional distinctive combinations of features are also provided. Higher rank taxa are here named based on stem-based definitions according to de Queiroz & Gauthier [43]. The diagnoses proposed for the taxa above the generic rank are based on unambiguous and ambiguous synapomorphies. Among them, the unambiguous synapomorphies are indicated with an asterisk “*” and the ambiguous synapomorphies with “(ACCTRAN)” or “(DELTRAN)” depending on the optimization method (in all cases, the precise direction of change is given in the list of synapomorphies in Table S1). The character number and state is given between brackets for all characters included in the diagnoses.

#### Out-groups

In contrast to previous phylogenetic studies of “semionotiforms” and lepisosteiforms [8], [10], [12], which used a hypothetical ancestor, real outgroup taxa have been used herein: the subholostean †*Perleidus*, two halecomorphs †*Watsonulus eugnathoides* and *Amia calva*, three basal teleosts †*Siemensichthys macrocephalus*, †*Pholidophorus bechei* and †*Leptolepis coryphaenoides*, and †*Dapedium*. The genus †*Perleidus* was erected for †*Semionotus altolepis* Deecke, 1889 [44], a fish from the Middle Triassic of Italy (Upper Ladinian of the Perledo Member of the Perledo-Varenna Formation). Stensiö added the species †*P. woodwardi* Stensiö, 1921 [45], from the Early Triassic of Spitzbergen, and †*P. stoschiensis* Stensiö, 1932 [46], from the Early Triassic of East Greenland. Other species have subsequently been added to this genus by Piveteau [47], Teixeira [48], Lehman [49], Beltan [50], and Su [51]. In a revision of the actinopterygian fishes from the Middle Triassic of northern Italy and the Canton Ticino (Switzerland), Lombardo [52] argued that the genus †*Perleidus* should be restricted to the type species †*P. altolepis*. In particular, †*P. woodwardi* and †*P. stoschiensis* have, according to this author, a very different pattern of bones in the ethmoid region of the skull; †*P. woodwardi* would further differ in having a different kind of caudal fin (abbreviated heterocercal vs. hemiheterocercal in †*P*. *altolepis*). However, apart from differences in the anatomical nomenclature used by Stensiö [45], [46] and Lombardo [52], and the different interpretation of certain bones (in particular the antorbital, interpreted as a rostral by Stensiö) I do not find major differences in the pattern of skull bones in the three species †*P. altolepis*, †*P. woodwardi*, and †*P. stoschiensis*. Quite the opposite, the skull osteology is strikingly similar, supporting the referral of the three species to the same genus. Also, Lombardo [52] argued for the absence of epaxial fin rays in the caudal fin of †*P. woodwardi*, but the caudal fin is not completely preserved in any of the specimens of this species studied by Stensiö and the photograph in Stensiö ([45]: pl. 33) provides no evidence for an heterocercal tail, as indicated by Lombardo ([52]: 357). Consequently, I consider †*P*. *altolepis* and †*P. stoschiensis* as the best described species of †*Perleidus*. Accordingly, the morphological characters were scored on the basis of descriptions by Lombardo [52] and Stensiö [46] and figures of the respective species.

The scorings for the genus †*Watsonulus* are based on the detailed descriptions of the type species †*Watsonulus eugnathoides* (Piveteau, 1935 [47]) published by Beltan [50] and Olsen [53], and the high quality photographs of this species in Grande & Bemis ([54]: figs. 414–417; the syntype MNHN MAE33a and b, and MNHN MAE2506a, MAE2506b, MAE 2507a, MAE2507b, MAE15; YPM 8994; MCZ 13494). The anatomical information for the living species *Amia calva* was taken from the detailed descriptions and excellent illustrations in Grande & Bemis [54] and direct observation on the specimen BSPG 1964-I-400. Developmental information was mainly taken from Allis [55] and Pehrson [56].

The cladistic analysis by Arratia [57] shows that the teleosts split in two lineages at the base of Teleostei. One lineage is represented by the extinct †*Siemensichthys*-Group and the other, leading to the living teleosts, includes †*Pholidophorus* at its base. The genus †*Siemensichthys* Arratia, 2000 [57], was chosen to represent the †*Siemensichthys*-Group [57]. †*Siemensichthys* is represented by two species from the Late Jurassic of Southern Germany: †*S. macrocephalus* (Agassiz, 1834 [58]) and †*S. siemensis* Arratia, 2000 [57]. Among them, the type species †*S. macrocephalus*, originally thought to represent the genus †*Pholidophorus* [58], is the most completely known and, thus, it was chosen to represent the genus in the present cladistic analysis. Scorings for this species are based on Arratia [57] and direct observation of the holotype BSPG AS I 1134. Arratia [57] discussed in detail the problems concerning the poor definition of the order Pholidophoriphormes Berg, 1940 [59], the family Pholidophoridae Woodward, 1890 [14], and even the genus †*Pholidophorus* Agassiz, 1832 [25]. The author demonstrated that a monophyletic †*Pholidophorus* is restricted to the type species †*Ph. latiusculus* and †*Ph. bechei*, as previously suggested by Nybelin [60] and proposed by Zambelli [61]. Scorings for this genus are based on the descriptions of †*Ph*. *bechei* by Nybelin [60], Patterson [62] and Arratia [57], [63]. †*Leptolepis coryphaenoides* is also included because it shares with the more advanced teleosts several synapomorphies that are absent in †*Pholidophorus* or the †*Siemensichthys*-Group [64]. The scorings for †*L. coryphaenoides* are based on Patterson [62] and Arratia [57], [63].

The genus †*Dapedium* is here represented by direct observations on specimens of †*D. pholidotus* (BSPG 1952-XV-603, 1969-I-112), †*D. punctatus* (BSPG 1949-XV-22, 1952-XV-95), †*D. politum* (NHMUK PV P.3555), †*D. colei* (NHMUK PV P.3538, P.4431), and the description and illustrations of an excellently preserved specimen of †*D. coelatus* at the Urweltmuseum Hauff Holzmaden (UHH2 [65]).

To avoid misinterpretations concerning the relationships between the out-group taxa and the ingroup, the analysis was run leaving the outgroup in an unresolved polytomy at the base of the trees. However, due to the possible close relationship between †*Dapedium* and the “semionotiforms”, this genus was not defined as outgroup in PAUP*.

#### In-group

According to recent phylogenetic studies “semionotids”, the gars, and Macrosemiidae form a major monophyletic group [8]–[13] (Figs. 1, 2). Consequently, the three families and a few “semionotiforms” of uncertain relationships are here included in the in-group.

Apart from the three Chinese taxa, which are poorly described in the literature and material of which was not available for this study, the remaining eight of the 11 “semionotid” genera are included in the analysis. Among them, six genera are monospecific: †*Araripelepidotes temnurus* (Agassiz, 1841 [66]), †*Neosemionotus puntanus* Bocchino, 1973 [27], †*Paralepidotus ornatus* (Agassiz, 1833 [58]), †*Pliodetes nigeriensis* Wenz, 1999 [21], †*Semiolepis brembanus* Lombardo & Tintori, 2008 [29], and †*Tlayuamichin itztli* López-Arbarello & Alvarado-Ortega, 2011 [32]. †*Semionotus* Agassiz, 1832 [25], is represented by the type species †*S*. *bergeri* Agassiz, 1832 [25], †*S*. *capensis* Woodward, 1888 [67], and †*S*. *elegans* (Newberry, 1888 [68]), the later considered equivalent to the †*Semionotus elegans* Species Group of Olsen & McCune [8]. These three species were treated as separate OTUs. The genus †*Lepidotes* Agassiz, 1832 [25], is here represented by the type species †*L*. *gigas* Agassiz, 1832 (see section on fist level beta-taxonomy for distinction between this species and †*L*. *elvensis* (Blainville, 1818 [69])) and the very similar †*L*. *semiserratus* Agassiz, 1836 [58]. Other species included in the analysis, which are currently referred to †*Lepidotes* (in alphabetic order †*L*. *laevis*, †*L*. *mantelli*, †*L*. *maximus*, and †*L*. *minor*) probably represent other genera.

Macrosemiids are represented with three genera: †*Macrosemius*, †*Propterus* and †*Notagogus*. The genera previously classified in the family Lepisosteidae have recently been reorganized by Grande [13] in two different families: Lepisosteidae (including *Lepisosteus*, *Atractosteus*, †*Cuneatus*, †*Masillosteus*) and Obaichthyidae (†*Obaichthys* + †*Dentilepisosteus*). Except for †*Cuneatus*, all other lepisosteid and obaichthyid genera are included in the analysis based on the detailed information provided by Grande [13].

Additionally, the four “semionotiforms” of uncertain relationships †*Isanichthys* Cavin & Suteethorn, 2006 [10], †*Sangiorgioichthys* Tintori & Lombardo, 2007 [33], †*Scheenstia* López-Arbarello & Sferco, 2011 [37], and †*Macrosemimimus* Schröder, López-Arbarello & Ebert, 2012 [70] are also part of the in-group. Also, a new Chinese taxon very recently described, *Luoxiongichthys hyperdorsalis* Wen et al. 2012 [71] is here included in the in-group, because, according to my own observations, the fish is a “semionotiform”, although the authors of this taxon classified it in the Halecomorphi.

Detailed information on the studied material and the literature consulted for each taxon is included in Text S1. Most of the taxa included in the in-group have been studied first hand and specific literature was mainly consulted to complete information and reconcile the interpretation of several anatomical features.

### Anatomical Nomenclature

Skull bones are generally named according to the use of most authors in actinopterygians. The bones carrying the infraorbital sensory canal anterior to the orbit are referred to as ‘anterior infraorbitals’ following Wenz [21], [72] and López-Arbarello & Codorniú [22]. The ossifications of the palatoquadrate are named according to Arratia & Schultze [73]. The distinction of non-tritoral, moderately tritoral and strongly tritoral dentitions is based on Jain [74]. Fringing fulcra are named according to Patterson [75]. Scutes, unpaired and paired basal fulcra are identified according to López-Arbarello & Codorniú [22]. More specific problems of anatomical nomenclature related to discussions of homology will be explained in the following section ‘Discussion of characters’.

### Nomenclatural Acts

The electronic version of this document does not represent a published work according to the International Code of Zoological Nomenclature (ICZN), and hence the nomenclatural acts contained in the electronic version are not available under that Code from the electronic edition. Therefore, a separate edition of this document was produced by a method that assures numerous identical and durable copies, and those copies were simultaneously obtainable (from the publication date noted on the first page of this article) for the purpose of providing a public and permanent scientific record, in accordance with Article 8.1 of the Code. The separate print-only edition is available on request from PLoS by sending a request to PLoS ONE, 1160 Battery Street, Suite 100, San Francisco, CA 94111, USA along with a check for $10 (to cover printing and postage) payable to “Public Library of Science”.

In addition, this published work and the nomenclatural acts it contains have been registered in ZooBank, the proposed online registration system for the ICZN. The Zoo Bank LSIDs (Life Science Identifiers) can be resolved and the associated information viewed through any standard web browser by appending the LSID to the prefix “http://zoobank.org/”. The LSID for this publication is: urn:lsid:zoobank.org:pub:BFFD7527-33BA-41D5-AF0F-CFD43625FDBE.

## Results

### Discussion of Characters

Among basal neopterygians, “semionotids” are one of the most morphologically conflicting groups. “Semionotiforms” show morphological affinities with both halecomorphs and teleosts [7], and have been regarded as ancestors of at least some halecostome fishes [1], [2]. Establishing the phylogenetic relationships of these fishes has been a challenge and this is largely due to the poor knowledge of the homology and evolution of several morphological characters.

The 90 parsimony-informative characters used in the present cladistic analysis are listed in this section. Some of the characters are newly proposed, while others are taken from previous authors. In the latter case, the source is clearly indicated. Wiley [76] performed several cladistic analyses of the phylogenetic relationships of gars with other neopterygians, and within the Lepisosteidae. For the purposes of this study, I took characters from his analysis of the relationships of chondrosteans, gars, amiids, and teleosts (indicated with a number followed with “a”), and from his analysis of the relationships of *Lepisosteus* and *Atractosteus* to the Halecostomi and Chondrostei (indicated with a number followed with “b”).

Newly proposed characters or characters significantly modified from previous authors deserve special discussion, and are, thus, explained in detail. Character state “0” does not necessarily represent the plesiomorphic condition because character polarity was determined by rooting the tree [77].

Character 1. Relative position of the dorsal fin.

1. Dorsal fin contained between pelvic and anal fins.
2. Dorsal fin opposite to anal fin.
3. Dorsal fin opposite to pelvic fins.
4. Dorsal fin originates anterior to pelvic fins and extends opposite to anal fin.

Cavin & Suteethorn ([10]: 347) regarded the “elongated body with the dorsal and anal fins located far backward, close to the caudal peduncle” as a synapomorphy shared by gars and †*Isanichthys*. In the latter taxon, the dorsal and anal fins are not as remote as normally in the gars. However, among the studied taxa, only in the gars and †*Isanichthys* are the dorsal and anal fins fully opposite to each other and located backward.

The position of the dorsal fin relative to the pelvic and anal fins is a discrete feature, which is easy to evaluate. Quite the opposite, identifying cylindrical or elongated bodies is usually problematic and rather subjective. †*Pliodetes* specimens are never preserved in lateral view, but in dorsal or dorsolateral view and, thus, this fish apparently shares with the gars a cylindrical body shape. The only known specimen of †*Isanichthys* shows a very long and shallow body, approximately equally deep throughout the thoracic region, suggesting a circular cross section [10]. However, the fish is completely preserved in lateral view, not twisted as usually happen with fishes with circular bodies (like almost all the specimens of †*Pliodetes*) and, thus, the condition in †*Isanichthys* is doubtful. Similar doubts come up when trying to evaluate the condition of a possibly cylindrical body in other fishes with elongated bodies. Evaluating the feature “elongated body” also becomes problematic when trying to draw the line between elongated and not elongated bodies.

Character 2. Posttemporal fossa ([78]: character 33).

1. Absent.
2. Present.

Character 3. Forward extension of the exoccipital around the vagus nerve ([8]: character 3).

1. Absent.
2. Present.

Character 4. Opistotic ([76]: character 6c).

1. Present.
2. Absent.

Character 5. Intercalar ([53]: character 22).

1. Present.
2. Absent.

Character 6. Basisphenoid ([76]: character 17b).

1. Present.
2. Absent.

Character 7. Sphenotic with small dermal component ([13]: character 23).

1. Absent.
2. Present.

Character 8. Posterior myodome ([76]: character 2a).

1. Present.
2. Absent.

Character 9. Elongation of the rostral region anterior to the lower jaw symphysis ([13]: character 4).

1. Extends anterior to the dentary symphysis by less than 20% of mandibular length
2. Extends well anterior to the dentary symphysis by more than 50% of mandibular length.

Character 10. Vomers co-ossified ([53]: character 38).

1. Absent.
2. Present.

Character 11. Autopalatine missing ([76]: character 11b).

1. Absent.
2. Present.

Character 12. Ectopterygoid elongate ([76]: character 10b).

1. Absent.
2. Present.

Character 13. Ectopterygoid participation in palatal surface area ([13]: character 63).

1. Ectopterygoid form half or less of the palatal region.
2. Ectopterygoid forms the majority of the palatal region.

Character 14. Part of dorsal surface of ectopterygoid ornamented and forming part of skull roof ([13]: character 61).

1. Absent.
2. Present.

Character 15. Endopterygoid dentition.

1. Present.
2. Absent.

Endopterygoid bones normally bear teeth in basal neopterygians [73]. Although the condition is unknown in many “semionotiform” taxa, toothless endopterygoids are present in †*Tlayuamichin* and the macrosemiid genera †*Macrosemius* and †*Propterus*.

Character 16. Quadrate position in front of the orbit ([76]: character 13b).

1. Absent.
2. Present.

Character 17. Splint-like quadratojugal (modified from [9]: character 32).

1. Absent.
2. Present and independent.
3. Present and partially fused to the quadrate.
4. Completely fused to the quadrate.

The presence of a quadratojugal is considered primitive in actinopterygians [7]. In basal actinopterygians the quadratojugal is a plate-like dermal ossification placed lateral to the quadrate and tightly bound to the preoperculum, the maxilla and the posterior margin of the quadrate in a very rigid cheek unit (e.g. see detailed descriptions in Gardiner [79] or Arratia & Schultze [73]). In these fishes, the quadratojugal carries a distinctive vertical pit line [73]. The quadratojugal is thus a superficial bone involved in the very rigid upper jaw and the sensory system. Above this primitive level, different conditions are found among neopterygians.

**Figure 3:**
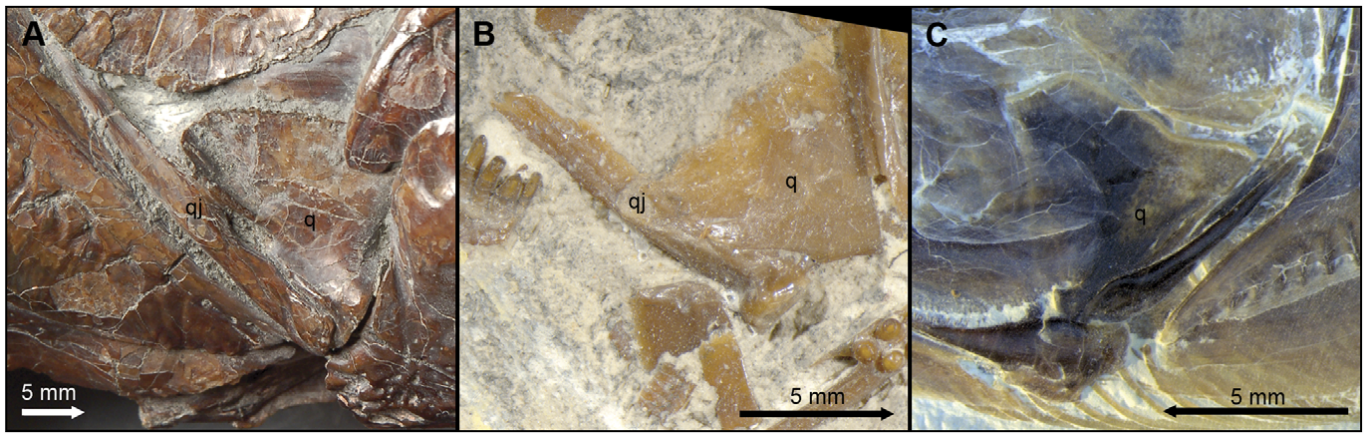
Splint-like quadratojugal. A , Present and independent in †*Macrosemimimus lennieri* (BMNHUK P.25180). **B**, Present and partially fused to the quadrate in †*Macrosemius rostratus* (BSPG AS-I-770). **C**, Completely fused to the quadrate or absent in †*Thrissops formosus* (JME ETT-74). Abbreviations: q, quadrate; qj, quadratojugal. Scale bars point anteriorly.

In “semionotiforms” and †*Dapedium* the bone identified as a quadratojugal is a splint-like dermal ossification lying along the dorsal margin of the preoperculum, with an anterior articular head that buttresses the articular process of the quadrate and a posterior spine-like portion. The symplecticum articulates between the quadrate and this posterior spine-like portion of the quadratojugal. Therefore, the condition in “semionotiforms” (State 1; Fig. 3A) is markedly different from that in basal actinopterygians and this splint-like quadratojugal plays a very different role in the skull. This splint-like bone is well inside the skull and is involved in the suspension of the lower jaw buttressing the palatoquadrate and transmitting forces between the quadrate and the preoperculum.

Although the topographic homology between the plate-like quadratojugal of basal actinopterygians and the splint-like quadratojugal of several neopterygians was proposed by Hammarberg [80] and supported by Patterson [7], it was questioned by Arratia & Schultze [73], who first expressed doubts about the homology among at least some of the different bones identified as quadratojugal in different osteichthyan lineages.

The macrosemiids have a splint-like quadratojugal, the most anterior portion of which is partially fused to the quadrate; the spine-like posterior portion is free (state 2; Fig. 3B) ([39]; pers. obs.). In the gars the quadratojugal is also an independent splint-like bone with an articular head and a spine-like posterior portion, but it is notably larger than in other neopterygians (state 1). In teleosts, there is no independent quadratojugal, but the quadrate forms a spine-like posterior process, which has been considered homologous to the splint-like quadratojugal of “semionotiforms” and other neopterygians [7]. According to this hypothesis of homology, the quadratojugal is completely fused to the quadrate in teleosts (state 3; Fig. 3C).

The homology between the splint-like quadratojugal of *Lepisosteus* and the spine-like posterior process of the quadrate of teleosts has been supported by several authors [7], [39], [76], [81]–[87], but it has been questioned by Arratia & Schultze [73] and Arratia [88]. The similarity between the partially fused quadrate-quadratojugal complex of macrosemiids and the quadrate of basal teleosts is noteworthy (Fig. 3B–C). Strikingly similar is also the development of the quadratojugal of *Lepisosteus* and the posterior process of the quadrate in teleosts. Hammarberg ([80]: p. 315) noted that in *Lepisosteus platostomus* “Das Quadratojugale erscheint im 18.3-mm-Stadium als ein äusserst dünner Knochenstab, der dicht and dem lateroventralen Rand des vorderen Teils des Palatoquadratum gerade hinter dem Unterkiefergelenk liegt” (the quadratojugal appears in the state of 18.3 mm as a very thin rod of bone, which is positioned close to the lateroventral margin of the palatoquadrate, just behind the mandibular joint). In teleosts, the posterior process of the quadrate ossifies independently: “… the posteroventral margin of the pars quadrata … close to the symplectic ossifies first, followed by the membranous ossification of the posterior process; the perichondral ossification of the body of the quadrate follows next” ([73]: pp. 67–68). This early membranous ossification of the posterior process of the quadrate of teleosts further resembles the early ossification of the quadratojugal of *Lepisosteous* both morphologically and topologically (compare [73]: fig. 44B with the description in [80] and the photograph in [13]: fig. 25B). Although I have not found a separate or partially fused quadratojugal in a teleost, I defined an independent character state 3 assuming the homology of the posterior process of the quadrate of teleosts and the splint-like quadratojugal of other neopterygians. Since the character is unordered, this character state 3, which is restricted to the teleosts, does not affect the relationships within the in-group in this analysis, but allows a phylogenetic test for this hypothesis of primary homology. However, since only a few teleosts are here included as out-group taxa, this question of homology cannot be solved in the present phylogenetic study and should be tested in a more comprehensive cladistic analysis of basal neopterygians.

Finally, within Halecomorphi a small plate-like quadratojugal has been identified in one specimen of †*Watsonulus* by Olsen [53] and doubtfully in †*Thomasinotus* by Lehman [49], which would represent a condition similar to that in basal actinopterygians. However, no quadratojugal is present in the specimens of †*Watsonulus* described by Lehman [49] or the acid prepared specimens illustrated by Grande & Bemis [54]. Therefore, and considering that the quadratojugal is absent in all other known halecomorphs [7], [54], the putative quadratojugal in †*Watsonulus* [53] or †*Thomasinotus* [49] might rather represent a different element, probably a preopercular ossification like in †*Prosantichthys* [89].

Chacracter 18. Symplectic involvement in jaw joint ([54]: character 61).

1. Does not articulate with lower jaw.
2. Distal end articulates with articular bone of lower jaw.

Character 19. Ornamentation of the dermal bones of the skull ([13]: character 2, [54]: character 8).

1. Ornamented with tubercles or ridges.
2. Smooth or very slightly ornamented.
3. Ornamented with firmly anchored large conical teeth.

This character is the result of merging character 8 of Grande & Bemis [54] and character 2 of Grande [13]. In the first of these characters Grande & Bemis [54] distinguished between two degrees in the strength of ornamentation of the dermal bones of the skull: weak and/or fine (their character state 0) and strong, coarse (their character state 1). In his character 2 Grande ([13]: 742) distinguished between the presence and absence of “large, firmly anchored, pointed conical teeth covering the dermal bones of the skull”. As shown by Grande [13] this strongly toothed ornamentation is rare among actinopterygians, known only in the Cretaceous gars, in the clupeomorph *Denticeps* and in the †”paleonisciform” †*Coccolepis*.

Character 20. Number of extrascapular bones (modified from [54]: character 49).

1. One pair
2. Two pairs
3. Three or more pairs.

Wiley [76] interpreted the presence of single pair of extrascapulars (vs. two pairs in gars) as a synapomorphy of amiids and teleosts. However, more basal actinopterygians have a single pair of large extrascapular bones, as is the case in †*Perleidus* and †*Watsonulus*.

The number of extrascapular bones within a species might be variable and, thus, the condition should be checked in several specimens when possible. For example, some specimens of †*Lepidotes mantelli* have three pairs of extrascapular bones (NHMUK PV P.6336) while others have four pairs (NHMUK PV P.6933, 11832), and one specimen has three extrascapulars on one side of the skull and four on the other side (NHMUK PV P.20673a). Despite this variability, the patterns defined as the three states of this character were found to be stable within a species among the taxa studied here.

Character 21. Posterior extension of parietals median to the single pair of laterally placed extrascapular bones.

1. Absent.
2. Present.

**Figure 4:**
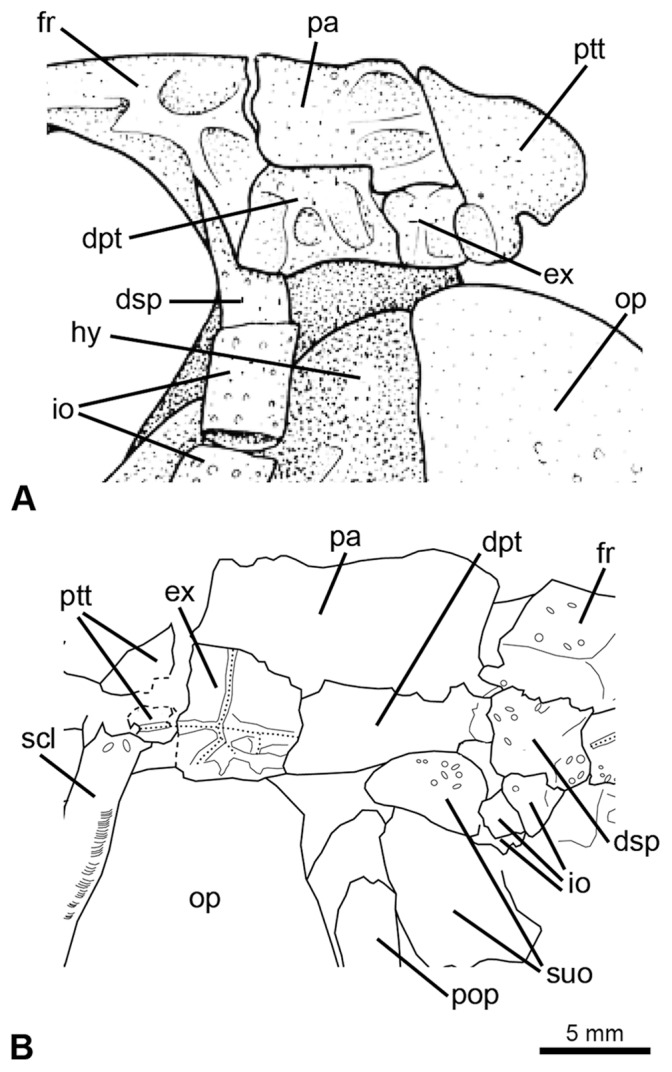
Posterior extension of parietals median to the single pair of laterally placed extrascapular bones. **A**, †*Macrosemius rostratus* (reconstruction based on BSPG AS-I-770; redrawn from [39]). **B**, †*Macrosemimimus fegerti* (JME ETT-854). Abbreviations: dpt, dermopteroticum; dsp, dermosphenoticum; ex, extrascapular; fr, frontal; hy, hyomandibula; io, infraorbital bones; op, operculum; pa, parietal; pop, preoperculum; ptt, posttemporal; scl, supracleithrum; suo, suborbital bone.

In macrosemiids and a few “semionotiforms” the extrascapular bones are represented by one pair of small lateral elements only (Fig. 4) [39]. These bones are placed lateral to the parietals, and the median section of the supraoccipital commissure is enclosed in the posterior portion of the parietal bones. Olsen & McCune [8] interpreted the condition in macrosemiids as homologous with the two pairs of extrascapulars in gars. According to this hypothesis, a fusion of the medial pair of extrascapulars with the parietals is assumed. The fusion of extrascapulars with the parietals has been reported for several taxa (see discussion in Bartram [39]: 143) and it is present in some, though not all specimens of the Chinese species of †*Sangiorgioichthys* [34]. However, no macrosemiid demonstrates direct evidence of this fusion. Even if a fusion is assumed, it is not possible to be certain about the actual number of possibly fused extrascapulars.

Character 22. Relative length of parietals and frontals.

1. Length of parietals less than half but more than one-third the length of frontals.
2. Length of parietals about half the length of frontals.
3. Length of parietals less than one-third the length of frontals.

Character 23. Length of frontals (from [74]; modified from [54]: character 34).

1. Frontals less than 3 times longer than their maximum width.
2. Frontals 3 or more times longer than their maximum width.

Character 24. Frontal bones distinctly broader posteriorly, but long and narrow anteriorly (modified from [88]: character 188).

**Figure 5 pone-0039370-g005:**
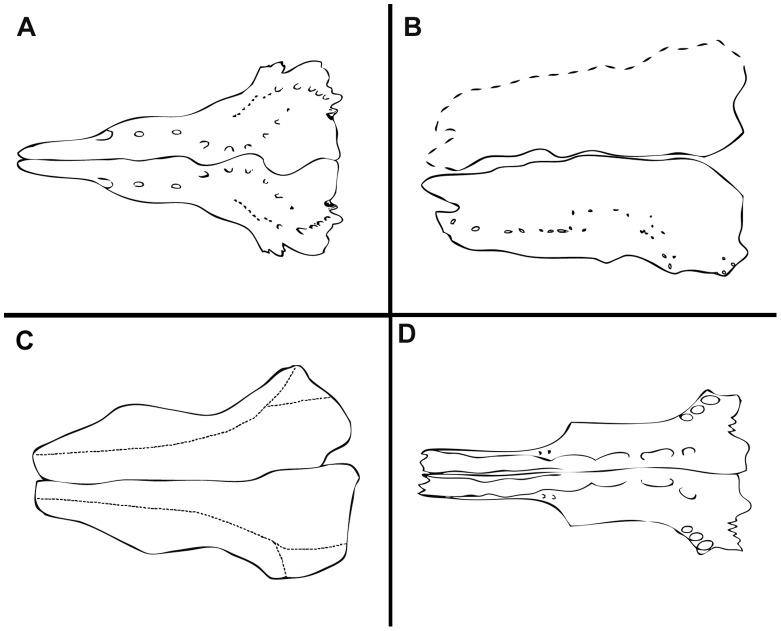
Variation in the shape of the frontal bones in basal neopterygians. A , frontal bones distinctly broader posteriorly, but long and narrow anteriorly; redrawn from [62], fig. 147: restoration of a Callovian species of †*Leptolepis* (Teleostei) based on isolated bones. **B**, Broad antorbital portion of frontal; line drawing of the frontals in †*Lepidotes laevis* MNHN-CRN 61. **C**, Antorbital portion of frontal tapering gradually; line drawing of the frontals in †*Lepidotes minor* NHMUK PV P.1118. **D**, Tubular antorbital portion of frontal; redrawn from [39], fig. 23: line drawing of the frontals in †*Propterus elongatus* BSPG 1964-23-145. Drawn to the same size for comparison.

1. Absent.
2. Present (Fig. 5A).

Character 25. Antorbital portion of frontal.

1. Broad.
2. Tapering gradually.
3. Tubular.

Independently of a more or less developed inter-orbital constriction, the frontals are subrectangular in most basal actinopterygians and in most basal neopterygians (Fig. 5B). In †*Semionotus* and other “semionotiforms” the antorbital portion of the frontal narrows gradually anteriorly (Fig. 5C). In most macrosemiids, including the taxa considered in the present analysis, the frontals narrow abruptly and become almost tubular in the antorbital portion of the skull, enclosing the anterior portion of the supraorbital sensory canal (Fig. 5D) [39]. Outside the †Macrosemiidae, this condition is so far only known in †*Macrosemimimus* ([18]: pl. XIX, fig. B).

Character 26. Frontal ethmoidal sagittal lamina.

1. Absent.
2. Present.

The two species of †*Macrosemimimus* share very peculiarly shaped frontal bones. Anterior to the orbit, each frontal presents a lamina along the sagittal plane, along which it sutures to the nasal process of the premaxilla ([18]: pl. XIX, fig. B; [70]: figs. 4–6, 11). A frontal sagittal lamina is absent in all of the other studied taxa.

**Figure 6 pone-0039370-g006:**
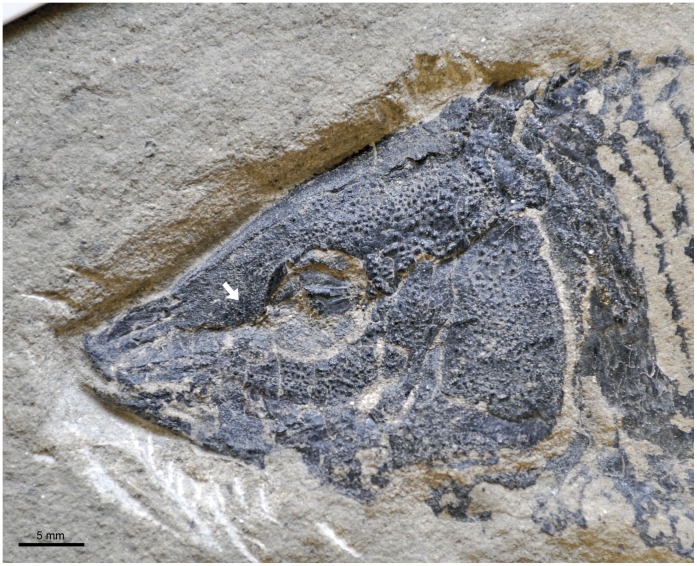
Skull in †*Semionotus bergeri*. White arrow indicates the triangular lateral expansion of antorbital portion of frontal in SMF P6108.

Character 27. Triangular lateral expansion of antorbital portion of frontal.

1. Absent.
2. Present.

In †*Semionotus bergeri*, †*S*. *capensis* and †*Luoxiongichthys* the antorbital portion of the frontal is expanded laterally (Fig. 6). This expanded area has a triangular shape following the anterior rim of the orbit posteriorly and the series of anterior infraorbitals ventrally. Such an expansion is absent in the other studied taxa.

Character 28. Nasals long and narrow.

1. Absent.
2. Present.

The nasal bones are relatively broad in basal neopterygians (e.g. *Amia* [54]), but the bones are narrow and long in many “semionotiforms” (state 1) like †*Sangiorgioichthys* [33], [34] and †*Tlayuamichin* [32]. In †*Lepidotes gigas*, †*Scheenstia* and other semionotiforms the nasal bones are relatively small but broad (state 0) ([37]: fig. 4).

Character 29. Circumborbital ring ([76]: character 9a).

1. Supraorbitals do not contact infraorbitals at the anterior rim of the orbit.
2. Supraorbitals contact infraorbitals, closing the orbit.

**Figure 7 pone-0039370-g007:**
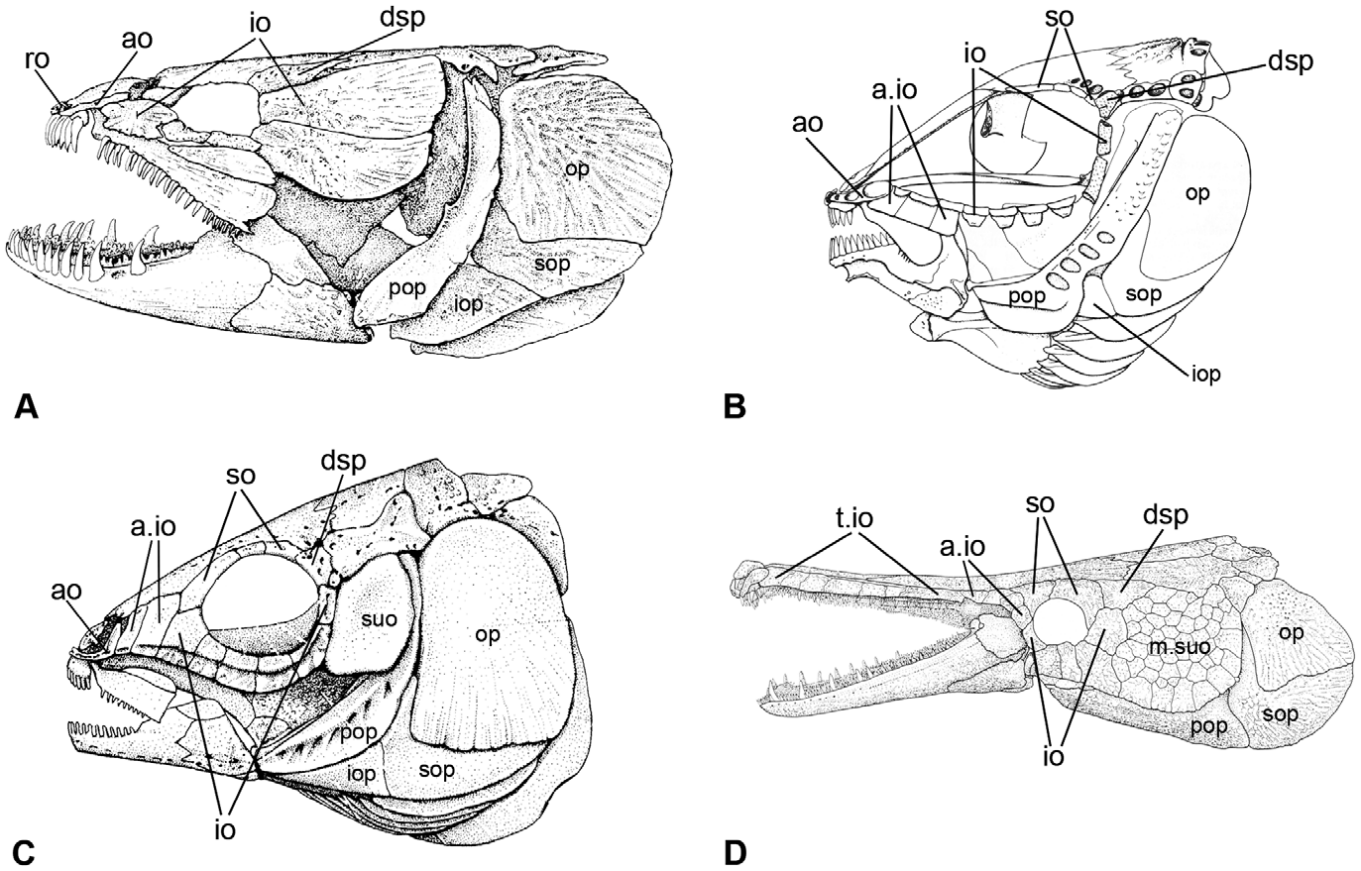
Circumborbital bones in neopterygians. **A**, †*Amia calva* redrawn from Grande & Bemis [54]: fig. 16. **B**, †*Propterus elongatus* redrawn from [39]: fig. 24. **C**, †*Semionotus elegans* redrawn from [8]: fig. 4. **D**, †*Atractosteus spatula* redrawn from [54]: fig. 423. Abbreviations: a.io, anterior infraorbital bone; ao, antorbital; dsp, dermosphenoticum; io, infraorbital bone; iop, interoperculum; m.suo, mosaic of suborbital bones; op, operculum; pop, preoperculum; ro, rostral; so, supraorbital; sop, suboperculum; suo, suborbital; t.io, toothed infraorbital bones.

For this and the following characters related to the circumborbital bones, a brief explanation is necessary concerning the chosen anatomical nomenclature and the homology of certain bones. Starting at the anterodorsal corner of the orbit and in clockwise direction, the following bones are here distinguished in the circumborbital series of the “semionotiforms”, macrosemiids and gars: supraorbitals, dermosphenotic, infraorbitals, anterior infraorbitals, toothed infraorbitals, antorbital, and rostral (Fig. 7). Normally in neopterygians, the circumborbital series includes only supraorbital, dermosphenotic, infraorbital (including the so-called postorbitals, suborbitals and lacrimals), antorbital, and rostral bones (e.g. Fig. 7A). Anterior infraorbitals and toothed infraorbitals are unique features of the “semionotiforms”, macrosemiids and gars (see below), with the latter bones being a unique specialization of the gars [8], [76].

Perleidiforms and other basal neopterygians, as well as a few taxa considered as advanced stem neopterygians have a series of supraorbital bones forming the dorsal rim of the orbit between the nasal and the dermosphenotic [90]. Accordingly, the bones forming the dorsal rim of the orbit and placed lateral to the frontals and anterior to the dermosphenotic are here identified as supraorbitals, though in “semionotiforms”, macrosemiids and gars the skull is elongated anteriorly and, thus, the nasals are far from the orbit and do not articulate with the supraorbital bones (Fig. 7B–D). Under this topographic criterion, the identification of the supraorbital bones largely depends on the identification of the dermosphenotic. Poplin [91] summarized the problems concerning the identification of the dermosphenotic bone in non-teleostean actinopterygians. However, as a single bone placed at the posterodorsal corner of the orbit, laying on the sphenotic, and carrying the last portion of the infraorbital sensory canal, the identification of the dermosphenotic in “semionotiforms”, macrosemiids or gars is usually not problematic (Fig. 7B–D).

Anteroventral to the dermosphenotic follows the series of dermal bones associated with the infraorbital sensory canal, which border the orbit posteriorly and ventrally. Following the dermosphenotic these bones have been named postorbitals and suborbitals in *Amia* (e.g. [54], [56]) and in *Lepisosteus* (e.g. [86]). They were called circumborbitals (e.g. [76], [92]), infraorbitals (e.g. [93]), or subinfraorbitals and postinfraorbitals (e.g. [94]) in gars. In “semionotiforms” and macrosemiids they have generally been named infraorbitals (e.g. [7], [18], [20]–[23], [29], [33], [37], ) but were also called circumborbitals in earlier works (e.g. [95], [96]). Although one or more suborbital, a jugal, and one or more postorbital have been distinguished in this series, the number of infraorbital bones is highly variable among actinopterygians and individual homologies cannot be established [86]. The association of each of these bones with particular neuromasts of the infraorbital sensory line does not provide a valid criterion of homology because the number of neuromasts in this sensory line is variable, even between species of the same genus [85]. Furthermore, their number was shown to be variable between the left and right sides of the same specimen of *L. platostomus* [80].

Developmental studies [56], [80], [85], [97] demonstrated that all the ossifications associated with the infraorbital line occur in connection with one or more neuromasts and go through the same developmental process. Therefore, serial homology (sensu [98]) can be assumed for the whole series from the rostral to the dermosphenotic. Also, some correspondences can be recognized in the development of these dermal ossifications in *Amia* [56] and *Lepisosteus* [80], [85], [97]. The rostral and the antorbital bones appear simultaneously and are among the first elements to ossify. The dermosphenotic appears much later than the rostral and the antorbital, but slightly earlier than one or more infraorbitals immediately below it. The series of infraorbital bones between the antorbital and the dermosphenotic gradually appears in caudally directed succession, starting with the few most anterior elements, which appear concurrently with the rostral and the antorbital.

The most anterior bones in the circumborbital series can further be distinguished because of their relationship with the sensory canals: the rostral with the ethmoidal commissure, the antorbital with the anterior connection between the infra- and supraorbital canals. Similarly, the dermosphenotic, as mentioned before, carries the last portion of the infraorbital sensory canal. Conversely, apart from their sometimes clearly defined position relative to the orbit and their peculiar morphology in some taxa, there is no valid criterion distinguishing individual elements among the infraorbital bones placed between the antorbital and the dermosphenotic. Therefore, taxic primary homology (sensu De Pinna [98]) is here accepted for the rostral, the antorbital and the dermosphenotic individually, and the series of infraorbital bones between the antorbital and the dermosphenotic as a whole.

In “semionotiforms”, macrosemiids and gars, however, the anterior infraorbitals and toothed infraorbitals (Fig. 7B–D) can be distinguished clearly within the series of infraorbital bones, on the bases of their morphology and position. These terms are thus being used to indicate these bones, which are only found in “semionotiforms”, macrosemiids and gars, but individual homologies are not assumed. In the elongated ethmoid region of the skull of these fishes, the series of infraorbital bones starts far beyond the anterior border of the orbit, where it is represented by the so-called anterior infraorbitals and toothed infraorbitals in Lepisosteidae and Obaichthyidae, or by the anterior infraorbitals only in the “semionotiforms”.

The term ‘anterior infraorbitals’ (after [21]) refers to the infraorbital bones placed anterior to the anterior border of the orbit and posterior to the antorbital, which do not contribute to the orbital margin (Fig. 7C). Different names have been used for these bones in the literature: preorbitals [95], lacrimals [8], [76], or anterior infraorbitals [21], [72], among which the latter is preferred here because it highlights the homology of these bones with the other infraorbital bones (serial homology; see above).

The ‘toothed infraorbitals’ (after [76]), are placed between the antorbital and the anterior infraorbitals in lepisosteids and obaichthyids (Fig. 7D). These toothed dermal bones are rigidly attached to the ectopterygoid and pierced by the infraorbital sensory canal [13]. They have been regarded as ‘maxillary bones’ [99], ‘lacrimals’ [80], [85], or ‘infraorbitals’ (Aumonier [97], who proposed their homology with the more posterior infraorbital bones surrounding the orbit [76]). The maxilla, which is extremely reduced, is fused to the most posterior toothed infraorbitals in lepisosteids (at some stage between the 75–150 mm specimens in *L. osseus* and between the 85–125 mm specimens in *L. platostomus*; data from [85]). The number and shape of the anterior infraorbitals is variable among taxa, but stable within a species. The number of toothed infraorbitals varies during the ontogeny [85], and their possible inter- and intraspecific variability in adults is unknown.

Character 30. Ventral border of infraorbital series flexes abruptly dorsally at the anterior margin of the orbit.

1. Absent.
2. Present.

The circumborbital series of bones in lepisosteids and obaichthyids is peculiarly shaped, probably in relation to feeding adaptations [100]. In these fishes, the infraorbital bones at the anterior portion of the orbit become very narrow and the ventral border of the series flexes dorsally rather abruptly, following the orbit and the rounded coronoid process of the lower jaw (Fig. 7D). The lower jaw is then free to effectively move in a rapid strike [100]. A similar pattern of the infraorbital series is otherwise observed in †*Pliodetes*, †*Isanichthys*, †*Scheenstia*, and several species of †*Lepidotes*.

Character 31. Large supraorbital bones.

1. Absent.
2. Present.

Character 32. Most anterior supraorbital bone trapezoidal, longest ventrally, contacting more than one infraorbital bone.

1. Absent.
2. Present.

**Figure 8 pone-0039370-g008:**
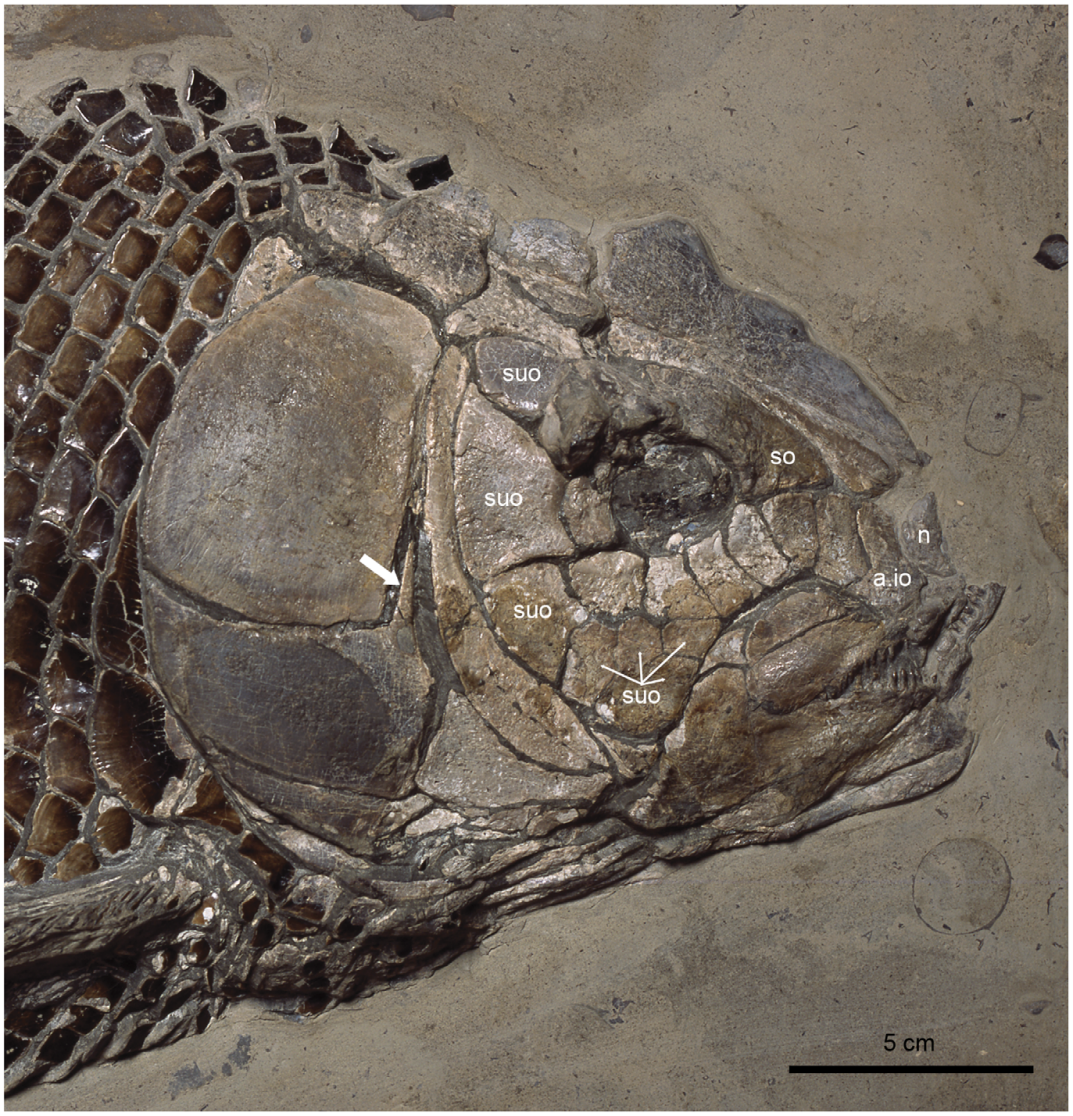
Skull of †*Lepidotes gigas* Agassiz, 1832 [**25**]. Specimen BSPG 1940-I-8 from the area of Holzmaden, Germany.

Generally in basal actinopterygians (e.g. perleidiforms, ophiopsids, macrosemiids; [39], [42], [49], [101], basal halecomorphs [18], [42], [54], [102] and in basal teleosts [88] the supraorbitals are relatively small bones. This is also the case in many “semionotids” (e.g. †*Lepidotes minor* [95]: pl 5, figs. 7–8; †*Semionotus elegans* [8]: figs. 5–6; †*Semionotus bergeri* [23]: figs. 5–6; Fig. 7B–C). However, in other “semionotids” (e.g., †*Lepidotes maximus* [103]: pl. 2; †*Pliodetes* [21]: figs. 5–7), and in the lepisosteids, the supraorbital bones are large and usually the most anterior supraorbital is expanded anteroventrally and articulates with two or three infraorbital bones (Fig. 7D, 8). In †*Scheenstia zappi* ([37]: fig. 4) and †*Neosemionotus* ([22]: figs. 4–6) the supraorbital bones are large, but the anterior supraorbital is not expanded.

Character 33. A series of toothed infraorbitals bordering the snout ([76]: character 3b).

1. Absent.
2. Present (Fig. 7D).

Character 34. Anterior infraorbitals ([8]: character 1).

1. Absent.
2. Present.

**Figure 9:**
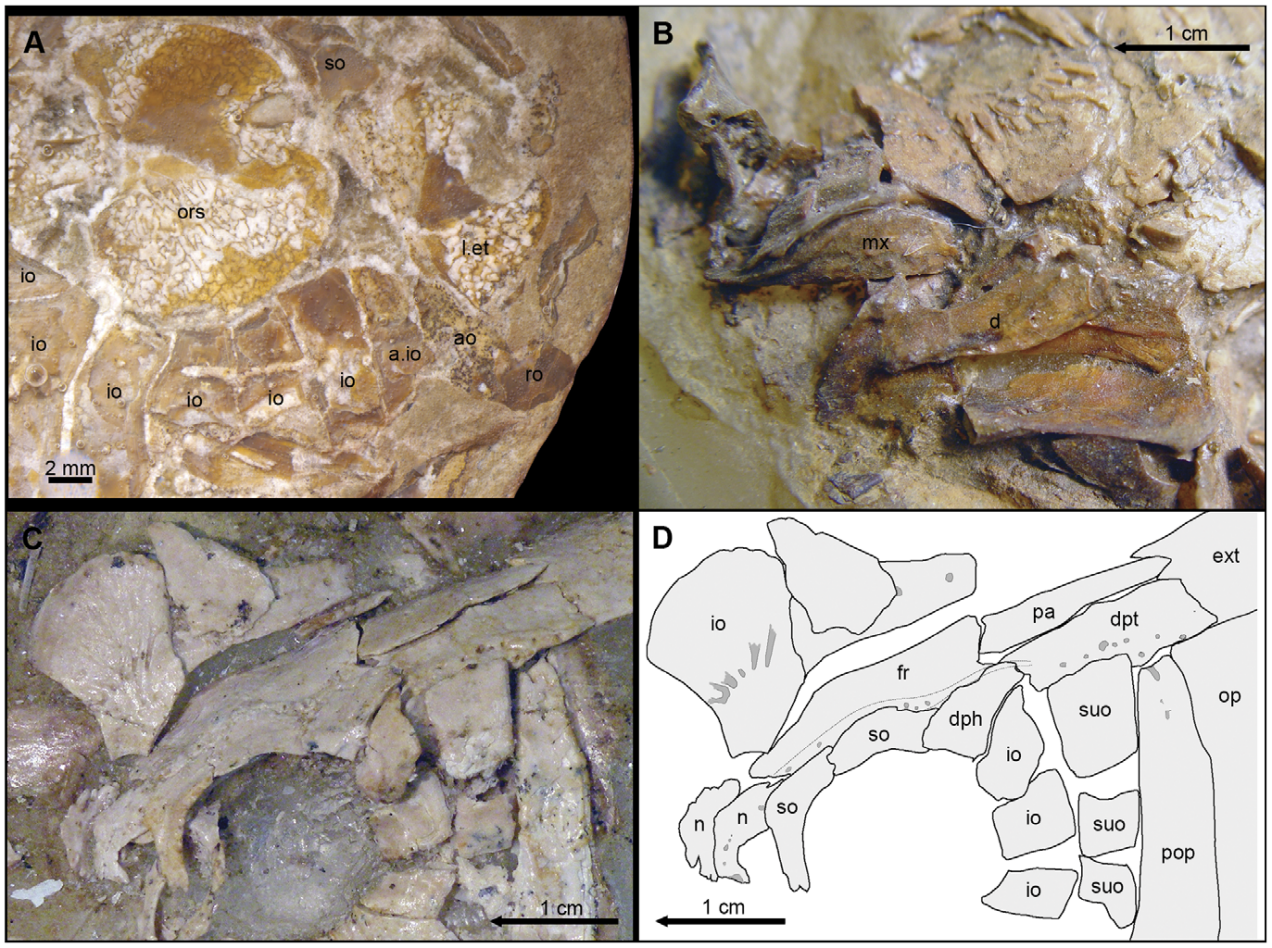
Details of the skull of †*Araripelepidotes temnurus*. A , Anterior portion of the skull in BSPG 1965-I-132 showing the anterior infraorbital. **B**, Upper and lower jaws in MNHN BCE-336. **C** (photograph) and **D** (line drawing), Posterodorsal portion of the skull in AMNH 11833R showing the path of the supraorbital sensory canals. Abbreviations: a.io, anterior infraorbital bone; ao, antorbital; d, dentary; dph, dermosphenoticum; dpt, dermopteroticum; ext, extrascapular; fr, frontal; io, infraorbital bone; l.et, lateral ethmoid; mx, maxilla; n, nasal; op, operculum; ors, orbitosphenoid; pa, parietal; pop, preoperculum; ro, rostral; so, supraorbital; suo, suborbital.

Anterior infraorbitals (Fig. 7B–C) are unknown outside “semionotiforms”, macrosemiids or gars and their presence was proposed as a synapomorphy of the clade formed by these groups by Olsen & McCune [8]. †*Araripelepidotes temnurus* was reconstructed without anterior infraorbitals by Maisey ([104]: 122). However, specimen BSPG 1965-I-132 has a very well-preserved skull in which one anterior infraorbital is clearly visible (Fig. 9A). The variation in the number of anterior infraorbitals in the skull of “semionotiforms”, macrosemiids and gars is high and usually intraspecific.

Although the orbit in the macrosemiids is widely open anteriorly, the position of its anterior rim can be estimated on the basis of the curvature of the other orbital margins and at least three (†*Notagogus*) or four (†*Macrosemius*, †*Propterus*) infraorbital bones are placed in the ethmoid region of the skull in these fishes (Fig. 7B).

Character 35. Most anterior infraorbital.

1. Lower than or equalling the posterior elements.
2. Higher than posterior elements.

In most “semionotiforms”, the dorsal border of the anterior portion of the circumborbital series describes a convex curve, while the ventral border follows an only slightly concave curve. Accordingly, the depth of the anterior infraorbitals decreases gradually anteriorly, so that the most posterior anterior infraorbital is the deepest among these elements. In the macrosemiids the series of anterior infraorbitals is almost straight and, thus, the bones are all approximately equally deep. In contrast, in †*Lepidotes gigas* ([18]: pls 21–22), †*L. semiserratus*, and †*Isanichthys* ([10]: text-fig. 2A), the ventral border of the anterior portion of the circumborbital series follows a deep concave curve and the depth of the anterior infraorbitals becomes gradually larger anteriorly, so that the most anterior infraorbital is the deepest among these elements (Fig. 8).

Character 36. Relative size of the infraorbital bone (or bones) at the posteroventral corner of the orbit.

1. Not enlarged.
2. Enlarged, but do not reach the preoperculum.
3. Enlarged and reach the preoperculum.

The series of infraorbital bones expands at the posteroventral corner of the orbit in some “semionotiform” taxa. In †*Araripelepidotes*, †*Semiolepis* and †*Sangiorgioichthys*, as well as in the lepisosteids and obaichthyids, a single large infraorbital is expanded posteroventrally and contacts the anterodorsal border of the preoperculum. In †*Paralepidotus* and †*Pliodetes* the infraorbital at the posteroventral corner of the orbit is also large and expanded, but does not reach the preoperculum.

Character 37. Shape of the infraorbital bones at the posterior border of the orbit.

1. Deeper than long, sometimes almost tubular.
2. Approximately quadrangular.
3. Longer than deep, expanded posteriorly.

The shape of the infraorbital bones at the posterior border of the orbit is variable in “semionotiforms”. This variability can be summarized in the three character states described above, which account for the condition observed in the studied taxa. In most “semionotiforms”, the infraorbitals forming the posterior border of the orbit are relatively small bones, which are dorsoventrally elongated and sometimes almost reduced to a tube around the infraorbital sensory canal (Fig. 7B–C). In some taxa like †*Lepidotes gigas*, †*Neosemionotus* or †*Scheenstia zappi* these posterior infraorbitals are rather quadrate-shaped bones, approximately as deep as long (Fig. 8; [37]: fig. 4). In several of the out-group taxa like the teleosts, *Amia* and †*Dapedium*, but also in †*Isanichthys* and several “semionotiforms” the posterior infraorbitals are expanded posteriorly ([10]: text-fig. 2A).

Character 38. Dermosphenotic participation in orbital margin ([13]: character 16).

1. Dermosphenotic reaches orbital margin.
2. Dermosphenotic does not reach orbital margin (Fig. 7D).

Character 39. Dermosphenotic/sphenotic association ([13]: character 22).

1. Closely associated with each other (i.e. contacting or fused to each other).
2. Not in contact with each other.

Character 40. Quadrate laterally covered by infraorbital bones.

1. Absent.
2. Present.

In most neopterygians the area of the cheek lateral to the quadrate is naked or protected by suborbital bones (see character 36). In lepisosteids, obaichthyids, †*Pliodetes* and †*Araripelepidotes* however, the series of infraorbital bones expands posteriorly and ventrally, covering the quadrate laterally (Fig. 7D).

Cavin recently defined a comparable character: “Cheek: not complete (quadrate visible) (0); complete (1)” ([12]: character 19). Defined this way, the character implies homology between two different conditions: the quadrate covered by infraorbital bones and the quadrate covered by suborbital bones. However, infraorbital and suborbital bones are not homologous and, thus, these two different conditions are here represented in two independent characters (character 40 and character 42, state 2, respectively).

Character 41. Suborbital bones ([54]: character 7).

1. Present.
2. Absent.

Character 42. Number of suborbital bones (modified from [10]: character 4).

**Figure 10:**
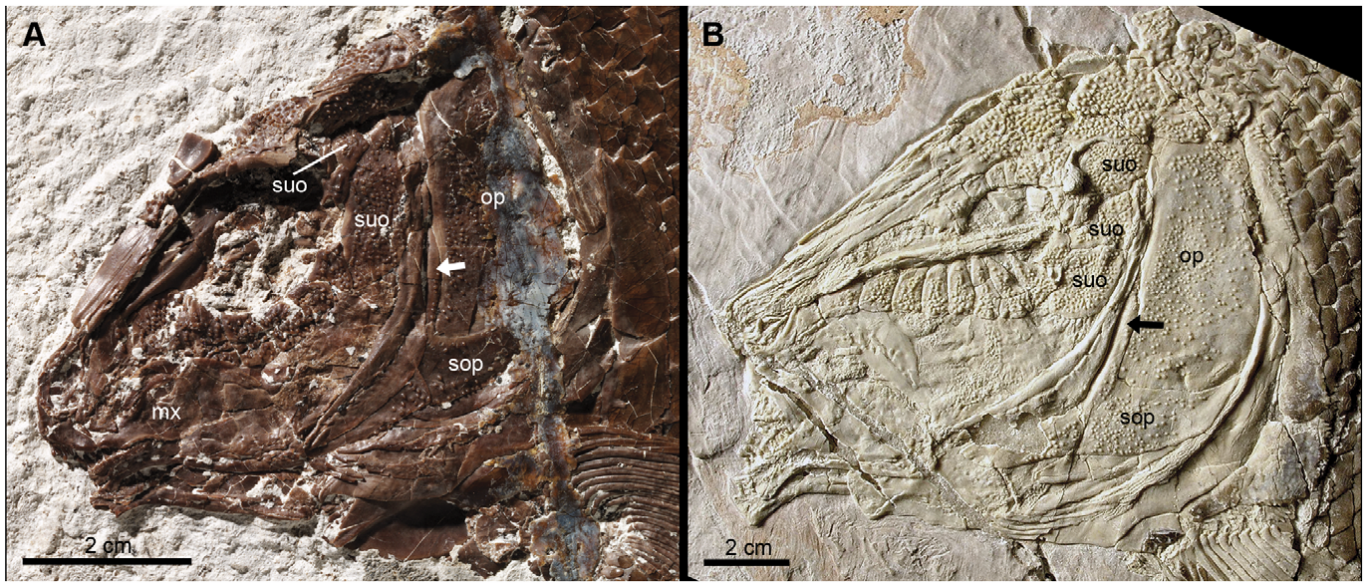
Low number of suborbital bones. **A**, Skull of the neotype of †*Lepidotes minor* (GSM 27975). **B**, Skull of the holotype of †*Tlayuamichin itztli* (IGM 6716). Abbreviations: mx, maxilla; op, operculum; sop, suboperculum; suo, suborbital. Arrows indicate the high ascending process of the suboperculum.

1. One (Fig. 7C).
2. Two (Fig. 10A).
3. Several arranged in one row, which extends anteriorly below the orbit (Fig. 8).
4. Mosaic of numerous suborbitals (Fig. 7D).
5. Three or four suborbitals arranged in a row, which does not extend anteriorly below the orbit (Fig. 10B).

Jain & Robinson [105] and Wenz [21] first attempted to classify the “semionotids” according the number and arrangement of suborbital bones. Wenz [21] presented three character states, which are equivalent to character state 0, 2 and 3 as defined here. Later, Cavin & Suteethorn [10] first included this character in a cladistic analysis using the three character states defined by Wenz and a fourth state representing the absence of suborbital bones. The same character was more recently used in the cladistic analysis of Cavin [12].

The presence or absence of suborbital bones is here represented with a separate character (41), because the presence of suborbital bones is independent of their number and arrangement. On the other hand, two character states have been added to represent the observed variability better. Several taxa have a stable number of two (†*Lepidotes minor* and the two species of †*Macrosemimimus* among the species included in this analysis). On the other hand, †*Araripelepidotes*, †*Neosemionotus* and †*Tlayuamichin* have three or four suborbital bones arranged in a series, but limited to the area posterior to the orbit. In all the fishes presenting the character state 2 (several suborbitals arranged in one row extending below the orbit), and only in these fishes, the suborbitals cover the quadrate bone laterally and, thus, this character state and the character 40 together account for the character 19 of Cavin ([12]; see comments above).

Cavin & Suteethorn [10] and Cavin [12] considered the pattern of suborbitals in †*Isanichthys* equivalent to the mosaic of suborbitals present in other taxa like the lepisosteids because there are two rows of suborbitals in the ventral region of the cheek in this fish. However, at least one specimen of †*Lepidotes gigas* (BSPG 1940-I-8; Fig. 8) and one specimen of †*L*. *elvensis* (MNHN JRE-250), species that normally present a single series of suborbital bones, also have irregularly arranged suborbitals in the ventral region of the cheek. Therefore, the pattern in †*Isanichthys* is here considered a normal deviation from character state 2.

Similarly, although in most specimens of †*Sangiorgioichthys sui* the series of suborbital bones is interrupted by an enlarged infraorbital that reaches the preoperculum, thus separating the suborbitals placed posterior to the orbit from the one or two elements placed lateral to the quadrate, a few specimens show a continuous series of suborbitals, like in the cases represented by the character state 2 ([34]: fig. 5; specimens GMPKU-P-1359 and GMPKU-P-1973]). The series of suborbitals is also continuous, though narrowed by the presence of a large infraorbital, in two specimens of †*S*. *aldae* figured by Tintori & Lombardo ([33]: figs. 3–4).

The number of suborbital bones is much lower in †*Obaichthys* (two or three) and †*Dentilepisosteus* (three or four) than in the lepisosteids. However, since these few suborbitals are irregular in shape and size and irregularly arranged [13], the condition in the two obaichthyid genera is considered here homologous to the mosaic of suborbitals normally present in the gars.

Character 43. Independent of the total number, there is a large suborbital covering almost the whole area between the infraorbital bones and the preoperculum.

1. Absent
2. Present

Independent of the total number of suborbital bones, different patterns of suborbitals have been observed in those fishes with more than one suborbital bone. In gars and the “semionotiforms” with a mosaic of suborbitals, the suborbial bones are irregular in size and shape and no pattern can be defined, apart from the mosaic itself (state 0). However, three patterns steadily repeat in those fishes with more than one suborbital arranged in a row. In fishes such as †*Lepidotes minor* or †*Macrosemimimus*, the first (most dorsal) suborbital is relatively small, ovoid to subrectangular in shape and longitudinally elongate, and the second is notably the largest in the series and covers almost the whole area between the infraorbital bones and the preoperculum (Fig. 10A). This pattern also occurs in †*Sangiorgioichthys*, although this fish has a series of suborbitals arranged in one row.

Character 44. First and last suborbitals are larger than the other suborbitals.

1. Absent
2. Present

**Figure 11:**
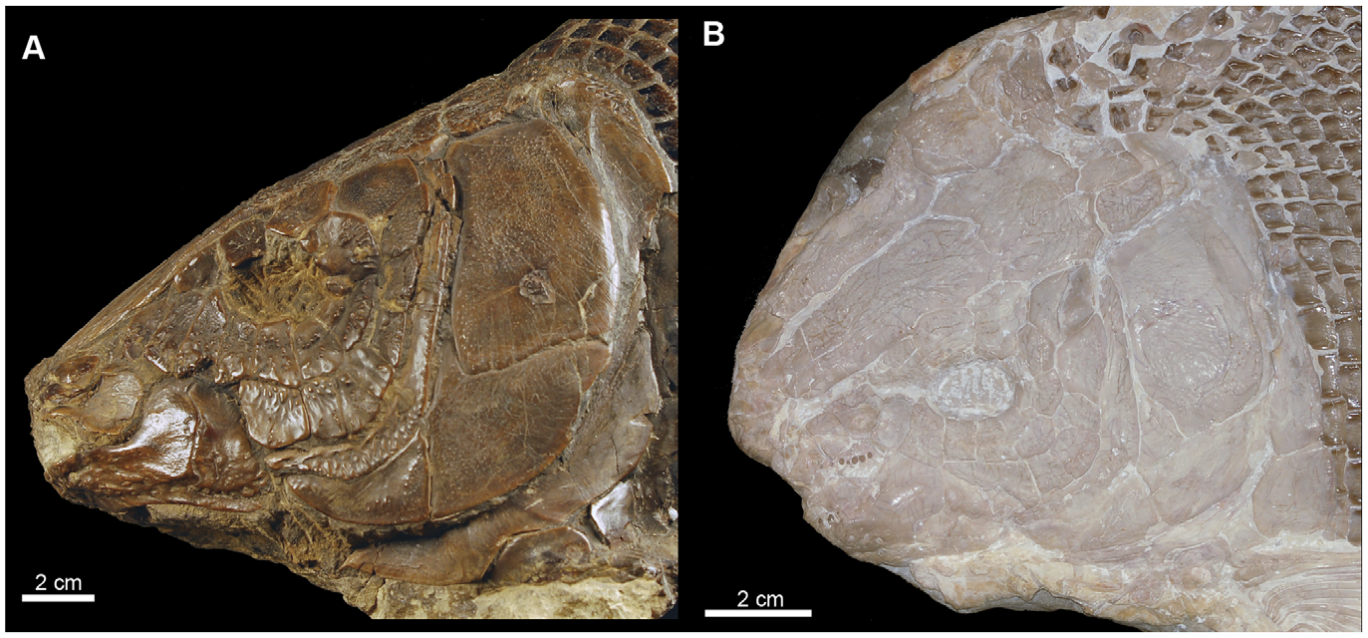
Skull in two large tritoral species. **A**, †*Lepidotes mantelli* (NHMUK PV P. 6933). **B**, †*Lepidotes laevis* (MNHN-CRN 61).

In †*Scheenstia zappi* and the large, tritoral forms referred to †*Lepidotes* (†*L*. *mantelli*, †*L*. *maximus*, and †*L*. *laevis*), there is a series of suborbitals extending forwards ventral to the orbit. In this series, the first and last suborbitals are larger than the other suborbital bones, which might be variable in size and shape (Fig. 11; [103]: pl. 2; [37]: fig. 8).

Character 45. Suborbital series separates preoperculum from dermopterotic.

1. Absent
2. Present

In †*Tlayuamichin* and †*Sangiorgioichthys aldae* the first and most dorsal suborbital separates the preoperculum from the dermopterotic (Fig. 10B; [32]: figs. 6–9). At least in †*Tlayuamichin itztli* this suborbital is traversed by the preopercular sensory canal ([32]: fig. 9C).

Character 46. Triangular suborbital lateral to quadrate.

1. Absent.
2. Present.

The species of †*Sangiorgioichthys* share the presence of one or two suborbital bones covering the quadrate laterally ([33]: figs. 3–4; [34]: figs. 4–5).

Character 47. Premaxilla with nasal process (modified from [8]: character 4).

1. Absent.
2. Present.

Olsen & McCune [8] considered the elongate nasal process of “semionotiforms”, macrosemiids, gars, and *Amia* as a derived condition. Developmental evidence summarized by Wiley [76] suggests that the nasal processes of the premaxillae of *Amia* and gars are derived independently. However, due to the presence of a nasal process in most extinct halecomorphs and “semionotiforms”, for which ontogenetic or developmental data are not available, the homology between the nasal process of gars and *Amia* should be tested in a cladistic analysis. Testing this hypothesis of homology is, however, not the purpose of the present analysis, since it would require a different data set including a much wider array of halecomorphs and other basal neopterygians. Therefore, pending further research, the homology of the nasal processes in all neopterygians is here assumed.

Favouring this assumption of homology, Patterson [7] pointed out the morphological, topographical and functional similarities of the nasal process of gars and *Amia*. In these fishes and in “semionotiforms” the nasal process lines the nasal pits, sutures with the frontal, and is perforated by the olfactory nerve.

Character 48. Premaxillary nasal process forming an external dermal component of the skull roof ([76]: character 5b).

1. Absent.
2. Present.

Character 49. Supraorbital canal in premaxillary nasal process ([76]: character 4b).

1. Absent.
2. Present.

Character 50. Length of maxilla.

1. Long, extends backwards lateral to the coronoid process of the lower jaw.
2. Short, does not reach the coronoid process.
3. Atrophied or absent.

The shape and relative length of the maxilla is variable among “semionotiforms”, though in most cases it is relatively long extending lateral to the coronoid process of the lower jaw (state 0: Figs. 8, 10; e.g., †*Lepidotes gigas*, †*L. semiserratus*, †*Isanichthys*, †*Neosemionotus*, †*Sangiorgioichthys*). In †*Lepidotes mantelli* and †*L. laevis*, and in the Cretaceous gars †*Obaichthys* and †*Dentilepisosteus* the maxilla is short, ending anterior to the coronoid process of the lower jaw (state 1; Fig. 11). In †*Araripelepidotes* and †*Pliodetes*, the maxilla is very reduced but it is still an independent bone with a well-developed articular process (state 1; Fig. 9B). In the lepisosteids the maxilla is atrophied and fused to the “toothed infraorbitals” (State 2) [76], [92].

The jaws of †*Araripelepidotes* are very peculiarly shaped [19]. They are well preserved and nicely exposed in the acid prepared specimens MNHN BCE-335 and BCE-336 (Fig. 9B). In these two specimens, the maxilla is a relatively small bone, the main body of which is laterally compressed, with convex dorsal and posterior borders, and a concave ventral border in MNHN BCE-335, but notably straight ventral border in MNHN BCE-336. The maxilla becomes rapidly shallower and laterally expanded anteriorly forming a dorso-ventrally compressed and anteriorly rounded medial process.

Character 51. Depth of maxilla.

1. Shallow (Figs. 7B–C, 8).
2. Deep (Figs. 10, 11).

The maxilla of “semionotiforms” is normally elongate, its depth being no more than half of its length. In a few taxa however, the maxilla is posteriorly expanded forming a deep plate (e.g. †*Lepidotes mantelli*, †*L. laevis*, †*L. minor*).

Character 52. Supramaxilla ([76]: character 3a).

1. Absent.
2. Present, single bone.
3. Present, two bones.

Character 53. Maxillary teeth ([12]: character 30).

1. Present.
2. Absent.

Character 54. Plicidentine ([76]: character 27b).

1. Absent.
2. Present.

Character 55. Tritoral dentition (from [74]).

1. Absent.
2. Moderately tritoral.
3. Extremely tritoral.

Character 56. Well-developed posteroventral process of the dentary (from [19]).

1. Absent.
2. Present.

This character is taken from Thies [19] and refers to the acuminate process extending backwards from the ventral border of the dentary in †*Lepidotes* and other “semionotiforms”. Cavin & Suteethorn ([10]: character 5) modified this character and considered the condition of the dentary of †*Araripelepidotes* as homologous to the condition in †*Lepidotes* as described by Thies [19]. However, the authors do not discuss this hypothesis in any detail and there is no comparable morphological structure or any evidence supporting the homology of the highly modified dentary of †*Araripelepidotes* (or any portion of it; Fig. 9B; [106]) with the posteroventral process of the dentary in other “semionotiforms”.

The character was further modified by Cavin [12] by adding a character state 2 representing the condition of the dentary of gars. However, there is no evidence of homology for the condition in gars, the dentary of which extends to the posterior border of the lower jaw dorsal to the angular, and the condition in “semionotiforms” as defined here and described by Thies [19], which refers to a process extending backwards ventral to the angular. Only in †*Dentilepisosteus*, in addition to the expanded portion dorsal to the angular that normally occurs in gars, there is a short posteroventral process ([13]: fig. 488), which closely resembles the posteroventral process of the dentary in †*Lepidotes* and it is thus here considered homologous to the latter.

Character 57. Double row of teeth in dentary (modified from [13]: character 39).

1. Absent.
2. Present.

Character 58. Mandibular symphysis very deep (from [74]).

1. Absent.
2. Present.

Character 59. Extent of teeth on dentary (excluding coronoid toothplates) ([13]: character 56).

1. Tooth row extends over a third the length of dentary.
2. Tooth row is present on only the anterior one third or less of dentary.

Character 60. Shape of preoperculum.

1. Dorsoventrally elongated without anteroventral arm.
2. Crescent-shaped.
3. L-shaped.

**Figure 12:**
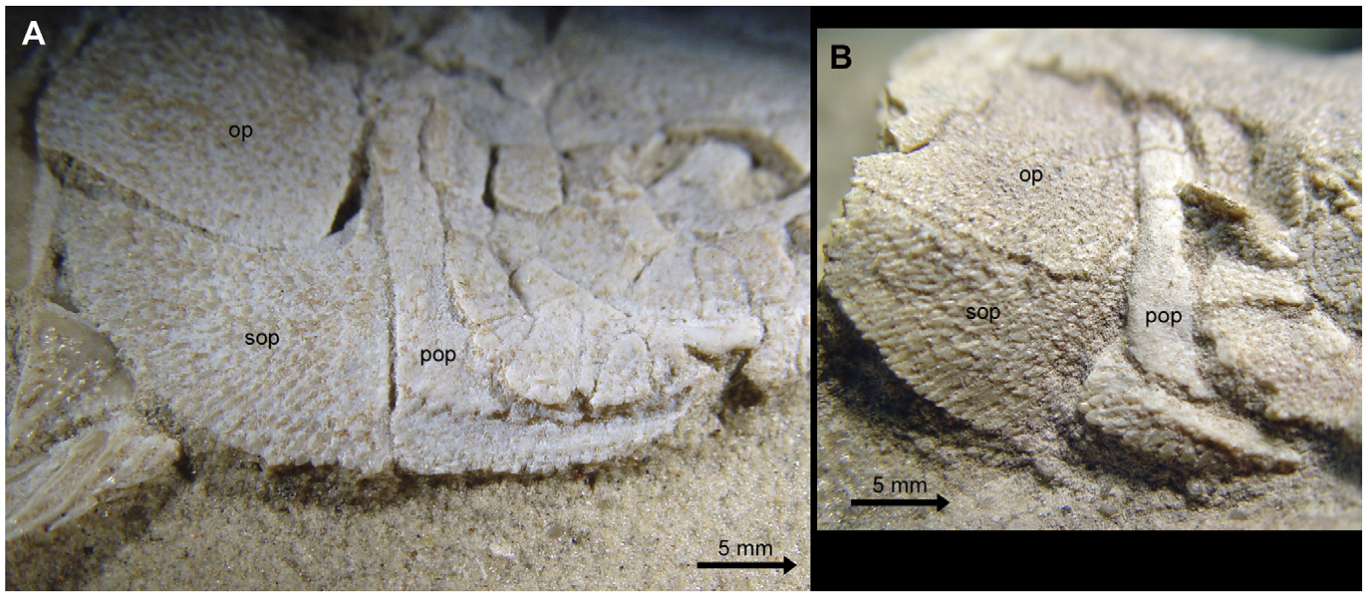
Opercular bones in †*Pliodetes nigeriensis*. **A**, Holotype specimen MNHN GDF-1275. **B**, specimen MNHN GDF-1276 showing detached ventral arm of preoperculum. Abbreviations: op, operculum; pop, preoperculum; sop, suboperculum. Scale bars point anteriorly.

**Figure 13:**
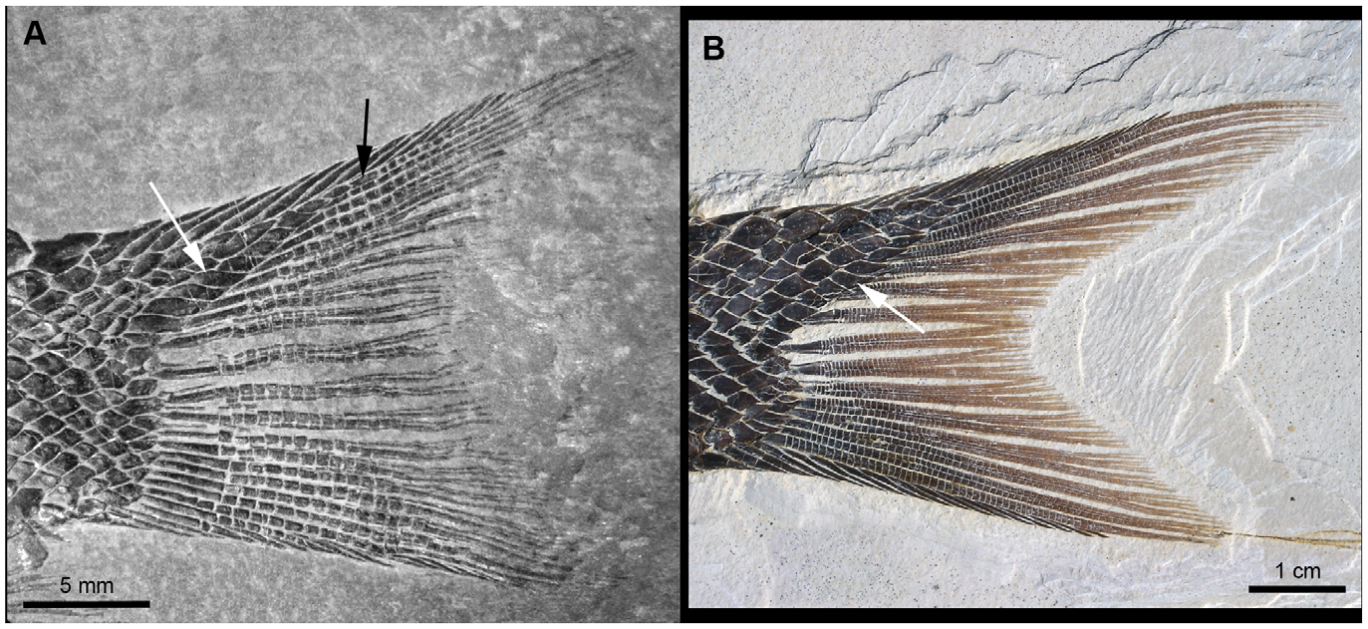
Caudal fin in two ginglymodians. **A**, †*Sangiorgioichthys sui* (GMPKU-P-1359). **B**, †*Macrosemimimus fegerti* (JME ETT-1351). White arrows indicate the marginal row of scales in the body lobe. The black arrow indicates the scale-like ray.

In the out-groups †*Perleidus* and †*Watsonulus*, and in †*Araripelepidotes* the preoperculum is a dorsoventrally elongated bone, which has no anteroventral arm (state 0; see [19]: figs. 1–2). In most “semionotiforms”, as well as in *Amia* and basal teleosts, the preoperculum is a crescent-shaped bone and there are no well-defined dorsal and anteroventral arms (state 1; Fig. 7A–C). Distinctively in †*Pliodetes*, the preoperculum is L-shaped, with well defined dorsal and anteroventral arms forming an approximately right angle (state 2, Fig. 12; see [21]: figs. 5–7). The condition in gars resembles that of †*Pliodetes*, but the dorsal arm is variably reduced in the different taxa, and the anteroventral arm is notably larger than in “semionotiforms” (Fig. 7D; [13]).

Character 61. Exposure of dorsal limb of preoperculum ([13]: character 73).

1. Mostly exposed forming a significant part of the ornamented lateral surface of the skull anterior to the operculum.
2. Entirely covered or nearly entirely covered by other dermal bones in adults (Fig. 7D).

Character 62. Posterior border of preoperculum notched ventrally.

1. Absent.
2. Present.

The posterior border of the preoperculum in †*Macrosemius*, †*Propterus*, †*Notagogus* and other macrosemiids is peculiarly excavated ([39]: figs. 24, 26, 38–39; Fig. 7B). Such a notch is absent in the preoperculum of other “semionotiforms” studied here.

Character 63. Shape of the operculum.

1. Subrectangular, deeper than long.
2. Rounded to quadrate, approximately as deep as long.
3. Tapering anteroventrally.

Although the shape of the operculum is very variable among actinopterygians, it is typically subrectangular, deeper than long, in most “semionotiforms” (state 0; Fig. 7A–C). In the gars and in †*Pliodetes* and †*Araripelepidotes*, the operculum is rounded and approximately as deep as long (state 1; Figs. 7D, 12). In the teleosts the ventral portion of the operculum typically narrows in anteroventral direction (state 2).

Character 64. Suboperculum with well-developed ascending process.

1. Absent.
2. Present.

In “semionotiforms” the suboperculum has a well-developed ascending process, which is absent in non-neopterygian actinopterygians (Figs. 7, 10, 11). The distribution of this character among neopterygians is poorly known, although an ascending process is present in †*Dapedium* and *Amia*.

Character 65. Shape of ascending process of the suboperculum.

1. Robust, with broad base and rounded distal end (Figs. 7A, 11B).
2. Slender, tapering dorsally (Figs. 7B–C, 8, 10, 11A).

The shape and relative height of the ascending process of the suboperculum is variable among “semionotiforms”. The ascending process is usually narrow and acuminate towards the dorsal tip in most cases, but it is unusually broad and with rounded dorsal end in †*Lepidotes maximus*, †*L*. *laevis*, the lepisosteids, and †*Dentilepisosteus*.

Character 66. High ascending process of the suboperculum.

1. Less than or equal to half of the length of the dorsal border of the bone (Fig. 8).
2. More than half of the length of the dorsal border of the bone (Fig. 10).

In addition to the variation in shape, the height of the ascending process is usually less than half of the length of the dorsal border of the suboperculum in most taxa, but it is unusually high in †*Lepidotes minor*, †*Tlayuamichin*, †*Macrosemimimus*, †*Paralepidotus*, and †*Semiolepis*.

Character 67. Suboperculum less than half the depth of the operculum.

1. Absent (Fig. 7D, 12).
2. Present (Figs. 7A–C, 8, 10, 11).

The depth of the suboperculum is normally less than half of the depth of the operculum, but the bone is deeper in most of the taxa with shallow opercula (character 54). Although characters 54 and 58 are based on relative measurements, the two characters are independent and the suboperculum is relatively shallow in †*Araripelepidotes* and †*Obaichthys*, although the operculum in these taxa is approximately as deep as it is long.

Character 68. Interoperculum (modified from [76]: character 10a).

1. Absent.
2. Present.

The presence of an interoperculum is a synapomorphy of Neopterygii. The bone has been secondary lost independently in Lepisosteidae and Siluridae (Teleostei). Wenz [21] mentioned the presence of an interoperculum in †*Pliodetes*. However, after detailed observation of the specimens of †*Pliodetes* in the MNHN (Paris), there is no independent interoperculum in this fish. The preoperculum of †*Pliodetes* is a robust L-shaped bone, which is firmly attached to the suboperculum. The preopercular canal is deeply excavated close to the anterior and dorsal margin of the preoperculum, and several branches of the main canal exit the bone through a series of relatively large pores aligned almost parallel to the dorsal border of its ventral (horizontal) arm (se holotype MNHN GDF-1275 in Fig. 12A). In some specimens, the ventral arm ventral to this series of pores is detached from the rest of the bone, thus resembling and independent interoperculum (e.g. MNHN GDF-1276 in Fig. 12B).

The presence of an independent interoperculum in obaichthyids has been clearly illustrated by Grande ([13]: figs. 473C, 476, 488, 490).

Character 69. Size of interoperculum.

1. Large, approximately as long as the ventral arm of the preoperculum.
2. Small, remote from mandible.

The interoperculum is longitudinally elongated, deepest posteriorly at the suture with the suboperculum, and narrowing gradually in anterior direction. It places medial and ventral to the preoperculum and usually extends all along the horizontal arm of the latter (state 0; Figs. 7A, C, 8, 10, 11). Thus, the anterior border of the interoperculum is close to the posterior end of the lower jaw, to which it is connected through a ligament in Recent fishes. Bartram [39] noted that the interoperculum in macrosemiid fishes is smaller than usual and places well behind the lower jaw (state 1; Fig. 7B). The same condition was observed in some “semionotiforms” like †*Neosemionotus* or †*Semiolepis*.

Character 70. Gular plate (modified from [8]: character 8).

1. Double.
2. Single.
3. Absent.

Character 71. Opistocoelous vertebrae ([76]: character 26b).

1. Absent.
2. Present.

Character 72. Knob-like anteroventral process of posttemporal.

1. Absent.
2. Present.

The posttemporal bone in †*Scheenstia* and the large tritoral †*Lepidotes maximus*, †*L. laevis*, and †*L. mantelli* forms a stout distinct knob-like anteroventral process ([37]: fig. 6).

Character 73. Supracleithrum with a concave articular facet for articulation with the posttemporal ([13]: character 93).

1. Absent.
2. Present.

Character 74. Series of denticles along the ridge between the branchial and lateral surfaces of the cleithrum (from [39]).

1. Absent.
2. One or two rows.
3. Several rows.

Several of the studied taxa present one or two series of very small denticles aligned between the branchial and lateral surfaces of the cleithrum (e.g. [8]: 11; [23]: fig. 7). In †*Lepidotes gigas*, †*L. minor*, †*Tlayuamichin* (Fig. 10B), †*Sangiorgioichthys*, †*Scheenstia* and several other taxa, these denticles are arranged in several rows. On the other hand, such denticles are absent in the gars.

Character 75. Fringing fulcra on pectoral fin.

1. Present.
2. Absent.

Character 76. Fringing fulcra on pelvic fin.

1. Present.
2. Absent.

Character 77. Large dorsal fin, with more than 20 rays.

1. Absent.
2. Present.

Character 78. Large basal fulcra in the dorsal and anal fins.

1. Absent.
2. Present.

Character 79. Scale-like ray at the dorsal margin of the caudal fin (from [39]).

1. Absent.
2. Present.

Bartram ([39]: 218) discussed the peculiar condition of the uppermost caudal fin ray in macrosemiids, which “does not insert beneath the squamation proximally, but remains superficial, and is not sharply delimited from the axial lobe scales”. He considered this condition as primitive and reported the same phenomenon in *Lepisosteus osseus* and †*Acentrophorus varians*, and partially in †*Dapedium orbis* and a species of †*Caturus*. Such a scale-like ray is also present in several “semionotiforms” (Fig. 13A).

Character 80. A constant number of exactly eight lepidotrichia in the lower, non-axial lobe of the tail (from [39]).

1. Absent.
2. Present.

Another interesting feature observed by Bartram ([39]: 219) in the caudal fin of macrosemiids, is the “constancy of number of the eight lower, non-axial lobe rays”. Several “semionotiforms” also present a constant number of eight rays in the lower lobe of the caudal fin (e.g. †*Semionotus*, †*Tlayuamichin*, †*Macrosemimimus*; Fig. 13B).

Character 81. A constant number of exactly six lepidotrichia in the lower, non-axial lobe of the tail.

1. Absent.
2. Present.

Resembling the case described before, the gars also present a constant number of rays in the lower lobe of the caudal fin, but there are six in this case. Comparing the specimens of *Lepisosteus osseus* illustrated in the figures 89 and 94 in [13], the lateral line and the hinge-line or limit of the body lobe are good indicators of the limit between the upper caudal fin rays, which articulate with the hypurals, and the lower caudal fin rays, which articulate with the parhypural and precaudal haemal spines.

Character 82. Body lobe scale row (modified from [29]).

1. Absent.
2. Present, with additional incomplete row.
3. Present, without additional incomplete row.

Lombardo & Tintori ([29]: fig. 8) first noticed the variation related to the row of scales bordering the axial lobe of the tail in some “semionotiforms”. In these fishes, like †*Sangiorgioichthys sui*, there is a complete row of elongated scales between the last scale of the lateral line and the uppermost caudal fin ray (Fig. 13A). In †*Macrosemimimus fegerti* and other taxa like †*Tlayuamichin* or †*Semiolepis*, there is an incomplete row of scales at the margin of the body lobe, in addition to the complete row described before (Fig. 13B). In other “semionotiforms” like †*Lepidotes* or †*Scheenstia* and in the gars, several more or less well-defined incomplete rows of scales form the margin of the body lobe.

Character 83. Dorsal ridge of scales (modified from [8]: character 17).

1. Inconspicuous.
2. Conspicuous, with a low spine.
3. Conspicuous, with a high spine.

Character 84. Scales of the body with a strong posteriorly directed spine.

1. Absent.
2. Present.

**Figure 14:**
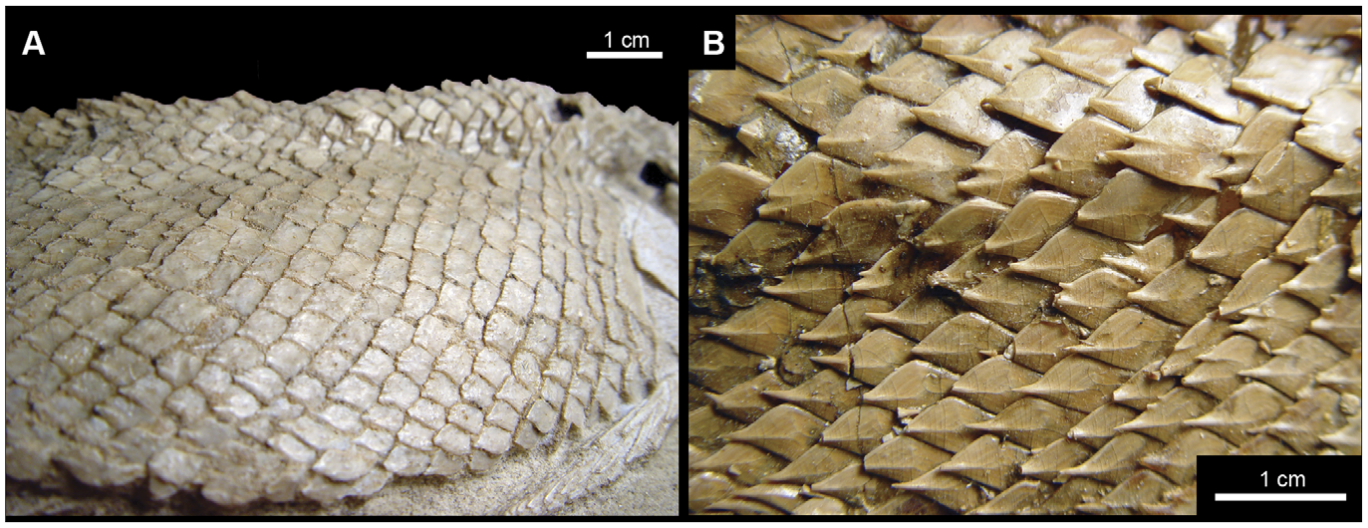
Body scales with strong posterior spine. **A**, †*Pliodetes nigeriensis* (MNHN GDF-1275), **B**, †*Araripelepidotes temnurus* (MNHN BCE-335).

Independently of the presence or absence of other serrations, the scales of the body in †*Pliodetes*, †*Araripelepidotes* and the Cretaceous gars †*Dentilepisosteus* and †*Obaichthys* have a very strong spine protruding from the posterior border in caudal direction (Fig. 14). In the first three genera, the spine protrudes from the posteroventral corner of the scale, while in †*Obaichthys* it protrudes from the posteroventral corner or from the middle of the posterior border of the scale ([13]: fig. 479H).

Character 85. Vertical peg-and-socket articulation.

1. Present.
2. Reduced or absent.

**Figure 15:**
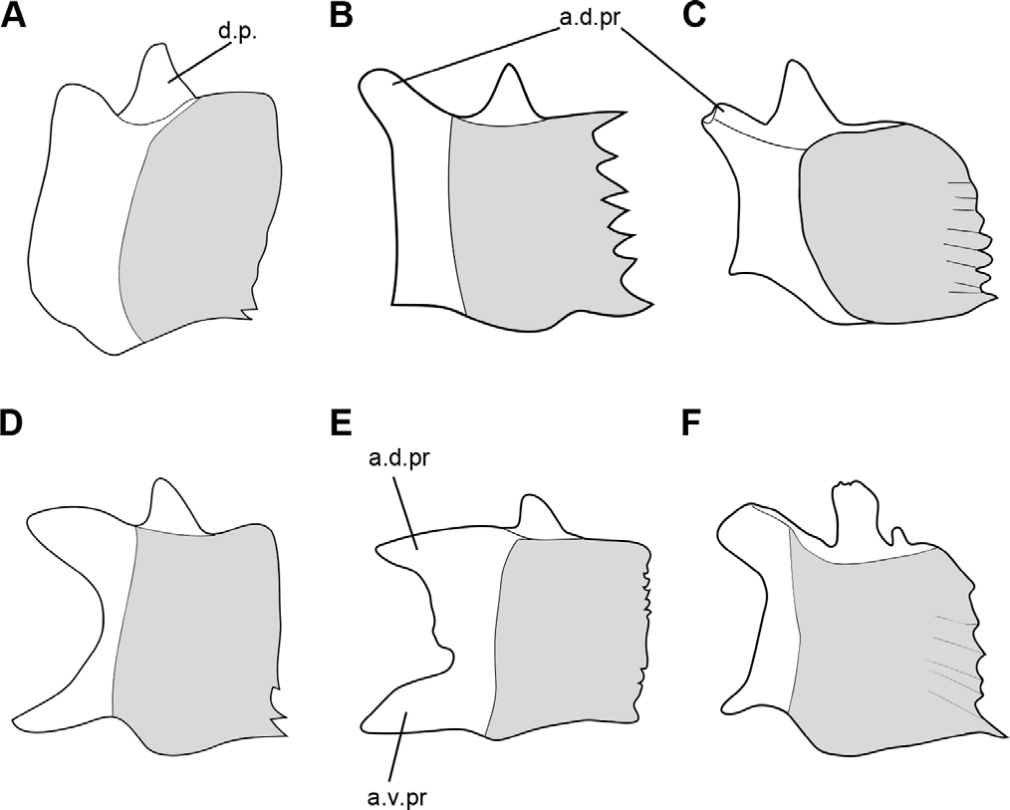
Different modes of scale articulation in ginglymodians. **A**, †*Sangiorgioichthys sui* GMPKU-P-1642. **B**, †*Semionotus bergeri* (NMC 15128a). **C**, †*Paralepidotus ornatus* (BSPG 2003-XXIX-218). **D**, †*Lepidotes minor* (NHMUK PV P8047). **E**, †*Lepidotes mantelli* (NHMUK PV 2397 and 4916). **F**, †*Araripelepidotes temnurus* (MNHN BCE-334). Abbreviations: a.d.pr, anterior dorsal process; a.v.pr, anterior ventral process; d.p, dorsal peg.

Normally in actinopterygians most of the scales of the body are articulated through the so called peg-and-socket articulation consisting in a dorsal spine-like peg protruding from the dorsal border of the scale (Fig. 15), which fits in a conical socket excavated in the medial surface of the scale. In some “semionotiforms” the scales have only very reduced pegs and sockets or this articulating structure is completely absent.

Character 86. Longitudinal articulation of the scales of the body.

1. Absent.
2. Single.
3. Double.

The peg-and-socket articulation explained above function in dorso-ventral direction and is there is an anterior area, without processes, which is overlapped by the adjacent scale (Fig. 15A; e.g. †*Sangiorgioichthys*). Additionally, the scales in many “semionotiforms” (e.g. †*Lepidotes gigas*, †*L*. *maximus*, †*L*. *laevis*, †*L*. *minor*, †*Tlayuamichin*) a rostro-caudal or longitudinal articulation consisting of two anterior processes, which protrude from the anteroventral and anterodorsal corners of the scale in anterior to anterodorsal directions (state 2; Fig. 15D–F). These processes can be as strong or stronger than the peg for the dorso-ventral articulation. In several “semionotiforms” (e.g. †*Semionotus*, †*Paralepidotus*, †*Pliodetes*), in the gars and in some out-group taxa the ventral anterior process is poorly developed and there is a strong dorsal anterior process (state 1; Fig. 15B–C).

Character 87. Posttemporal penetration by lateral line canal ([13]: character 91).

1. Present.
2. Absent.

Character 88. Supraorbital sensory canal in parietal (modified from [76]).

1. Supraorbital canal penetrates parietals at the central portion of these bones.
2. Supraorbital canal running almost on the lateral rim of the parietals.
3. Supraorbital canal does not penetrate the parietals.

The supraorbital sensory canal does not join the infraorbital canal in basal actinopterygians and penetrates the parietals at their central portion. Although a connection between the supraorbital and infraorbital sensory canals is possibly present in the posterolateral portion of the frontals, the canal runs further backwards penetrating the central portion of the parietals in most “semionotiforms” (state 0). In the gars and macrosemiids, the supraorbital sensory canal joins the infraorbital canal in the frontal or the dermopterotic and ends at this junction (state 2). A probably intermediate condition is observed in some taxa like †*Lepidotes gigas* or †*Scheenstia*, in which the supraorbital canal apparently joins the infraorbital canal at the posterolateral corner of the frontal, but it goes on backwards through the lateral rim of the parietals [37].

Character 89. Orbital canal.

1. Absent.
2. Present.

López-Arbarello & Sferco ([37]: 203, fig. 8) described for the first time and named the orbital sensory canal in †*Scheenstia zappi*. This sensory canal runs along the dorsal border of the orbit through one or more supraorbital bones and is present in other “semionotiforms” like †*Tlayuamichin* [32] and †*Macrosemimimus*, and also in *Lepisosteus* and *Atractosteus*.

Character 90. Deep groove housing the middle pit line in dermopterotic and parietal.

1. Absent.
2. Present.

In †*Scheenstia* ([37]: fig. 6) and other “semionotiforms” the pits representing the middle pit-line are aligned into a groove well excavated in the dermopterotic and the parietal.

### Description of the Results of the Cladistics Analysis

**Figure 16:**
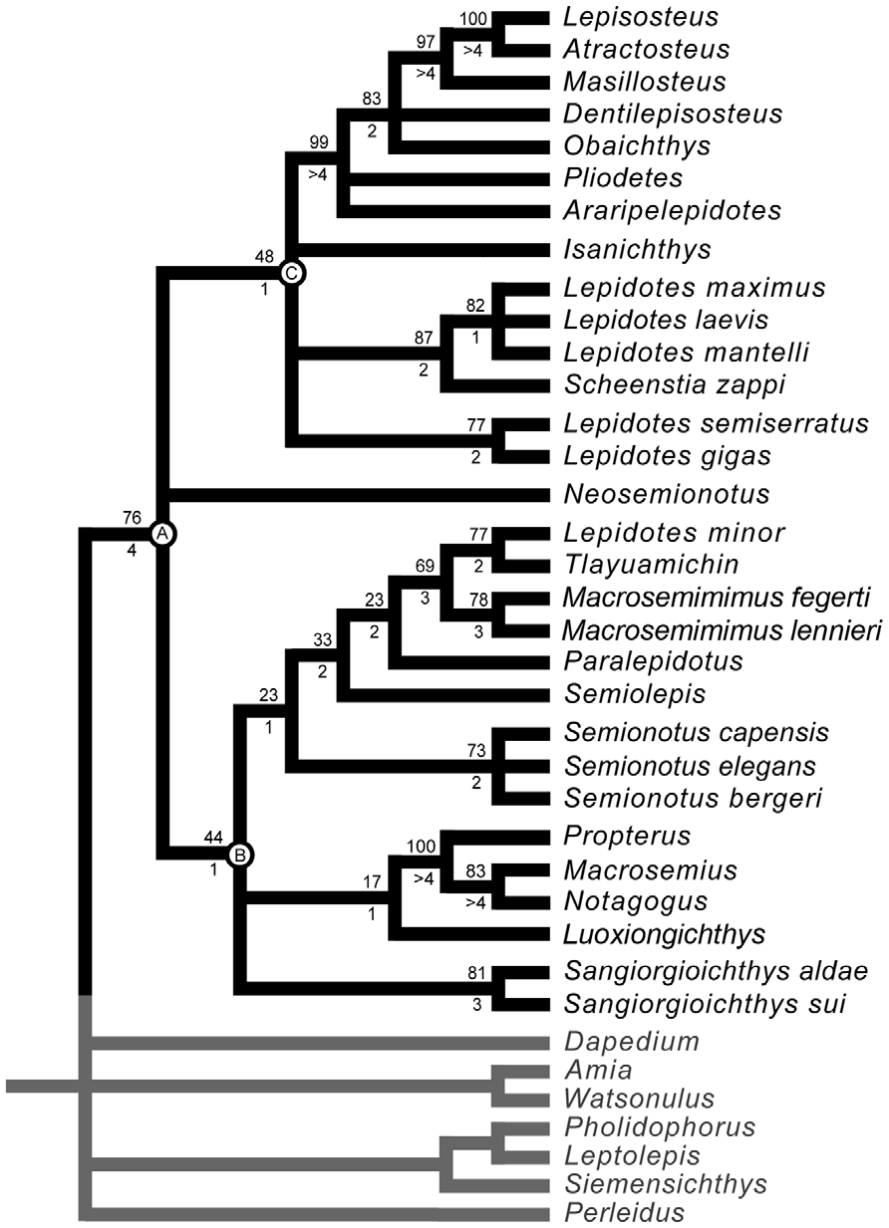
Strict consensus of 69 most parsimonious trees (92 characters, 39 taxa). Tree length  = 327; consistency index (CI)  = 0.3547; homoplasy index (HI)  = 0.6453; retention index (RI)  = 0.6608; rescaled consistency index (RC)  = 0.2344. Bootstrap and Bremer values are given above and below the branches leading to each node, respectively.

The “new technology” and heuristic searches in TNT and PAUP* produced equivalent results. The heuristic search in PAUP* produced 88 most parsimonious trees (MPT) of 272 steps (CI  = 0.4191; RI  = 0.7304; HI  = 0.5809; RC  = 0.3061). The Strict Consensus Tree (SCT) of these MPT is identical in both PAUP* and TNT and is represented in Figure 16, in which Bootstrap and Bremer values are given above and below the branches leading to each of the well-supported nodes, respectively. A detailed list of synapomorphies is provided in Table S1.

Although †*Dapedium* was not defined as an outgroup, it joined the polytomy formed by the ingroup and the other out-group taxa at the base of the tree. Therefore, †*Dapedium* is not more closely related to “semionotiforms” than to halecomorphs or teleosts in this analysis.

In the SCT the ingroup form a well-defined monophyletic group with seven unambiguous and 10 ambiguous synapomorphies, Bremer value of 4 and Bootstrap of 76. Except for †*Neosemionotus*, all the other ingroup taxa split in two major clades indicated at nodes A and B in the Figure 16.

Four unambiguous and six ambiguous synapomorphies define the clade at Node A, which is supported with decay index of 1 and Bootstrap value of 44. Three monophyletic groups form a polytomy at the base of this clade. †*Lepidotes minor* Agassiz, 1833 [58], from the British Purbeck is recovered as the sister group of †*Tlayuamichin itztli* López-Arbarello & Alvarado-Ortega, 2011 [32], from the Early Cretaceous of Mexico. This relationship has bootstrap value of 77, decay index of 2, and two unambiguous and one ambiguous synapomorphies. The monophyly of the new genus from Europe, which is described in [70] including the new species †*Macrosemimimus fegerti* and †*Macrosemimimus lennieri* Sauvage, 1893 [107], is confirmed based on four unambiguous synapomorphies and Bremer and Bootstrap values of 3 and 78, respectively. †*Macrosemimimus* is further recovered as the sister group of the clade (†*Tlayuamichin*, †*Lepidotes minor*) with five unambiguous and one ambiguous synapomorphies, Bremer of 3 and Bootstrap of 69. †*Paralepidotus* and †*Semiolepis* are stem taxa to this latter clade. A monophyletic †*Semionotus* including the three studied species †*S*. *bergeri*, †*S*. *capensis* and †*S*. *elegans* is defined with four unambiguous and two ambiguous synapomorphies, Bremer support of 2 and Bootstrap value of 73. In the SCT †*Semionotus* is the sister group of the clade (†*Semiolepis* (†*Paralepidotus* (†*Macrosemimimus* (†*Lepidotes minor*, †*Tlayuamichin*)))), but this relationship, although supported with four unambiguous and one ambiguous synapomorphies, has very low support with Bremer of 1 and Bootstrap of 23.

The monophyly of the family †Macrosemiidae is very strongly supported with decay index higher than 4, bootstrap value of 100, and 10 unambiguous and three ambiguous synapomorphies. The new Chinese taxon †*Luoxiongichthys* is the sister group of the †Macrosemiidae in the SCT, but with very low Bremer (1) and Bootstrap (17) values.

The monophyly of the Middle Triassic Chinese genus †*Sangiorgioichthys* with two species is recovered with four unambiguous and three ambiguous synapomorphies and Bremer and Bootstrap values of 3 and 81, respectively.

**Figure 17:**
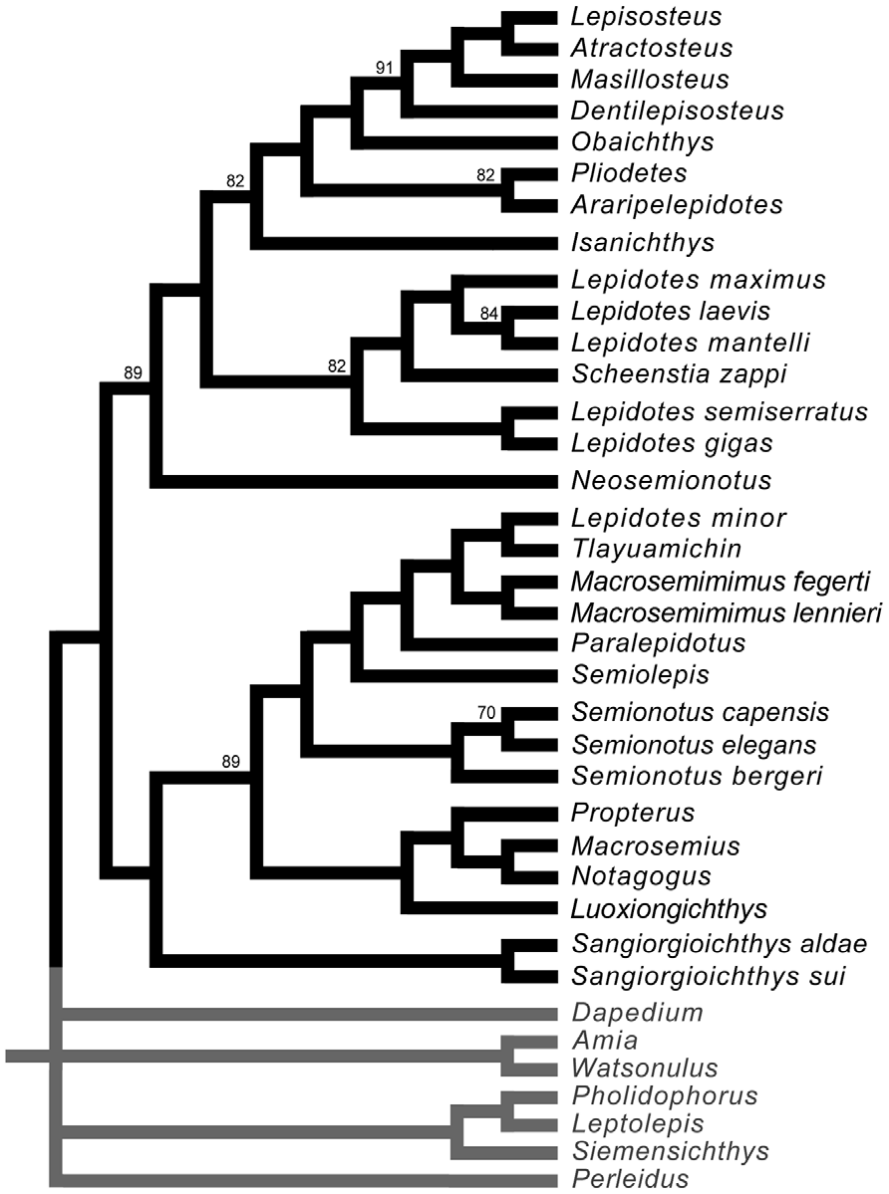
Majority rule consensus of 69 most parsimonious trees (92 characters, 39 taxa). Tree length  = 327; consistency index (CI)  = 0.3547; homoplasy index (HI)  = 0.6453; retention index (RI)  = 0.6608; rescaled consistency index (RC)  = 0.2344. The numbers above the branches indicate the percentage of MPTs containing the corresponding node.

The other main clade at node B represents a lineage leading to the gars and is resolved based on four unambiguous and eight ambiguous synapomorphies, Bremer of 1 and Bootstrap of 48. Three monophyletic groups and †*Isanichthys* form a polytomy at the base of this major clade. Among these groups, the monophyly of the Early Jurassic †*Lepidotes* is well supported with Bremer and Bootstrap values of 2 and 77, respectively, and four unambiguous and two ambiguous synapomorphies. The three strongly tritoral species previously referred to †*Lepidotes*, i.e. †*L*. *maximus*, †*L*. *laevis*, and †*L*. *mantelli* form a monophyletic group supported with one unambiguous and seven ambiguous synapomorphies, Bremer of 1 and Bootstrap of 82, which is more closely related to the genus †*Scheenstia* than to †*Lepidotes*. The sister group relationship between †*Scheenstia* and the three strongly tritoral species has decay index of 2, Bootstrap value of 87 and six unambiguous and three ambiguous synapomorphies. A sister group relationship between †*Lepidotes* and (†*Scheenstia* (†*L*. *maximus*, †*L*. *laevis*, †*L*. *mantelli*)) is recovered in 82% of the MPTs (see majority-rule consensus tree in Fig. 17).

The close relationships between †*Pliodetes* and †*Araripelepidotes* and the gars is very strongly supported with Bremer value higher than 4, Bootstrap value 99, and 15 unambiguous and 12 ambiguous synapomorphies at node C in Figure 16. A sister-group relationship between †*Pliodetes* and †*Araripelepidotes* is recovered in 82% of the MPTs (Fig. 17). Above these two taxa, the monophyly of the Lepisosteiformes sensu Grande ([13]; i.e. Obaichthyidae and Lepisosteidae) is recovered with eight unambiguous and 14 ambiguous synapomorphies, decay index of 2 and Bootstrap value of 83. The family Obaichthyidae Grande, 2010 [13] is not monophyletic in the strict consensus tree or in the majority rule consensus (Figs. 16, 17), but the monophyly of the family Lepisosteidae is confirmed with bootstrap value of 97, decay index higher than 4 and nine unambiguous and seven ambiguous synapomorphies. In 82% of the MPTs †*Isanichthys* is the sister group of the clade formed by †*Pliodetes* and †*Araripelepidotes* and the gars.

**Figure 18:**
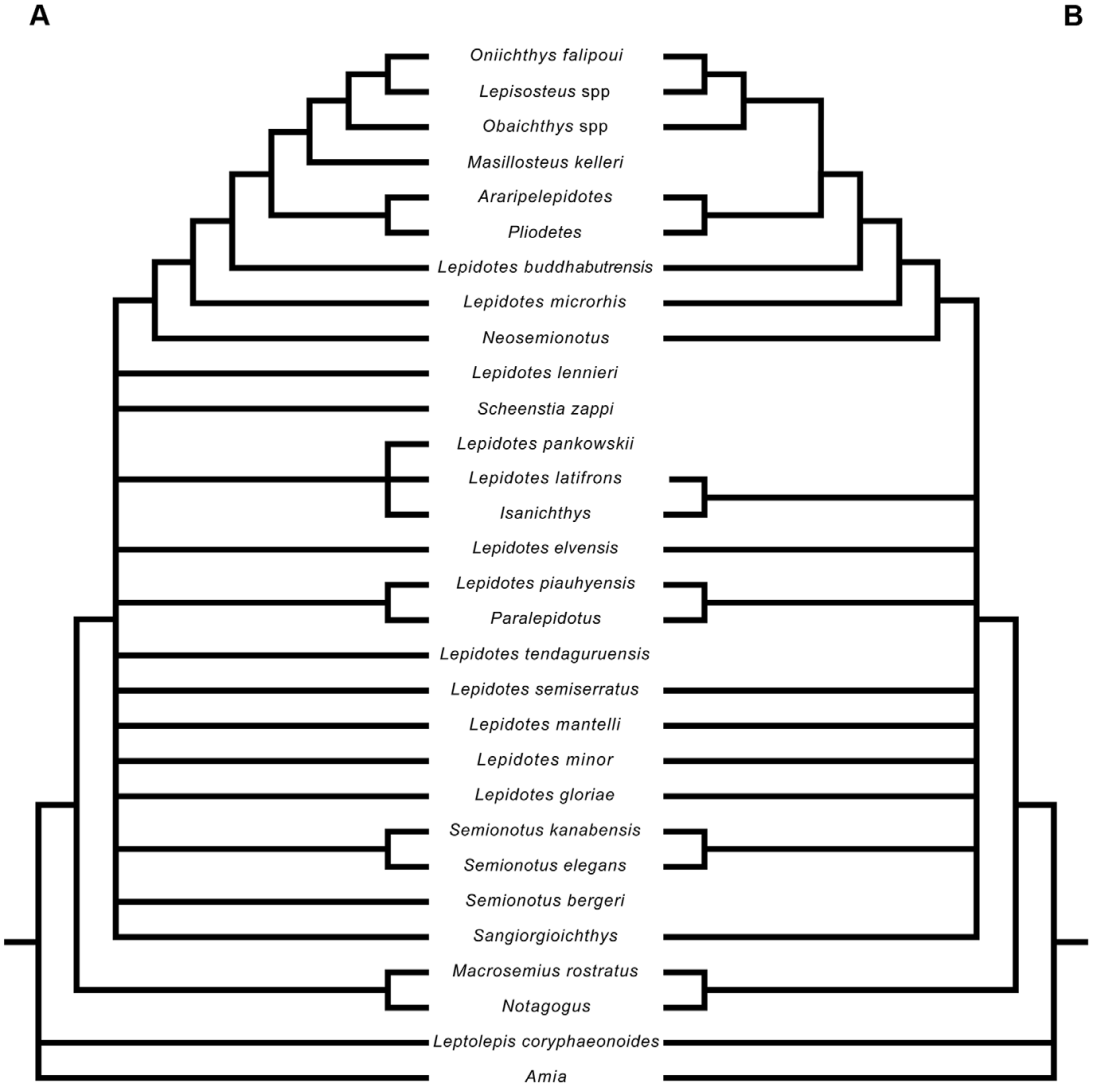
Results obtained after analyzing Cavin’s [**12**] data matrices excluding the artificial outgroup and, thus, using *Amia* and †*Leptolepis coryphaeonoides* as real out-group taxa. A , Strict consensus of 26 most parsimonious trees computed with 43 characters and 30 taxa. **B**, Strict consensus of 24 most parsimonious trees computed with 46 characters and 24 taxa.

The relationships of †*Neosemionotus* are not resolved in the strict consensus tree, but this taxon is the sister group of the clade defined at node B, leading to the gars, in 89% of the MPTs (Fig. 18).

## Discussion

### Comparison and Discussion of Previous Phylogenetic Hypotheses for “Semionotiform” Fishes

As a pioneer in the field, the cladistic analysis of Olsen & McCune [8] was the first study to demonstrate the sister group relationships between gars, “semionotids” and macrosemiids in a clade they named Semionotiformes. Subsequently, the monophyly of this clade was confirmed by each and every cladistic analysis, in which “semionotiform” fishes have been included (Figs. 1, 2) ([9]–[13], [108] and the present analysis). Similarly, the monophyly of gars was first demonstrated by Wiley [76] and subsequently confirmed by every cladistic analysis, with the most recent study [13] breaking with the idea of gars being more plesiomorphic than other neopterygians. Classified in the family Lepisosteidae in its own subclass Ginglymodi the gars were thought to be more plesiomorphic than the other neopterygians because they lack an interoperculum [75]. However, Grande [13] demonstrated that an interoperculum is present in the Cretaceous gars, which he classified in a separate family †Obaichthyidae. Reinforcing this evidence the cladistic analysis performed here confirms the presence of an interoperculum in the lineage leading to gars, which includes the fossil stem taxa †*Pliodetes* (without independent interoperculum) and †*Araripelepidotes* (with independent interoperculum). The present analysis also shows (like that of Cavin [12]) that the gars actually represent the crown group in one of two main clades at the base of the in-group (the clade defined at node B; see discussion of phylogenetic relationships below).

With the exception of the studies of Cavin & Suteethorn [10] and Cavin [12], the analysis presented here cannot be compared with previous cladistic analyses, apart from those patterns of higher-level relationships, because the data matrices are very different. The present analysis is the most comprehensive study of “semionotiform” fishes. The analysis is based on 30 in-group taxa, including almost all “semionotid” genera (†*Araripelepidotes*, †*Lepidotes*, †*Paralepidotus*, †*Pliodetes*, †*Semionotus*, †*Semiolepis*, and †*Tlayuamichin*), three macrosemiid genera (†*Macrosemius*, †*Propterus*, and †*Notagogus*), five lepisosteiform genera (*Lepisosteus*, *Atractosteus*, †*Masillosteus*, †*Obaichthys*, and †*Dentilepisosteus*; Lepisosteiformes sensu Grande [13]) and five “semionotiform” genera of uncertain relationships (†*Isanichthys*, †*Sangiorgioichthys*, †*Scheenstia*, †*Macrosemimimus* and †*Luoxiongichthys*). Cavin [12] also included numerous in-group taxa (28), representing almost all genera of gars, macrosemiids and “semionotids”, but the various analyses performed by this author are based on only 42 and 45 informative characters, respectively (vs. 90 informative characters included in this analysis) and have several problems that will be discussed in detail below. Finally, except for the analysis of lepisosteids and their fossil relatives by Grande [13], this is also the first cladistic analysis of “semionotiform” interrelationships using real out-group taxa instead of hypothetical ancestors [8], [10], [12].

#### Phylogenetic analyses of Cavin [12]

Although based on less than half the number of characters, the cladistic analyses presented by Cavin [12] have 20 (out of 28) in-group taxa in common with the analysis presented herein. However, the relationships proposed by Cavin for several of these taxa are very different and, thus, deserve detailed discussion.

Before discussing the differences between both studies it is worth noting that Cavin [12] already shows that the Cretaceous †*Neosemionotus*, †*Araripelepidotes* and †*Pliodetes* are stem taxa on the lineage towards Lepisosteidae and, thus, more closely related to the gars than to †*Lepidotes* or †*Semionotus* (Fig. 2). Cavin also recovered the basal position of the Middle Triassic †*Sangiorgioichthys* among “semionotiforms”, macrosemiids and gars, but apart from these agreements the phylogenetic relationships proposed for the other taxa in common with the current study are controversial.

According to Cavin [12] the macrosemiids are the sister group to all “semionotiforms” and gars, which contrasts with the more derived position, well nested within the main clade defined at Node A in my analysis (compare Figs. 2, 16). One of the three analyses performed by Cavin ([12]: fig. 2), which he chose for the discussion of relationships, also produced two main clades representing the taxa more closely related to †*Semionotus* than to gars on the one side (Node A in my analysis; unnamed in Cavin’s analysis) and the lepisosteid lineage on the other side (my Node B; Cavin’s fig. 2 node D). However, in Cavin’s hypothesis †*Isanichthys* and the species of †*Lepidotes* are more closely related to †*Semionotus* than to the gars, whereas my analysis produced the opposite results. Also, in my analysis the genus †*Lepidotes* is monophyletic, though restricted to the Early Jurassic species †*L*. *gigas* and †*L*. *semiserratus*. In Cavin’s analysis †*Lepidotes* is not monophyletic and †*L*. *gigas* (Cavin’s †*L*. *elvensis* in part) and †*L*. *semiserratus* are not sister groups. The specimens BMNH P.32421 and 14539 examined by Cavin and scored under the name of †*Lepidotes elvensis* come from the German Posidonia Shale and they correspond to the type species of the genus, †*Lepidotes gigas*, which is currently synonymized with †*L. elvensis*. However, it will be shown below that, although both species would score identical in Cavin’s data matrix, the French and German species are distinct in several features (see comments on the taxonomy of †*Lepidotes* below).

Further major discrepancies concern the relationships of †*Isanichthys*, and †*Paralepidotus*. According to Cavin †*Isanichthys* is more closely related to †*Lepidotes* or †*Semionotus* than to the gars. In my analysis, †*Isanichthys* is more closely related to the gars than to †*Semionotus*, and although the relationships between †*Isanichthys*, †*Lepidotes* and the gars are not resolved in the strict consensus tree, †*Isanichthys* is more closely related to the gars than to †*Lepidotes* in 82% of the MPTs (Fig. 17).

In Cavin’s analysis †*Paralepidotus* is more closely related to the species of †*Lepidotes* and †*Isanichthys* than to †*Semionotus* or the macrosemiids, but the opposite relationships were produced by my analysis. In the latter, †*Paralepidotus* is placed in the major clade defined at Node A and including the species of †*Semionotus* and the macrosemiids, among other taxa, but not †*Lepidotes* or †*Isanichthys*. Moreover, †*Paralepidotus* is included in a monophyletic group, which is the sister group of the †*Semionotus* (Fig. 16).

Besides the different treatment of some characters (see comments in the discussion of characters above), I disagree with Cavin [12] in the scoring of a number of taxa. According to my own study of the same specimens and literature of †*Lepidotes lattifrons* Jain & Robinson, 1963 [105], from the Oxford Clay, I was not able to confirm the scoring of at least 10 out of 29 characters Cavin scored for this species. The main problem with this species is that it is represented by two completely disarticulated specimens and a third fish with articulated postcranium preserved in lateroventral view and almost completely disarticulated skull. Therefore, many of the characters scored for †*L. lattifrons* seem to be based on reconstructions or assumptions about how the complete articulated fish would have looked.

The case of †*Lepidotes tendaguruensis* Arratia & Schultze, 1999 [109], and †*L*. *minor*, which appear with different phylogenetic relationships in the analysis presented by Cavin ([12]: fig. 1a) is noteworthy. The material of †*L*. *tendaguruensis* was first identified as †*L*. *minor* [110] and I will explain in the following section (first level beta-taxonomy) that, based on the available material, the two species are almost indistinguishable. Most of the features proposed by Arratia & Schultze [109] as diagnostic for †*L*. *tendaguruensis* are also present in †*L*. *minor*, and only after thorough analysis and comparison was I able to confirm the validity of the former species on the basis of two characters (see below). Cavin ([12]: supplementary material) did not examine the specimens of †*L*. *tendaguruensis* first hand, and, based on [109], he scored four characters with different states for †*L*. *tendaguruensis* and †*L*. *minor*, his characters 11, 20, 27, and 42 (first cladistic analysis). Thus, based on the evidence available to him, he scored the frontals as being narrower anteriorly than posteriorly in †*L*. *tendaguruensis* (ch. 25(1)), but as broad anteriorly as posteriorly in †*L*. *minor* (ch. 25(0)). However, according to my own observations, the frontals in the neotype of †*L*. *minor* (BGS.GSM 27975), as well as in the referred specimens, in which the frontals are well preserved (MB f. 1618, NHMUK PV P. 1118, 8047, 36080; see also [95]: pl. V figs. 6, 8) are narrower anteriorly than posteriorly. Cavin [12] scored the orbital ring as open (ch. 29(0)) in †*L*. *tendaguruensis* and closed (ch. 29(1)) in †*L*. *minor*. However, the condition of the orbit in most specimens of †*L*. *minor* is difficult to asses and the contact between the most anterior supraorbital and infraorbital bones in some specimens of †*L*. *minor* is an artefact of preservation: the infraorbital series is rotated and displaced against the anterior portion of the frontal in the left side of NHMUK PV P. 1118, the skull is preserved in dorsolateral view in NHMUK PV P. 36080 (see [95]: pl. V figs. 6 and 7 respectively) and the frontal is bent and preserved in dorsal view while the rest of the skull is preserved in lateral view in NHMUK PV P. 8047. Reconstructing the condition in the neotype and based on the specimen figured by Woodward ([95]: pl. V fig. 8), which is preserved in left lateral view with little displacement of the frontal and supraorbitals, the orbit of †*L*. *minor* is open anteriorly as it is the case in †*L*. *tendaguruensis*.

Cavin’s character 27 refers to the presence of tritoral dentition, which he considered absent in †*L*. *tendaguruensis*, as reported by Arratia & Schultze [109]. However, it will be explained below that the condition is unknown in this species, because coronoid and pterygoid dentitions are not preserved in the known specimens of this species. Finally, although there are “dorsal ridge scales lacking a posterior spine” (Cavin’s ch. 42(0)) in †*L*. *tendaguruensis* according to Arratia & Schultze ([109]: p. 138), I was not able to verify this feature with certainty in the poorly preserved specimens representing this species (see [109]: figs. 2–3).

Other disagreements with character scorings of Cavin [12] will not be discussed in detail here because the main problem I find in his analyses is the use of an artificial hypothetical outgroup. Cavin [12] does not explain how he inferred the hypothetical ancestor, and applying the outgroup algorithm of Maddison et al. [111] it is not possible to recover the same ancestral states that he used in his analyses. The use of hypothetical ancestors has fallen into disuse and Bryant ([112]: p. 345) has shown that “the use of a priori hypothetical ancestors as additional terminal taxa is either potentially problematic (outgroup comparison), or invalid (ontogenetic and paleontological methods)”. Therefore, and since Cavin [12] also included two real out-group taxa in his data matrices (*Amia calva* and †*Leptolepis coryphaeonides*), I re-analysed his data matrices excluding the hypothetical outgroup and the results are shown in Figure 18. Thus, using real outgroups for the data matrix presented by Cavin [12] leads to significantly lower resolution of the tree, and most of the inconsistencies between his analyses and the one presented here are no longer present in the strict consensus tree.

### First Level Beta-taxonomy

Based on the results of the present cladistic analysis described above, the following taxonomic changes are proposed (Table 1). The generic diagnoses are based on unambiguous synapomorphies only but, additionally, distinctive combinations of features are provided to facilitate identifications.

**Table 1:** Generic changes of some “semionotiform” taxa, based on the results of this phylogenetic study.

Original name	New combination
†*Lepidotes mantelli* Agassiz, 1833 [58]	†*Scheenstia mantelli*
†*Lepidotes laevis* Agassiz, 1837 [58]	†*Scheenstia laevis*
†*Lepidotes maximus* Wagner, 1863 [119]	†*Scheenstia maximus*
†*Lepidotes decoratus* Wagner, 1863 [119]	†*Scheenstia decoratus*
†*Lepidotes degenhardti* Branco, 1885 [120]	†*Scheenstia degenhardti*
†*Lepidotes hauchecornei* Branco, 1885 [120]	†*Scheenstia hauchecornei*
†*Lepidotes minor* Agassiz, 1833 [58]	†*Callipurbeckia minor*
†*Lepidotes tendaguruensis* Arratia & Schultze, 1999 [109]	†*Callipurbeckia tendaguruensis*
†*Lepidotes notopterus* Agassiz, 1833 [58]	†*Callipurbeckia notopterus*

### Genus †LEPIDOTES Agassiz, 1832 [25]

#### Type species

**Figure 19:**
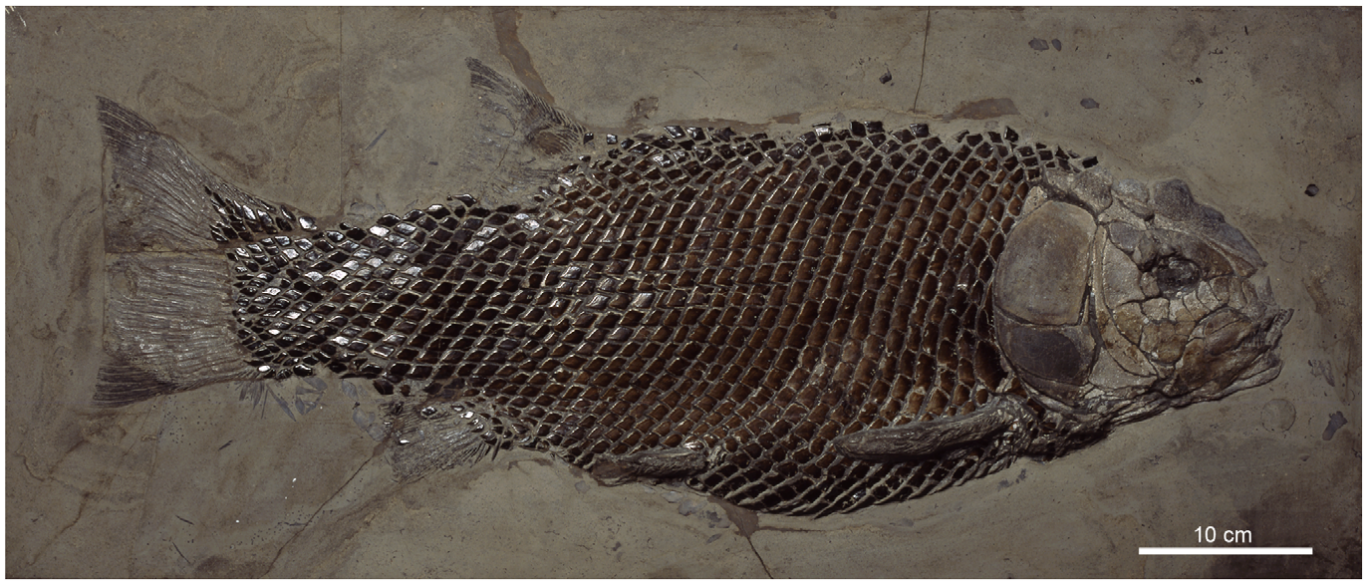
†*Lepidotes gigas* Agassiz, 1832 [**25**] **. BSPG 1940-I-8, SL  = 60.5 cm, from the area of Holzmaden, Germany.**

†*Lepidotes gigas* Agassiz, 1832 [25] from the Late Toarcian of Holzmaden (Germany) (Figs. 8, 19).

#### Referred species

†*Lepidotes elvensis* (Blainville, 1818) [69] from the Toarcian of La Caine, Elbes, (France), †*Lepidotes semiserratus* Agassiz, 1836 [58] from the Toarcian of Whitby (England), †*Lepidotes bülowianus* Jaekel, 1929 from the Toarcian of Dobbertin, Mecklenburg-Vorpommen (Germany).

#### Diagnosis

First anterior infraorbital bone deeper than more posterior anterior infraorbitals; approximately squared-shaped infraorbital bones forming the posterior border of the orbit; peg-and-socket articulation reduced or absent; middle pit line contained in a groove excavated in dermopteroticum and parietal.

Additionally, the following combination of features is distinctive of †*Lepidotes*: large fusiform fishes with body depth c. 35% of the standard length (SL) and head length c. 30% SL; pelvic, dorsal and anal fins placed in the posterior half of the body, the pelvic fins inserting at c. 55% SL, dorsal fin inserting at c. 65% SL, and anal fin inserting at c. 75% SL; the presence of a single pair of extrascapular bones (ACCTRAN); numerous suborbital bones of variable size and shape, arranged in a series, which extends ventral to the orbit covering the quadrate laterally; thick ganoid scales with strongly developed longitudinal articulation through large dorsal and ventral anterior processes.

#### Remarks

†*Lepidotes* has been one of the largest “wastebasket” genera of Mesozoic actinopterygians and most of the species previously referred to this genus either represent independent taxa or should be regarded as *nomina dubia*.

**Figure 20:**
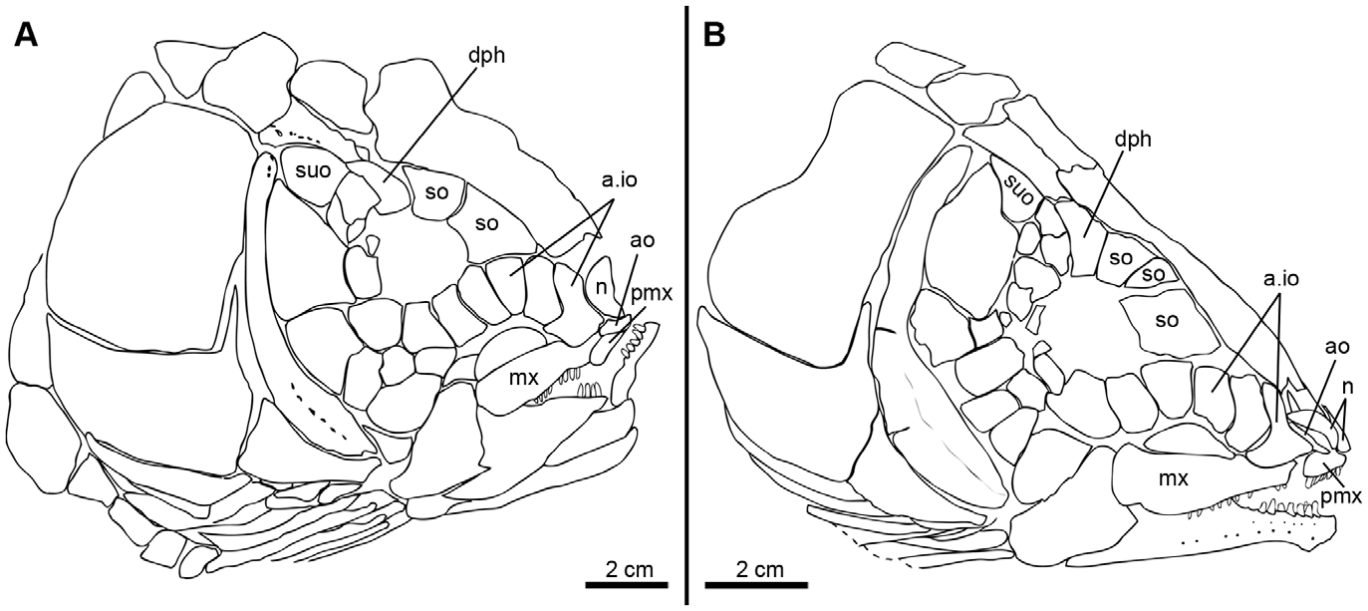
Distinction between: A, †*Lepidotes gigas* (BSPG 1940-I-8) and B, †*Lepidotes elvensis* (MNHN JRE-250). Abbreviations: a.io, anterior infraorbital; ao, antorbital; dph, dermosphenoticum; mx, maxilla; n, nasal; pmx, premaxilla; so, supraorbital; suo, suborbital.

†*Lepidotes semiserratus* Agassiz, 1836 [58] from England and †*L. bülowianus* Jaekel, 1929 [114] from Germany differ from the previously described species in having strongly serrated scales and tritoral dentition [115], [116]. The dermal bones in the skull of †*L. bülowianus* including all circumborbital and suborbital bones are furthermore very densely ornamented with ganoine tubercles, which are absent in †*L. semiserratus*. The precise limits and relationships between these four coeval species of †*Lepidotes* need further study. A thorough revision of all the available material has not been done so far and it is not yet possible to assert if these †*Lepidotes* species mirror the endemism shown by plesiosaurs, ichthyosaurs and marine crocodiles within in the lower Toarcian seas of Western Europe. However, based on the published material, they seem to follow the three or four marine reptile zones proposed by Godefroit [117] and Maisch & Ansorge [118].

The genus †*Lepidotes* Agassiz, 1832 [25] was erected for two fish specimens from the Posidonienschiefer (Toarcian) at Ohmden near Boll in Germany. Some years later Agassiz found this fish indistinguishable from a specimen from the Lias (Toarcian) of La Caine in France, which had already been named †*Cyprinus elvensis* Blainville, 1818 [69] and, thus, he put the two species in synonymy, but kept the name †*Lepidotes gigas* for this taxon [58]. Later Quenstedt [113] proposed the combination †*Lepidotes elvensis* for the German and French nominal species. Although the three specimens of the French species at the Muséum National d’Histoire Naturelle in Paris (the holotype MNHN JRE-545 and two other specimens MNHN JRE-250, 254) are more poorly preserved than the German material, a few anatomical differences support the validity of two different species (Fig. 20). Accordingly, †*L*. *gigas* Agassiz, 1832 [32] and †*L*. *elvensis* (Blainville, 1818) [69] differ in the general shape of the skull, the number of supraorbital bones (2 vs. 3 respectively), the relative size of the first, most dorsal suborbital bone, which is relatively trapezoidal and largest in †*L*. *gigas* while triangular, narrowing posterodorsally in †*L*. *elvensis*. Additionally, the maxilla is somewhat larger and the snout a little longer in †*L*. *elvensis* than in †*L*. *gigas*. Though the number of anterior infraorbital bones is the same, the frontal and most anterior supraorbital are slightly differently arranged in these species, so that the frontal extends over three anterior infraorbitals in †*L*. *elvensis*, but over two anterior infraorbitals in †*L*. *gigas*. Although †*L*. *elvensis* has been erroneously cited as the type species of the genus (e.g. [16], [18], [95], [109]), according to the International Code of Zoological Nomenclature (Article 67.2) only originally included nominal species are eligible as type species of a genus. Therefore, since Agassiz [25] only included †*L*. *gigas* when creating the nominal genus †*Lepidotes*, this species, and not †*L*. *elvensis*, is the type species of †*Lepidotes*.

### Genus †SCHEENSTIA López-Arbarello & Sferco, 2011 [37]

#### Type species

†*Scheenstia zappi* López-Arbarello & Sferco, 2011 [37] from the Kimmeridgian of Schamhaupten (Germany).

#### Referred species

†*Lepidotes mantelli* Agassiz, 1833 [58] from the Wealden of Sussex and Isle of Wight and the Upper Purbeck Beds of Sussex (England), †*Lepidotes laevis* Agassiz, 1837 [58] from the Late Kimmeridgian of Cerin (France), †*Lepidotes maximus* Wagner, 1863 [119] from the Tithonian Solnhofen limestones at Kelheim, Eichstätt and Langenaltheim (Germany), †*Lepidotes decoratus* Wagner, 1863 [119] from the Tithonian Solnhofen limestones at Solnhofen (Germany), and †*Lepidotes degenhardti* Branco, 1885 [120], and †*Lepidotes hauchecornei* Branco, 1885 [120] from the “Wealden” of Obernkirchen (Germany).

#### Diagnosis

Three or more pairs of extrascapular bones; in the series of suborbital bones that extend ventral to the orbit covering the quadrate laterally, the first and last suborbitals are the largest; dentition extremely tritoral; strong knob-like anteroventral process in posttemporal bone; orbital sensory canal present; middle pit line contained in a groove excavated in dermopteroticum and parietal.

Additionally, the following combination of features is distinctive of †*Scheenstia*: large fishes with fusiform bodies with body depth c. 40–45% of the standard length (SL) and head length c. 30% SL; pelvic, dorsal and anal fins placed in the posterior half of the body, the pelvic fins inserting at c. 50–53%, dorsal fin inserting at c. 65–70% SL, and anal fin inserting at c. 75–78% SL; infraorbitals at the posterior border of the orbit longer than deep; maxilla edentulous, very short and deep (ACCTRAN), ends at the level or before the anterior border of the coronoid process; thick ganoid scales with vertical peg-and-socket articulation variably developed and very well developed longitudinal articulation through large dorsal and ventral anterior processes.

#### Remarks

The close relationship between †*Scheenstia zappi* and †*Lepidotes* was already put forward by López-Arbarello & Sferco [37]. These authors also discussed the close resemblance between this fish and the large tritoral forms that have been referred to †*Lepidotes* (i.e. †*L*. *laevis*, †*L*. *mantelli*, †*L*. *maximus*), which are now transferred to †*Scheenstia* based on the derived characters shared by them and the type species of this genus, †*S*. *zappi*. †*Scheenstia mantelli* (Agassiz, 1833) [58] is certainly the best known among these tritoral fishes [95]. †*Scheenstia laevis* (Agassiz, 1837) [58] is best known from an excellently preserved though incomplete specimen described by Saint-Seine [121].

**Figure 21:**
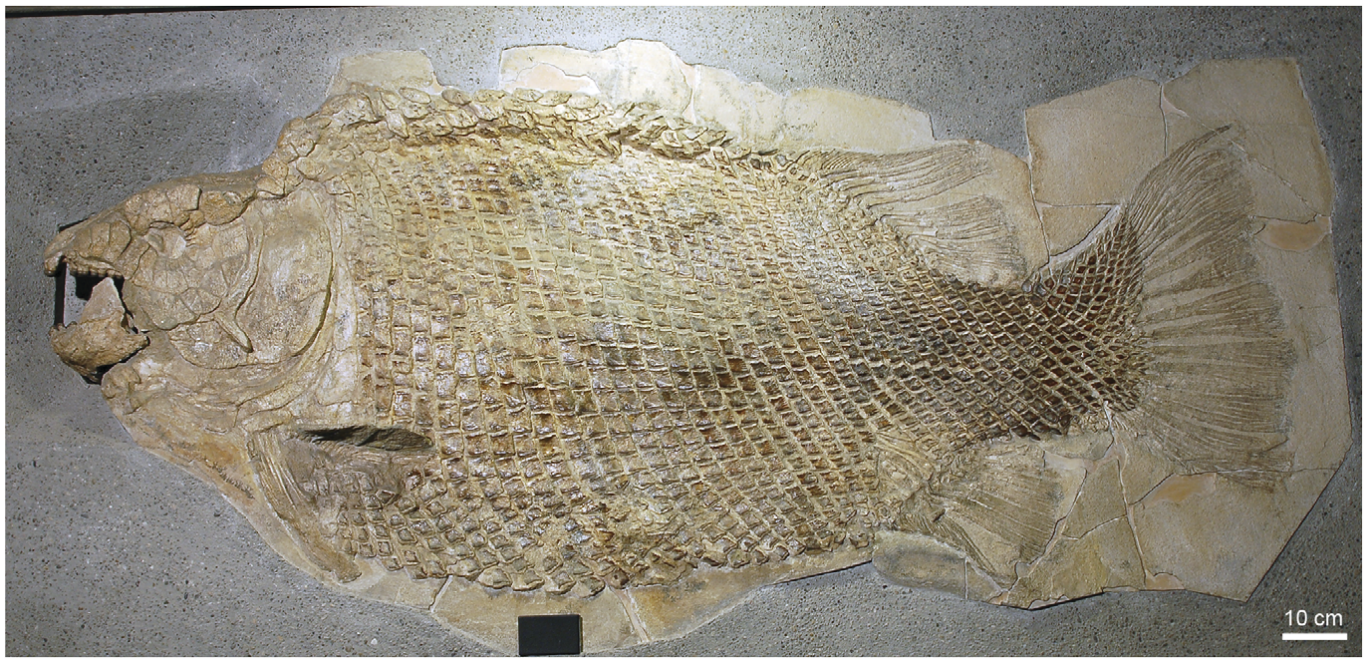
Neotype of †*Scheenstia maximus* (Wagner, 1863) [**119**] **. SMF P.2386, SL  = 168 cm, from Solnhofen limestones at Langenaltheim, Bavaria, Germany.**

Probably the largest and more impressive species in this genus is †*Scheenstia maximus* (Wagner, 1863) [119] (Fig. 21). The type material of this species was stored at the Bayerische Staatssammlung für Paläontologie und Geologie in Munich when Wagner ([119]: 19) described the species, but it is unfortunately lost. The material was most probably destroyed during the Second World War, as many other specimens in this collection, the house of which was severely bombed. The type material included two fragmentary specimens containing several articulated scales. Wagner described only one of them, consisting of a fragment (approximately 61 cm high x 37 cm long) of a large fish including several articulated scales mostly exposed in medial view, though at least some of them exhibited their lateral surface. Unfortunately, Wagner did not illustrate the specimen, which is neither figured nor described in any other publication. However, the characteristics described by Wagner for the scales in the type specimen, perfectly match the scales in the specimens SMF P.325 and SMF P.2386 of the Senckenberg Museum in Frankfurt, which has been studied by Jain [103]. According to Wagner the type specimens were found in the Solnhofen limestones of Kelheim, Solnhofen and Eichstätt. The two almost complete specimens in the Senckenberg Museum come from the Solnhofen limestones at Langenaltheim, which represents the same depositional centre as the locality of Solnhofen and are well correlated with the equivalent outcrops at Kelheim in the *Rueppellianus* Subzone, and with those of Eichstätt in the *Hybonotum* Zone (lower Tithonian; [122]). Therefore, the specimen SMF P.2386 (Fig. 21) is here designated neotype of †*Scheenstia maximus* (Wagner, 1863) [119] new combination, to provide objective evidence for this species and avoid confusion over its characteristics.

**Figure 22:**
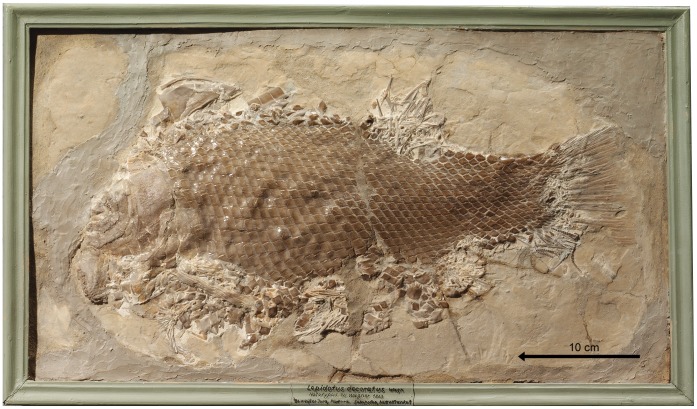
Holotype of †*Scheenstia decoratus* (Wagner, 1863) [**119**] **. BSPG AS-VI-3, estimated SL  =  c. 43 cm.**

A second species described by Wagner [119], †*Scheenstia decoratus* from Solnhofen (*Hybonotum* Zone, Solnhofen Formation; early Tithonian; [122]) is represented with a rather complete specimen only (Fig. 22). Although the skull is only partially preserved, the holotype is generally very similar to the recently described †*S*. *zappi* from Schamhaupten (Beckeri Zone, Rögling Formation; latest Kimmeridgian; [122]), but differs from this species in the ornamentation of the skull bones, which is made up of densely arranged broad tubercles and ridges that reach the free margin of the suborbital and infraorbital bones producing a crenulated border, very different from the much more sparsely and smaller tubercles with no ridges in †*S. zappi*, the lower jaw is notably more robust and the scales more strongly serrated in †*S*. *decoratus* than in †*S*. *zappi*. Due to the incomplete preservation it is not possible to take exact measurements in the holotype and so far only known specimen of †*S*. *decoratus*, but the body is somewhat more slender and the head was certainly smaller than the head of †*S. zappi*. Although none of the fins in †*S*. *decoratus* is complete enough to allow detailed comparison, further differences in the body are the total number of vertical rows of scales (38 vs. 37), the number of inverted rows of scales forming the body lobe of the tail (8 vs. 10). Two poorly known species from the German “Wealden”, †*S. degenhardti* Branco, 1885 [120] and †*S. hauchecornei* Branco, 1885 [120] are tentatively referred to †*Scheenstia*, thought they need detailed revision.

### Genus †CALLIPURBECKIA gen. nov

urn:lsid:zoobank.org:pub:BFFD7527-33BA-41D5-AF0F-CFD43625FDBE.

#### Etymology

From the Ancient Greek “calli-”, beautiful, and Purbeck, the current name of the area inhabited by the fish.

#### Type species

**Figure 23:**
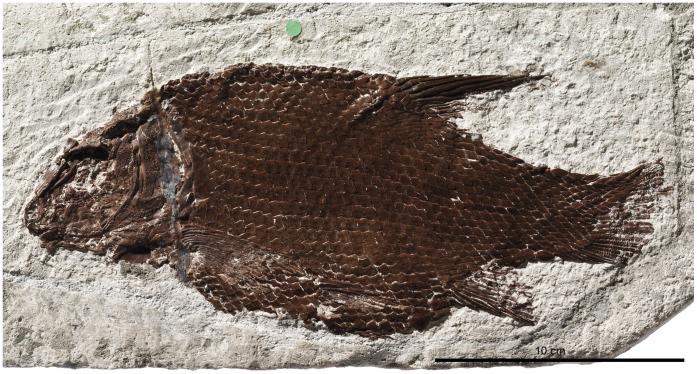
Lectotype of †*Callipurbeckia minor* (Agassiz, 1833) [**58**] **. GSM 27975, LS  = 23.5 cm, from the Middle Purbeck Beds at Swanage, Dorset (England).**

†*Lepidotes minor* Agassiz, 1833 [58] from the Middle Purbeck Beds at Swanage, Dorset (England) (Fig. 23).

#### Referred species

†*Lepidotes notopterus* Agassiz, 1833 [58] from the Solnhofen limestones in Germany (precise type locality unknown); †*Lepidotes tendaguruensis* Arratia & Schultze, 1999 [109] from the Tithonian of Tendaguru (Tanzania).

#### Diagnosis

The following combination of features is distinctive of †*Callipurbeckia*: medium size semionotiform fishes with fusiform bodies with body depth c. 45% of the standard length (SL) and head length c. 30% SL; pelvic, dorsal and anal fins placed in the posterior half of the body, the pelvic fins inserting at c. 50%, dorsal fin inserting at c. 65% SL, and anal fin inserting at c. 75% SL; skull bones ornamented with tubercles; single pair of extrascapular bones; two suborbital bones, a small oval dorsal suborbital and a much larger ventral suborbital filling most of the area between the infraorbitals and preoperculum; maxilla deep, forming a more or less circular plate; dentition moderately tritoral; conspicuous dorsal ridge of scales; ganoid scales with well developed vertical and longitudinal articulation with large dorsal peg and large dorsal and ventral anterior processes.

#### Remarks

Agassiz [58] coined the binomen †*Lepidotes minor* for a species commonly found in the Purbeck sequences at Swanage, which he represented with a specimen in the collection of the School of Mines in Paris ([58]: pl. 34). Since this original type has been lost, probably during the Second World War, McCune [123] designated the specimen BGS.GSM 27979 as the neotype. The species †*L. minor* was first revised by Woodward [95] who provided a complete description and excellent illustrations, including a complete drawing of the specimen later designated neotype (BGS.GSM 27979). In a taxonomic revision of the genus †*Semionotus* McCune [123] found †*L. minor* most similar to this genus and proposed the new combination †*Semionotus minor*. Although †*L. minor* certainly shares some similarities with the species of †*Semionotus*, according to the results of the cladistic analysis the Purbeck species cannot be referred to that genus or to †*Lepidotes* either. Instead, †*L. minor* is most closely related to †*Tlayuamichin itztli* López-Arbarello & Alvarado-Ortega, 2011 [32] from the Albian of Mexico. Although this sister-group relationship is as strong as the relationship shown by other species within a single genus, several apomorphic features of †*Tlayuamichin* (see diagnosis in [32]) in addition to the geographic and chronostratigraphic differences between the two species support the establishment of separate genera.

Among the species referred to this genus, the “semionotiforms” from the Upper Saurian Bed (Late Jurassic: Tithonian) in Tendaguru, Tanzania, representing the species †*Lepidotes tendaguruensis* Arratia & Schultze, 1999 [109], were originally referred to †*Lepidotes minor* [110] and after studying the material at the Museum für Naturkunde in Berlin, I find little evidence supporting different species. The few known specimens are very poorly preserved and it is not possible to corroborate several detailed anatomical features proposed in the diagnosis of †*C. tendaguruensis*. Since no detail structures like the distinct sockets are observable in the bone identified as the epiotic in †*C. tendaguruensis*, I am not sure if this element actually represents this bone. Nonetheless, even accepting this interpretation of the bone, a digitated posterior process is common to all “semionotiforms” for which the epiotic is known (see Cavin [12]: character 1). The series of supraorbitals is surely incompletely preserved in †*C. tendaguruensis* and at least one supraorbital is missing anteriorly (see [109]: figs. 4, 6–7). Therefore, there are certainly more than two supraorbital bones in these fishes and there are three supraorbitals in †*C. minor* (NHMUK PV P1118, P8047, P36080). The relative size and shape of the two suborbital bones, as well as the teeth on the premaxilla and dentary are basically the same in both nominal species.

A very peculiar feature reported in †*C. tendaguruensis* is the presence of two most anterior infraorbital bones horizontally placed, one dorsal to the other. According to my observations the two “anterior infraorbital bones or antorbitals, rectangular shaped and placed above each other” ([109]: 138) in MB. f.7048 rather represent two fragments of the most anterior anterior infraorbital, dorsal and ventral to the sensory canal, which is deeply excavated in the infraorbital bones of †*C. tendaguruensis* (see [109]: fig. 6). Accordingly, †*C. tendaguruensis* would have three and not four anterior infraorbitals, as is the case in the neotype of †*C. minor*. However, the anterior region of the skull of †*C. tendaguruensis* is not completely preserved in any of the known specimens and, thus, the exact number of anterior infraorbitals is unknown.

Arratia & Schultze ([109]: p. 145) described two rows of teeth on the dentary (their dentalosplenial) of †*C. tendaguruensis*, which would also represent a remarkable feature. The presence of two rows of teeth on the dentary, a lateral row of small pointed teeth plus an inner row of much more robust fangs is a unique feature of *Lepisosteus* and *Atractosteus* and unknown in “semionotiforms”, which have only one row of teeth on the dentary (see character 85 in this cladistic analysis and [13]: character 39). However, the only well preserved dentary of †*C. tendaguruensis* in the specimen MB. F.7043 has a single row of teeth (pers. obs.; see also [109]: figs. 5A and 10C). On the other hand, tritoral dentition is alleged to be absent in †*C. tendaguruensis*, but the tritoral teeth of †*C. minor*, as in many other semionotiforms, are not dentary teeth, but on the coronoid bones (NHMUK PV P.29399, 17329), and they were described in detail by Jain ([74]: 30). Therefore, since no coronoid bone is preserved in any of the specimens of †*C. tendaguruensis*, there is no evidence for the alleged absence of a tritoral dentition.

**Figure 24:**
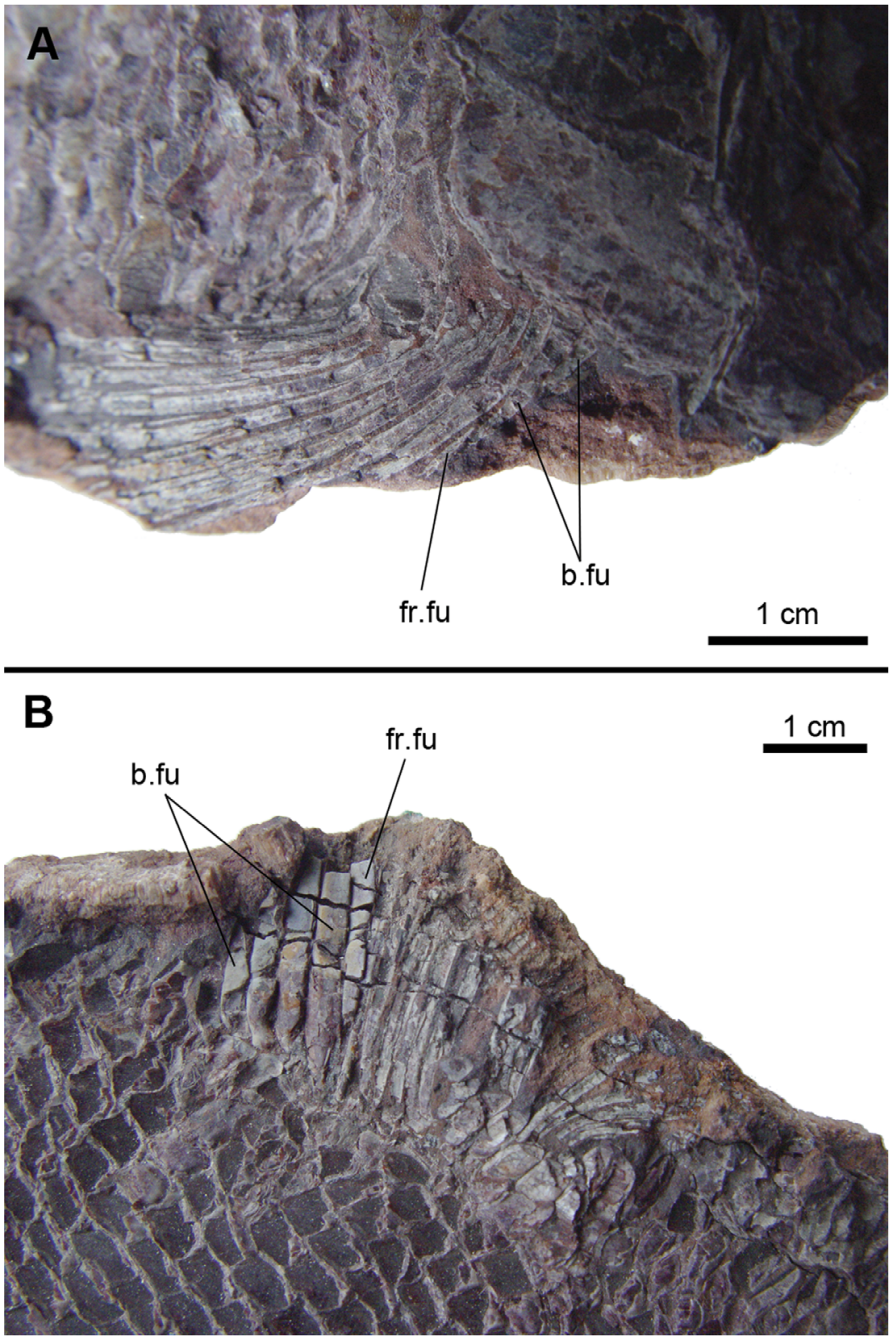
Fringing and basal fulcra in †*Callipurbeckia tendaguruensis* (Arratia & Schultze 1999) [**109**]. A, Pectoral fin in MBf 7040. B, Dorsal fin in MBf 7041. Abbreviations: b.fu, basal fulcra; fr.fu, fringing fulcrum.

As mentioned before, the specimens of †*C. tendaguruensis* are poorly preserved and I was not able to confirm the anterior membranous outgrowths of the hyomandibula described and illustrated by Arratia & Schultze ([109]: compare the photograph of the cast in fig. 6A with the interpretative drawing of same cast in fig. 7B). Similarly, the series of postcleithra and the dorsal ridge scales are incompletely preserved. On the other hand, the alluded absence of fringing fulcra in †*C. tendaguruensis* is erroneous. Fringing fulcra are present at least in the pectoral (MB. f.7040) and dorsal (MB. f.7041) fins of this species (Fig. 24).

Therefore, the only two features that distinguish †*C. tendaguruensis* from †*C*. *minor* are a comparatively short preoperculum that does not reach the dermopterotic and the ventroposterior expansion of the infraorbital bone placed at the posteroventral corner of the orbit, as noted by Arratia & Schultze [109]. The shape of the infraorbital bones is somewhat variable individually in all “semionotiforms” I have examined and one to one relationships of homology cannot be established for the individual bones in the infraorbital series (see the above discussion of characters). However, all infraorbital bones from the posteroventral to the anteroventral corner of the orbit reach the depth of their adjacent elements in the series in †*C. minor*, but not in †*C. tendaguruensis*, in which the infraorbital bone at the posteroventral corner of the orbit expands ventroposteriorly respect to the circumference drawn by the other infraorbital bones (compare [109]: fig. 7A with Fig. 24). Based on the two latter features, the species named by Arratia & Schultze is here confirmed as valid and based on its close resemblance with †*C. minor* it is referred to †*Callipurbeckia* gen. nov.

Although it needs thorough revision, another species of this genus is probably †”*Lepidotes*” *notopterus* Agassiz, 1833 [58]. According to Woodward [95] this species might occur in the Wealden Formation. However, the type specimens described by Agassiz came from the Solnhofen limestones. I have observed several unstudied specimens from the Solnhofen limestones, which are almost indistinguishable from †*C. minor* (e.g. MB. f.17878). I was not able to find any of the type specimens in the Natural History Museum and it is not clear whether the fish from the Wealden described and figured by Woodward actually represents this species or a different, still unnamed taxon (note the important chronostratigraphic difference between the Wealden and Solnhofen formations). Although strikingly similar, †*C. notopterus* apparently differs from †*C. minor* in some morphometric proportions and a few meristic and osteological features (pers. obs.).

### Discussion of Phylogenetic Relationships and Suprageneric Taxonomy

**Figure 25:**
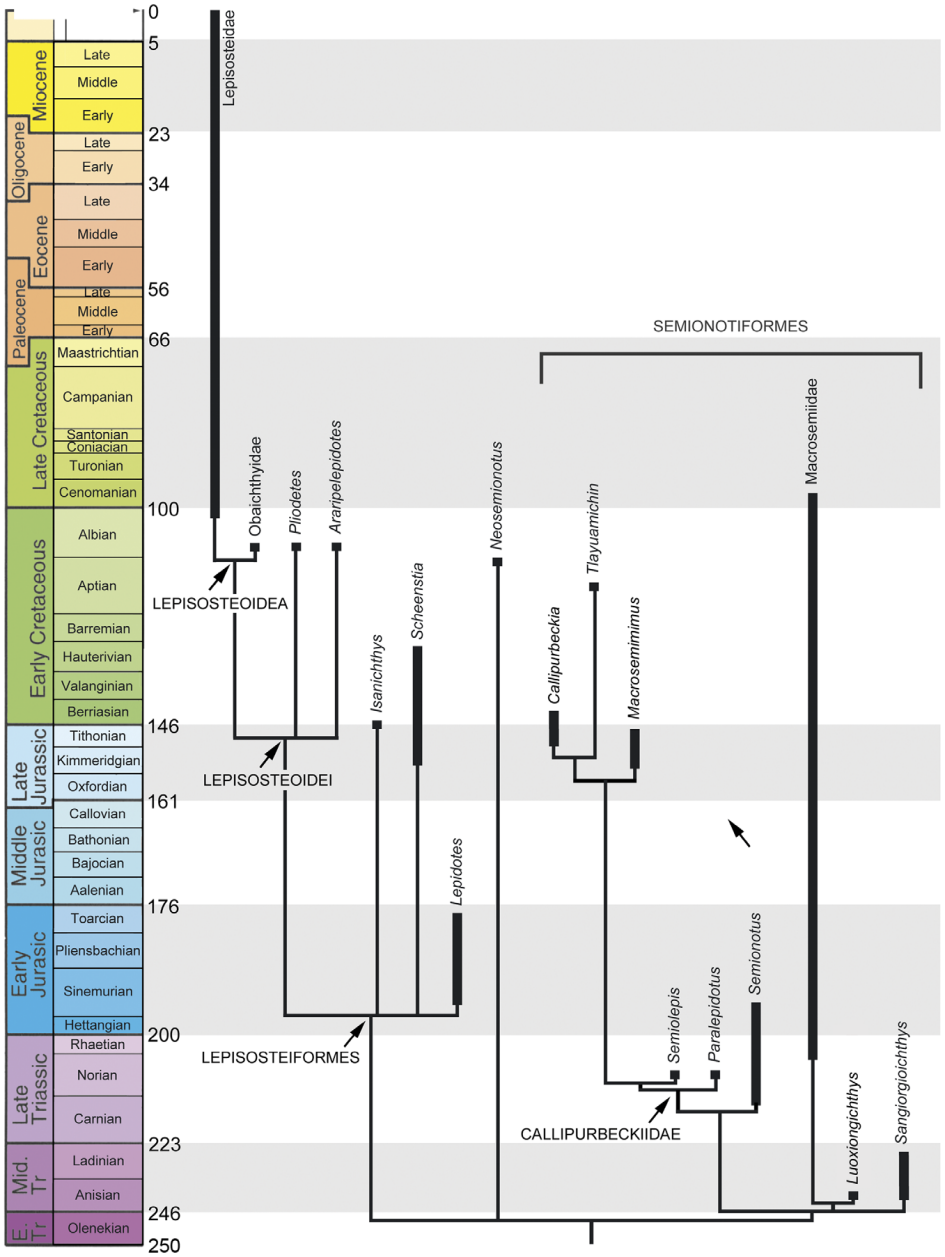
Callibrated phylogenetic hypothesis of ginglymodians interrelationships based on a simplyfied version of the strict consensus tree shown in **Figure 17**.

In addition to the taxonomic changes at the generic level that were explained in the previous section, the suprageneric classification of the studied taxa is here revised based on the phylogenetic relationships recovered in the strict consensus tree (Fig. 16), which is presented as a simplified cladogram in Figure 25. The following diagnoses proposed for the taxa above the generic rank are based on unambiguous and ambiguous synapomorphies. The unambiguous synapomorphies are indicated with an asterisk “*” and the ambiguous synapomorphies with “(ACCTRAN)” or “(DELTRAN)” depending on the optimization method (in all cases, the precise direction of change is given in the list of synapomorphies in Table S1).

The monophyly of the clade including gars, macrosemiids and “semionotiforms” has been demonstrated several times and is confirmed in this study. Two names have been proposed for this major clade: Semionotiformes [8], [9], [10], [11], [12] or Ginglymodi [13]. Since all gars, macrosemiids and “semionotiforms”, are included in this major clade, the names Ginglymodi Cope, 1872 [124], Lepisosteiformes Hay, 1929 [125], Semionotiformes Arambourg and Bertin, 1958 [38], and Macrosemiiformes Nelson, 2006 [35], are equally appropriate for this group, but the first name has priority over the others. However, Semionotiformes is the name adopted for the majority of authors referring to this major clade of neopterygian fishes and, thus, keeping this name would accord with the prevailing usage. Still, the name Lepisosteiformes has prevailing usage in reference to the gars (Lepisosteidae and Obaichthyidae). Therefore, since the usage of names is conflictive, I apply the principle of priority and follow Grande [13] adopting the name Ginglymodi for the major clade including gars, macrosemiids and “semionotiforms”. I keep the ordinal names of †Semionotiformes and Lepisosteiformes for the clades including the “semionotids” and the gars, respectively (see below).

It is worth noting that this usage of the name Ginglymodi is very different from that of Patterson [7]. Patterson took over this name, which, in agreement with Cope [124] only included the Lepisosteidae, to denote a clade representing the sister group of the Halecostomi. In the same work, Patterson concluded that the “semionotiforms” (including dapediids) represented a non-monophyletic assemblage, probably polyphyletic “placed as a basal grade in the Halecostomi” ([7]: 300). Bartram [39] further placed the †Macrosemiidae within the Halecostomi sensu Patterson [7] understanding the latter as the sister group of the equally ranked Ginglymodi (including Lepisosteidae only). Therefore, in Patterson [7] the usage of the name Ginglymodi is bound to the concept of Halecostomi and implies not only the acceptance of a monophyletic Halecostomi, but also that “semionotiforms” and macrosemiids are more closely related to halecomorphs and teleosts than they are to lepisosteids. Conversely, as used here, the name Ginglymodi does not imply, but agrees with the hypothesis of a monophyletic Holostei, which was proposed in all recent cladistic analyses including gars, macrosemiids, †”*Lepidotes*” (often actually †*Callipurbeckia minor*) and †*Semionotus* [12], [13], [108], [126]–[128]. The Ginglymodi is here characterized and defined according to a stem-based definition as follows:

#### Ginglymodi

The clade including all taxa more closely related to *Lepisosteus* than to *Dapedium*, *Amia* or *Pholidophorus*.

#### Diagnosis of ginglymodi

Neopterygian fishes characterized by the following combination of characters: Forward extension of the exoccipital around the vagus nerve (3(1))*; opistothic and intercalar bones absent (4(0), 5(0))*; presence of anterior infraorbital bones (34(1))*; premaxilla with nasal process (47(1))*; depth of suboperculum less than half the depth of operculum (67(1))*; gular plates absent (70(2))*; splint-like quadratojugal (17(1)) (ACCTRAN/DELTRAN); suboperculum with well-developed, tapering dorsally ascending process (64(1), 65(1)) (ACCTRAN/DELTRAN); long and narrow nasals (28(1)) (ACCTRAN); closed circumborbital ring (29(1)) (ACCTRAN); large supraorbital bones (31(1)) (ACCTRAN); numerous suborbital bones arranged in one row, which extends anteriorly below the orbit (42(2)) (ACCTRAN); supracleithrum with a concave articular facet for articulation with the posttemporal (73(1)) (ACCTRAN); series of denticles along the ridge between the branchial and lateral surfaces of the cleithrum (74(1)) (ACCTRAN); scale-like ray at the dorsal margin of the caudal fin (79(1)) (ACCTRAN).

The two main clades defined at the base of the Ginglymodi represent on the one hand the group containing †*Semionotus* and the macrosemiids (node A), and on the other hand the group containing †*Lepidotes* and the gars (node B). These major clades are here named Semionotiformes and Lepisosteiformes, respectively, and they are defined and characterized as follows:

#### †Semionotiformes

The clade including all taxa more closely related to †*Semionotus* than to †*Lepidotes*, *Lepisosteus*, †*Dapedium* or *Amia*.

#### Diagnosis of †Semionotiformes

Ginglymodian fishes with the following combination of characters: absence of endopterygoid dentition (15(1))*; frontals three or more times longer than their maximal width (23(1))*; narrow or tubular infraorbital bones forming the posterior border of the orbit (37(0))*; one or two rows of elongated scales at the posteroventral margin of the body lobe of the tail (82(1))*; circumborbital ring open anteriorly (29(0)) (ACCTRAN); small supraorbital bones (31(0)) (ACCTRAN); supracleithrum without a concave articular facet for articulation with the posttemporal (73(0)) (ACCTRAN); nasal bones long and narrow (28(1)) (DELTRAN); one or two rows of denticles along the ridge between the branchial and lateral surfaces of the cleithrum (74(1)) (DELTRAN); scale-like ray at the dorsal margin of the caudal fin (79(1)) (DELTRAN).

#### Lepisosteiformes

The clade including all taxa more closely related to *Lepisosteus* than to †*Semionotus*, †*Macrosemius*, †*Dapedium* or *Amia*.

#### Diagnosis of Lepisosteiformes

Ginglymodian fishes with the following combination of characters: ventral border of infraorbital series flexes abruptly dorsally at the anterior margin of the orbit (30(1))*; most anterior supraorbital bone trapezoidal, longest ventrally, contacting more than one infraorbital bone (32(1))*; scales with rostro-caudal articulation through anterior dorsal and ventral processes (86(2))*; supraorbital sensory canal marginally or not included in parietal bones (88(1))*; co-ossified vomers (10(1)) (ACCTRAN); three or more pairs of extrascapular bones (20(1)) (ACCTRAN); broad nasal bones (28(0)) (ACCTRAN); several series of denticles along the ridge between the branchial and lateral surfaces of the cleithrum (74(2)) (ACCTRAN); scale-like ray at the dorsal margin of the caudal fin absent (79(0)) (ACCTRAN); closed circumborbital ring (29(1)) (DELTRAN); large supraorbital bones (31(1)) (DELTRAN); supracleithrum with a concave articular facet for articulation with the posttemporal (73(1)) (DELTRAN).

Although the clade Semionotiformes has relatively low Bremer and Bootstrap support (1 and 44, respectively), it is nevertheless well defined with five unambiguous synapomorphies in the strict consensus tree (Fig. 16). The relationships between most taxa are often weakly supported within Semionotiformes, but a few monophyletic groups are well resolved. Further research will improve the knowledge and taxonomic composition of this major group. The monophyly of the †Macrosemiidae was already demonstrated by González-Rodríguez et al. [129] and González-Rodríguez & Reynoso [130], and here the three macrosemiid genera †*Propterus*, †*Macrosemius* and †*Notagogus* form a very well supported clade:

#### †Macrosemiidae

The clade including all taxa more closely related to †*Macrosemius* than to †*Semionotus*, †*Callipurbeckia*, †*Lepidotes* or *Lepisosteus*.

#### Diagnosis of †Macrosemiidae

Semionotiform fishes with the following combination of characters: dermal bones of the skull smooth or slightly ornamented (19(1))*; length of parietals less than one third the length of frontals (22(2))*; tubular antorbital portion of frontals (25(2))*; absence of suborbital bones (41(1))*; absence of supramaxilla (52(0))*; dentary without posteroventral process (56(0))*; posterior border of preoperculum notched ventrally (62(1))*; interoperculum small, remote from mandible (69(1))*; pectoral fin without fringing fulcra (75(1))*; eight lepidotrichia in the lower, non-axial lobe of the tail (80(1))*; basisphenoid absent (6(1)) (DELTRAN); posterior extension of parietals median to the single pair of laterally placed extrascapulars (21(1)) (DELTRAN); supraorbital sensory canal not included in parietals (88(2)) (DELTRAN).

†*Semionotus* is monophyletic in the strict consensus tree, and it is the sister group of a larger clade, which includes †*Semiolepis*, †*Paralepidotus*, †*Callipurbeckia*, †*Tlayuamichin*, and †*Macrosemimimus* (Fig. 16). This latter sister-group relationship is however very weakly supported (Bremer of 1 and Bootstrap of 23) and, thus, the family †Semionotidae is here restricted to †*Semionotus*:

#### †Semionotidae

The clade including all taxa more closely related to †*Semionotus bergeri* than to †*Callipurbeckia*, †*Macrosemius*, †*Lepidotes* or *Lepisosteus*.

#### Diagnosis of †Semionotidae

Semionotiform fishes with sphenotic bone with small dermal component (7(1))*; large basal fulcra in the dorsal and anal fins (78(1))*; eight lepidotrichia in the lower, non-axial lobe of the tail (80(1))*; conspicuous dorsal ridge scales with a high spine (83(1))*; triangular lateral expansion of antorbital portion of frontal present (27(1)) (ACCTRAN); closed circumborbital ring (29(1)) (ACCTRAN).

It was already mentioned that the genera †*Callipurbeckia* (Late Jurassic-earliest Cretaceous), †*Tlayuamichin* (Early Cretaceous) and †*Macrosemimimus* (Late Jurassic) also form a monophyletic clade (Fig. 16). The genera †*Paralepidotus* (Late Triassic) and †*Semiolepis* (Middle Triassic) are stem taxa to this clade. The overall resemblance between these five taxa, and between them and the macrosemiids is noticeable and has been already discussed in part by Schröder et al. [70]. Except for †*Tlayuamichin*, which would represent a late dispersion through a connecting Early Cretaceous seaway along coastal shallow marine environments of Europe and North America [32], these closely related taxa lived and evolved in the shallow marine environments around the Tethys Sea. Therefore, a new family †Callipurbeckidae including †*Callipurbeckia*, †*Tlayuamichin*, †*Macrosemimimus* and their stem taxa †*Paralepidotus* and †*Semiolepis* is defined and characterized as follows:

#### †Callipurbeckiidae

The clade including all taxa more closely related to †*Callipurbeckia* than to †*Macrosemius*, †*Semionotus*, †*Lepidotes* or *Lepisosteus*.

#### Diagnosis of †Callipurbeckiidae

Semionotiform fishes with small parietals, their length being less than one third the length of frontals (22(2))*; moderately tritoral dentition (55(1))*; high ascending process of suboperculum (66(1))*; presence of orbital sensory canal (89(1)) (ACCTRAN); supracleithrum with a concave articular facet for articulation with the posttemporal (73(1)) (DELTRAN). The following characters are absent in the stem taxa †*Paralepidotus* and †*Semiolepis*: two suborbital bones (42(1))*; several rows of denticles along the ridge between the branchial and lateral surfaces of the cleithrum (74(2))*; scale-like ray at the dorsal margin of the caudal fin absent (79(0))*; eight lepidotrichia in the lower, non-axial lobe of the tail (80(1))*; scales with rostro-caudal articulation with well developed anterior dorsal and ventral processes (86(2))*.

Within the clade Lepisosteiformes, the relationships between †*Lepidotes*, †*Scheenstia*, †*Isanichthys* and the remaining studied lepisosteiforms are unresolved in the strict consensus tree. Nonetheless, in 82% of the MPTs, †*Isanichthys* is more closely related to *Lepisosteus* than to †*Lepidotes*, and †*Scheenstia* and †*Lepidotes* are sister groups. The latter relationship suggests that the family Lepidotidae Owen, 1860 [15], probably represents a natural group, but the present analysis does not provide enough evidence supporting this hypothesis.

One of the most interesting results of the analysis is the rearrangement of many of the species so far classified in the genus †*Lepidotes*. A monophyletic †*Lepidotes* Agassiz, 1832 [25], is restricted to a few species from the Early Jurassic of central Europe, two of which have been included in this analysis: †*L*. *gigas* Agassiz, 1832 [25], and †*L*. *semiserratus* Agassiz, 1836 [58]. Most of the species previously referred to this genus that were included in this analysis do not join the monophyletic †*Lepidotes*, but other recently defined taxa (Fig. 16). The large, tritoral forms †”*Lepidotes*” *mantelli* Agassiz, 1833 [58], †”*Lepidotes*” *laevis* Agassiz, 1837 [58], and †”*Lepidotes*” *maximus* Wagner, 1863 [119], form a monophyletic group with †*Scheenstia zappi* López-Arbarello & Sferco, 2011 [37], and, thus, based on six unambiguous synapomorphies and very high Bootstrap and Bremer values, these three species are here refer to †*Scheenstia*. On the other hand, as explained before, †”*Lepidotes*” *minor* represents an independent genus †*Callipurbeckia* gen. nov., which is more closely related to †*Semionotus* and the macrosemiids within the Semionotiformes than to †*Lepidotes*.

The close relationship of †*Pliodetes* and †*Araripelepidotes* with the lepisosteids and obaichthyids sensu Grande [13] is very strongly supported. Most of the synapomorphies proposed by Grande [13] for his Lepisosteiformes are endocranial features unknown in †*Pliodetes* and †*Araripelepidotes* (Grande’s characters 2, 32, 59, 60, 63–65, 77). However, among the derived lepisosteiform features according to Grande [13] and although the junction between the supraorbital and infraorbital canal occurs in the dermosphenotic of †*Araripelepidotes* (AMNH 11813), the supraorbital canal does not penetrate the parietals in †*Araripelepidotes* or †*Pliodetes* (Fig. 9C–D; [21]: 112; pers. obs.). Furthermore, although the junction occurs in different bones, the general pattern followed by the supraorbital, infraorbital and temporal canals is basically the same in †*Araripelepidotes*, †*Pliodetes* and the gars. On the other hand, †*Pliodetes* shares with the gars some typically lepisosteiform features like the L-shaped preoperculum, the nasal processes of the premaxillae forming an external dermal component of the skull roof and bearing the supraorbital sensory canal, a mosaic of suborbital bones, and the absence of an independent interoperculum [13], [76]. †*Pliodetes* further presents two of the synapomorphies proposed by Grande [13] for the Obaichthyidae: large conical teeth firmly anchored to the surface of most of the dermal bones of the skull and rostral region elongated well anterior to the lower jaw symphysis by over 50% of the mandibular length (Grande’s characters 2 and 4 respectively). Also, the flank scales of †*Araripelepidotes* and †*Pliodetes* closely resemble the scales of obaichthyids in forming one or two large prominent spines at their posterior margin (Fig. 14). Consequently, according to the evidence discussed above, I consider †*Araripelepidotes* and †*Pliodetes* as basal gars and propose the name Lepisosteoidei for the clade defined at Node C in Figure 16.

#### Lepisosteoidei

The clade including all taxa more closely related to *Lepisosteus* or †*Pliodetes* than to †*Lepidotes*, †*Macrosemius* or †*Semionotus*.

#### Diagnosis of Lepisosteoidei

Lepisosteiform fishes with dorsal fin placed opposite to pelvic fins (1(2))*; rostral region extends well anterior to the dentary symphysis by more than 50% of mandibular length (9(1))*; dermal bones of the skull ornamented with firmly anchored large conical teeth (19(2))*; infraorbital bone or bones at the posteroventral corner of the orbit reach the preoperculum (36(2))*; quadrate laterally covered by infraorbital bones (40(1))*; mosaic of suborbital bones (42(3))*; supraorbital sensory canal in premaxillary nasal process (49(1))*; maxilla short, does not reach the coronoid process (50(1))*; absence of a supramaxilla (52(0))*; posteroventral process of the dentary absent (56(0))*; L-shaped preoperculum (60(2))*; operculum approximately as deep as wide (63(1))*; interoperculum small, remote from mandible (69(1))*; scales with a strong posteriorly directed spine (84(1))*; supraorbital canal does not enter parietals (88(2))*; co-ossified vomers (10(1)) (ACCTRAN); absence of autopalatine bone (11(1)) (ACCTRAN); elongate ectopterygoid that takes large part of the palatal surface (12(1), 13(1)) (ACCTRAN); large parietals, their length being about half the length of frontals (22(1)) (ACCTRAN); premaxillary nasal process forming an external dermal component of the skull roof (48(1)) (ACCTRAN); edentulous maxilla (53(1)) (ACCTRAN); marginal tooth row present on only the anterior one third or less of dentary (59(1)) (ACCTRAN); suboperculum more than half the depth of the operculum (67(0)) (ACCTRAN); denticles along the ridge between the branchial and lateral surfaces of the cleithrum absent (74(0)) (ACCTRAN); longitudinal articulation of the scales of the body present, but the anteroventral process is much smaller than the anterodorsal process (86(1)) (ACCTRAN); two pairs of extrascapular bones (20(1)) (DELTRAN).

Within Lepisosteoidei, the close relationships of †*Obaichthys* and †*Dentilepisosteus* with the Recet gars is very well supported. This arrangement is acknowledge as the Lepisosteiformes by Grande [13], but according to this study it represents an infra-ordinal rank and is here regarded as a superfamily Lepisosteoidea. The family †Obaichthyidae Grande, 2010 [13] is not recovered as a monophyletic group in this analysis (Figs. 16, 17). Nevertheless, Grande’s data matrix is more adequate than the matrix used for this study to solve the relationships within Lepisosteoidea because it includes more lepisoteoid taxa and more characters that are significant to establish those relationships. Therefore, I have no reason to question the results of the analysis carried out by Grande [13] and I accept the sister group relationships between †*Obaichthys* and †*Dentilepisosteus* in the clade †Obaichthyidae. Similarly, the family Lepisosteidae is here accepted in the more restricted sense of Grande [13], for which very high Bremer and Bootstrap values were obtained (Fig. 16).

#### Lepisosteoidea

The clade including all taxa more closely related to †*Obaichthys* or to *Lepisosteus* than to †*Pliodetes* or †*Lepidotes*.

#### Diagnosis of Lepisosteoidea

Lepisosteoid fishes with dorsal fin placed opposite to anal fin (1(1))*; absence of posttemporal fossa (2(0))*; basisphenoid absent (6(1))*; sphenotic with small dermal component (7(1))*; absence of posterior myodome (8(1))*; quadrate positioned in front of the orbit (16(1))*; opistocoelous vertebrae (71(1))*; six lepidotrichia in the lower, non-axial lobe of the tail (81(1))*; length of parietals less than one half but more than one third the length of frontals (22(0)) (ACCTRAN); supraorbital bones not particularly large (31(0)) (ACCTRAN); dermosphenotic does not reach the orbital margin (38(1)) (ACCTRAN); presence of maxillary teeth (53(0)) (ACCTRAN); robust ascending process of suboperculum (65(0)) (ACCTRAN); supracleithrum without concave articular facet for articultion with the posttemporal (73(0)) (ACCTRAN); vertical peg-and-socket articulation reduced or absent (85(1)) (ACCTRAN); deep groove housing the middle pit line in dermopterotic and parietal (90(1)) (ACCTRAN); absence of autopalatine bone (11(1)) (DELTRAN); elongate ectopterygoid that takes large part of the palatal surface (12(1), 13(1)) (DELTRAN); premaxillary nasal process forming an external dermal component of the skull roof (48(1)) (DELTRAN); marginal tooth row present on only the anterior one third or less of dentary (59(1)) (DELTRAN); longitudinal articulation of the scales of the body present, but the anteroventral process is much smaller than the anterodorsal process (86(1)) (DELTRAN).

#### †Obaichthyidae

The clade including all taxa more closely related to †*Obaichthys* than to *Lepisosteus*, †*Pliodetes* or †*Lepidotes*.

#### Diagnosis of †Obaichthyidae

See Grande ([13]: p. 661).

#### Lepisosteidae

The clade including all taxa more closely related to *Lepisosteus* than to †*Obaichthys* †*Pliodetes* or †*Lepidotes*.

#### Diagnosis of Lepisosteidae

See Grande ([13]: p. 26).

The phylogenetic relationships of the Early Cretaceous †*Neosemionotus* from Argentina remain unresolved at the base of Ginglymodi, but this genus is the sister group of lepisosteiforms in 89% of the MPTs (Fig. 17). The basal position of †*Neosemionotus* indicates that the history of the ginglymodians in South America is much longer than currently known. Ginglymodians are well represented in the Late Jurassic-Early Cretaceous of Brazil and Argentina [22], [131]–[132], but no reliable evidence of their presense have been found before that time [132–134).

### Character Evolution in Ginglymodi

In addition to the comments already made in the section “Discussion of Characters”, the evolution of certain characters deserve further and more detailed discussion. Two main features are distinct and stable among Ginglymodians: the presence of anterior infraorbitals and the absence of gular plates. The gulars however are also absent in other neopterygians like aspidorhynchids or osteoglossomorphs and more advanced teleosts [9], [63], but the anterior infraorbitals represent a very interesting feature uniquely derived in Ginglymodi. In Neopterygii the infraorbital bones are serial homologous and they develop in relation to the organs of the infraorbital sensory canal. The development of the dermal bones of the infraorbital sensory canal in *Amia calva* was described in detail by Pehrson [56] and can be summarized as follows. The formation of the canal bones in the skull of *Amia calva* starts early in the anterior part of the canal system and proceeds posteriorly. Pehrson defined two stages in the formation of these dermal ossifications. In the first stage the osteoblasts are formed and migrate under the epidermis to form the primary blastemas under each separate sense organ, and a stratum of osteoblasts under the future canal. The next stage is the formation of the secondary blastemas as a result of the gathering in the mesenchyma of the previously formed osteoblasts. These secondary blastemas do not always arise in connection with each separate sense organ and a single secondary blastema may be connected with more than one sense organ.

The rostral, antorbital and first infraorbital (lacrimal) bones (Fig. 7) develop first and nearly simultaneously. The primordia for the antorbital and first infraorbital (lacrimal) are already visible in a 11.5-mm specimen. The antorbital primordium is associated with the sense organs 3 to 6. The first infraorbital (lacrimal) primordium is associated with the organs 7 and 8. The two bones are already formed in a 12 mm specimen. Also in a 12 mm specimen, each of the two first sense organs on each side in the infraorbital series appear in connection with a separate, blastematic rostral primordium in the second stage of development. These four primordia will later fuse to form a single cylindrical rostral bone. The development of the more posterior infraorbitals and the dermosphenotic proceeds gradually posteriorly. A rudiment of the first of these elements is found under the sense organ 9 in a 12 mm specimen, and in a 13.8 mm specimen the primordium for the following bone is formed under organ 10. In a 16.1 mm specimen the primordia for the postorbitals, except the last element, and the dermosphenotic are formed, and, thus, the dermosphenotic forms earlier than the infraorbital bone immediately below it [56].

In the case of *Lepisosteus* there are three main reference works concerning the development of the dermal bones in the skull: Hammarberg ([80]; *L. platostomus*), Aumonier ([97]; *L. osseus*), and ([85]; *L. osseus* and *L. platostomus*). Among them, Hammarberg [80] includes the more detailed and complete description of the development of the bones around the infraorbital sensory canal. The first bones to develop in this series in *Lepisosteus* are the rostral, the antorbital, and the toothed infraorbitals. On each side, the rostral primordium appears in a 18.7 mm specimen of *L. platostomus*, in connection with the first neuromast in the infraorbital series [80]. In a 33.4 mm specimen the primordial rostral had extended backwards up to the third neuromast, the ethmoidal connection between the two infraorbital lines is established in a 44.2 mm specimen, and the cylindrical rostral is almost completely formed in a 65.4 mm specimen [80]. In *L. osseus*, Jollie [85] found the first evidence of the rostral in a 29 mm specimen. The antorbital primordium appears in a 18.7 mm specimen of *L. platostomus* and is associated to the neuromasts 4 to 7, and it is a well-formed tubular Y-shape bone in the 65.4 mm specimen [80]. In *L. osseus* the antorbital (lateral rostral in Jollie [85]) appears in a 28 mm specimen, below and anterior to the already formed first toothed infraorbital, and is associated to 4 or 5 neruomasts [85].

The first, most anterior primordial elements of toothed infraorbitals have no teeth and appear rather rapidly. There are already four primordia in a 18.7 mm specimen of *L. platostomus* [80]. The formation of the remaining toothed infraorbitals proceeds more slowly posteriorly. There are seven primordia in a 25 mm specimen and 11 primordia in a 49 mm specimen of *L. osseus* [85], and 13 and 14 primordia on each side of a 65.4 mm specimen of *L. platostomus* [80]. The teeth of the toothed infraorbitals form independently of the bones in the mouth margin below them. The teeth attach later to the toothed infraorbitals, starting at about the stage of a 39 mm specimen [84]. The vestigial maxilla also attach to the series of toothed infraorbitals at some stage between 75 and 150 mm specimens in *L. osseus*, and 85 and 125 mm specimens in *L. platostomus* [85].

The more posterior bones in the infraorbital series appear as a different series, which starts forming in a 54.2 mm specimen of *L. platostomus* [80], and this series is complete in a 75 mm specimen of *L. osseus* and an 85 mm specimen of *L. platostomus* [85]. The dermosphenotic forms some time before the infraorbital bones below it, and its blastema is found in the 54.2 mm specimen of *L. platostomus* [80].

The developmental patterns summarized before show that all the ossifications associated with the infraorbital sensory canal undergo the same process and serial homology can be assumed for the whole series from the rostral to the dermosphenotic [98]. However, due to their topographic relationships and early and simultaneous ontogenetic appearance, individual homology is accepted for the rostral and antorbital bones in *Amia* and *Lepisosteus* as already proposed by Hammarberg [80], Patterson [62], and Jollie [85], independently of the number of neuromasts associated with each bone. Similarly, the individual homology for the dermosphenotic bone in these taxa is supported by its position and out of turn development compared with the other infraorbital bones. The other infraorbital bones including the anterior infraorbitals, but not the toothed infraorbitals, developed gradually in the series and individual homologies cannot be established for any of them in particular. However, the topographic relationships of the ginglymodian anterior infraorbitals are unique among actinopterygians. Based on this topographic criterion, the hypothesis of primary homology has been proposed and tested in the cladistic analysis, resulting in an unambiguous and uniquely derived synapomorphy of the Ginglymodi. Therefore, within this clade, secondary homology is accepted for this portion of the infraorbital series, taken as a whole and restricted to the area between the antorbital and the first infraorbital bone forming the rim of the orbit.

In *Lepisosteus*, the most posterior toothed infraorbitals form later (at 60 to 65.4 mm stages) and ventral to the first, most anterior infraorbitals (at 54.2 mm stage) [80]. Therefore, there are two independent series: the series of toothed infraorbitals and the series of infraorbital bones, including the anterior infraorbitals (Fig. 7). It has been interpreted as a novelty of gars and, although serial homology with the infraorbital series ventral and posterior to the orbit is indicated by their development, this series of toothed infraorbitals has no known homologous structures in other actinopterygians. As shown by the cladistic analysis, the series of toothed infraorbitals appeared only once in the Lepisosteoidea and also represent a case of secondary homology.

A Splint-like quadratojugal is a unique feature of the Ginglymodi and their probably stem-taxon †Dapediidae. According to Patterson [83], the evolutionary trend in teleosts is towards the complete fusion and reduction of the quadratojugal, which might be limited to the spine-like posterior process of the compound quadrate in advanced teleosts, and a similar trend is observed in some semionotiforms [39]. The plate-like quadratojugal of basal actinopterygians contribute to the rigid upper jaw–cheek–palatoquadrate complex. The upper jaw becomes free and mobile in neopterygians and there are changes in the mode of suspension of the lower jaw in these fishes. Patterson [75] reinterpreted the “symplectic” of basal actinopterygians like †*Pteronisculus*, †*Boreosomus* and †*Australosomus* [135]–[136] and chondrosteans as an interhyal and proposed that the symplectic is a synapomorphy of the Neopterygii. I agree with Patterson and find no sustainable evidence for a symplectic outside Neopterygii. In neopterygians, the symplectic develops from the antero-ventral portion of the hyomandibular cartilage and contributes to the suspension of the lower jaw directly or via the quadrate and/or the quadratojugal [7]. The direct contribution to the suspension of the lower jaw occurs in the halecomorphs, in which the symplectic articulates directly with the lower jaw, as well as the quadrate. In the non-halecomorph neopterygians the symplectic contributes to the suspension indirectly. In teleosts the symplectic fits into a medial groove formed by the spine-like posterior process of the quadrate and the body of the quadrate. In ginglymodians and dapediids the symplectic articulates with the quadratojugal only (lepisosteids) or with the quadratojugal and the quadrate. In the latter case, the quadratojugal is a buttress firmly bound to the articular process of the quadrate (sometimes even partially fused to it) and the two bones form a medial groove that receives the symplectic. Among ginglymodians, the trend in lepisosteids is towards the enlargement of the quadratojugal, which becomes a bridge bone supporting the quadrate at its anterior end, and receiving the support of the symplectic at its posterior end. The suspension of the lower jaw is displaced forwards in lepisosteids; the quadrate places anterior to the orbit (which is a synapomorphy of this group; see character 21 and [76]) and the metapterygoid does not articulate with the hyomandibula [1], [80], [85]. Therefore, the methyostylic condition is only maintained through the symplectic-quadratojugal-quadrate bridge, which is furthermore tightly bound to the preoperculum via the quadratojugal. The condition of the symplectic is relatively poorly known in other neopterygians like pachychormiforms, aspidorhynchiforms, or pycnodontiforms. Patterson ([7]: fig. 18) reported a specimen of †*Pachychormus curtus*, in which the anterior end of the symplectic is partially fused to the inner face of the quadrate. Brito [137] described and illustrated a halecomorph-like symplectic articulating with the lower jay in †*Vinctifer* (†Aspidorhynchidae). In pycnodontiforms the symplectic is also directly involved in the lower jaw articulation resembling the condition in halecomorphs [138].

The presence of numerous suborbital bones has been considered a primitive feature in ginglymodians and dapediids [7]. However, although suborbital bones are also present in numerous basal non-neopterygian actinopterygians, only in ginglymodians and dapediids the suborbitals are covering a large portion of the cheek between the circumborbital bones and the preoperculum. In basal actinopterygians the suborbitals are small and restricted to a small area limited by the large preoperculum and the large posterior plate of the maxilla (e.g. several basal actinopterygians in Gardiner & Schaeffer [139], †Pseudobeaconiidae in López-Arbarello & Zavattieri [101], †Scanilepiformes in Xu & Gao [140]). The presence of a series of suborbitals more or less arranged in one row, as found in †*Dapedium* and many ginglymodians represents a novelty of the clade (†*Dapedium* (Ginglymodii)) and the primitive condition within Ginglymodi. In Lepisosteiforms the trend is towards a mosaic of suborbitals, which is a synapomorphy of the Lepisosteoidei. Quite the opposite, the trend in †Semionotiformes is towards the reduction in number of suborbitals (one suborbital in †*Semionotus*, †*Paralepidotus* and †*Semiolepis*, or two suborbitals in †Callipurbeckiidae), which are restricted to the area of the cheek posterior to the orbit only, or the complete absence of suborbitals in macrosemiids. Although the species was not included in this analysis, †”*Lepidotes*” *pankowskii* Forey et al., 2011 [36], with an extreme condition in which the series of suborbital bones extends further anteriorly below the series of anterior infraorbitals, is most probably a lepisosteiform.

The nasal process of the premaxilla present in Ginglymodi and halecomorphs, but not in †*Dapedium*, is a well-developed process mesial to the nasal sac, which is covering the adjacent ethmoidal endoskeleton and is perforated by a relatively large foramen for the passage of the olfactory nerve. Wiley [76], following Hammarberg [80] and Amounier [97]), concluded that the premaxillary nasal process of gars is not homologous with that of the amiiforms, and Olsen [53] and Olsen & McCune [8] interpreted the elongate nasal process in lepisosteids, “semionotids” and macrosemiids as a synapomorphy of the Semionotiformes. Patterson [62] however, concluded that the nasal processes of the premaxillae of all non-teleostean neopterygians are basically homologous.

**Figure 26:**
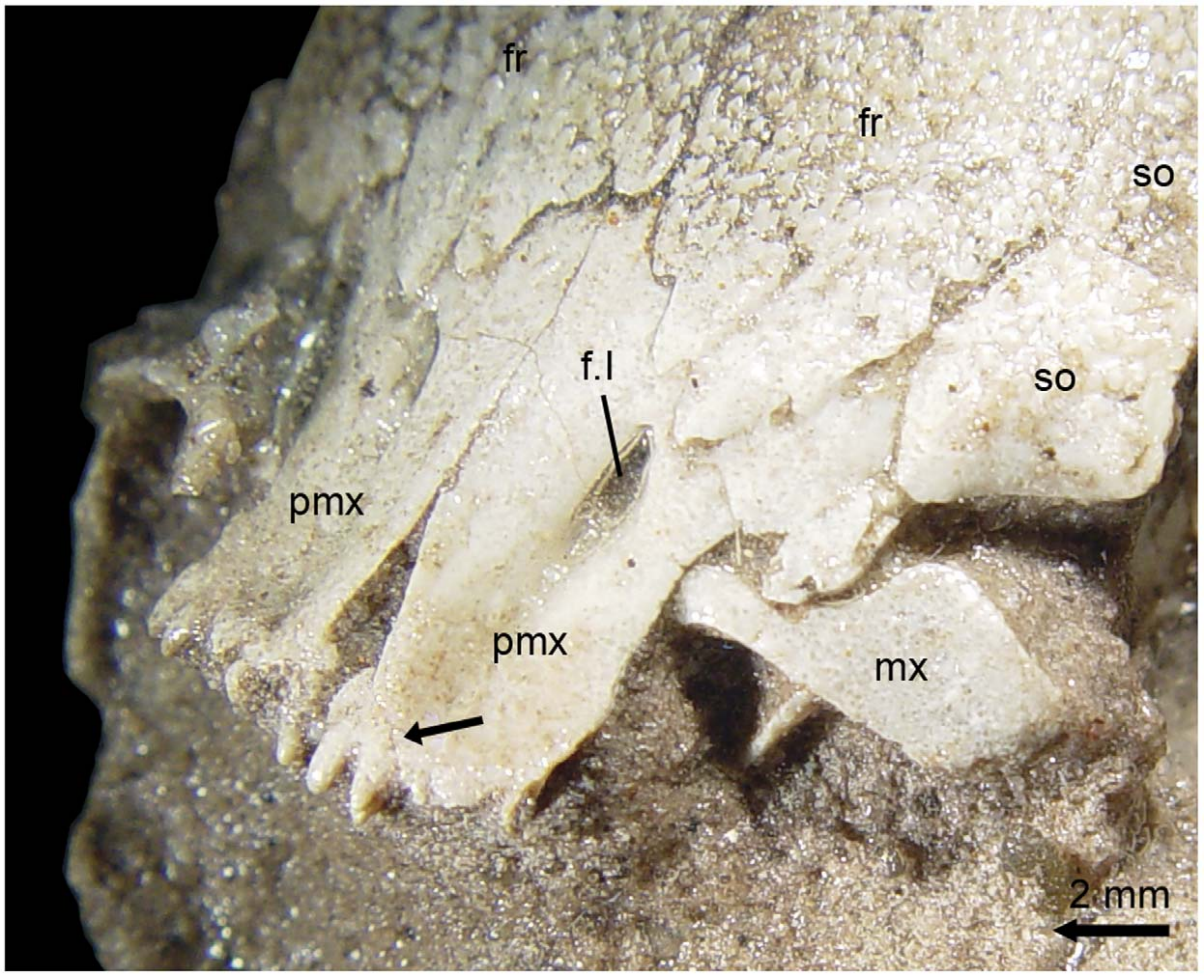
Premaxillary nasal processes in †*Pliodetes nigeriensis* Wenz, 1999 [**21**] (MNHN-GDF-1314). Abbreviations: f.I, foramen for the olfactory nerve; fr, frontal; mx, maxilla; pmx, premaxilla; so, supraorbital. Black arrow points to the suture between the nasal process and the toothed portion of the left premaxilla. Scale bar points anteriorly.

Bjerring has shown that the premaxillary nasal process corresponds to the rhinal bone, defined as the “ascending infrapharyngeal dental plate of the first-metamere branchial moiety”, which fuses with the premaxilla during the ontogeny ([141]: 200). Bjerring’s conclusion is based on ontogenetic studies of *Amia* [80], [142]–[145]. I have found further evidence in ginglymodians favouring the homology between the nasal process of the premaxilla and the rhinal bone. In two specimens of †*Pliodetes nigeriensis* (MNHN GDF 1275 and 1314; Fig. 26) and in the lectotype of †*Callipurbeckia minor* (BGS GSM 27975), the nasal process is not completely fused to the premaxilla. However, this evidence is enough only to propose a hypothesis of primary homology between the nasal processes of halecomorphs and ginglymodians. The current analyisis is not enough to propose hypothesis of secondary homology for these structures, which should be explored in a more comprehensive cladistic analysis of basal neopterygian lineages.

Patterson ([62]: 503) questioned Bjerring’s hypothesis of homology. Based on the shape of the premaxilla in some more primitive actinopterygians like †*Perleidus* and a probable parasemionotid from East Greenland, which have a small “ascending process”, Patterson argued that the nasal process of the holostean fishes develops originally as an outgrowth of the premaxilla, although he admits that the nasal process ossifies independently in the ontogeny of *Amia*. A small “ascending process” is certainly present in the premaxillae of at least some perleidiform fishes and some basal neopterygians like †*Dapedium*, but there is no evidence that such a process is homologous with the large nasal process of amiiforms and semionotiforms, which is distinctly perforated or notched for the passage of the olfactory nerve. Furthermore, the small process in the premaxilla of basal actinopterygians is more likely to be homologous to the premaxillary articular process of teleost than the nasal process of halecomorphs and semionotiforms.

According to Patterson ([7]: 503), the ascending process of the premaxilla of teleosts is not homologous with the nasal process defined above. His arguments however are very confusing (see Jollie [85]: 369) and this case of homology has never been explored in detail. The homologies of the ascending and articular processes of the premaxilla of advanced teleosts are still unclear. The premaxilla in †*Pholidophorus* and other basal teleosts has a small process, which is morphologically similar to the process in †*Perleidus* and might be homologous to the articular process of more advanced teleosts, which would have been acquired earlier than the ascending process [81], [146]. As is the case of the premaxillary nasal process, the premaxillary ascending process of teleosts also develops as an independent ossification, which later fuses to the premaxilla [81], [85], [146]. However, this fact does not imply homology because the two processes probably originate from different tissues or primordia. Also, the bones identified as “lateral dermethmoids” in pholidophorids by Patterson [7], [62] have similar characteristics and relationships as the rhinal bone of Bjerring [141] and, according to Patterson [62] they become part of the compound metapterygoid of more advanced teleosts, which resembles the nasal process of halecomorphs and ginglymodians. In any case, a nasal process with the characteristics described above is absent in teleosts and this feature is not problematic for the cladistic analysis conducted here. However, solving the question of homology between the ascending process in teleosts and the nasal process in halecomorphs and ginglymodians is critical to the so-called “gar-*Amia*-teleost” problem, i.e. the monophyly of Halecostomi vs. the monophyly of Holostei [13].

The peculiar morphology of the body scales of †*Lepidotes* has led to the identification of a countless number of isolated scales from Mesozoic sediments all around the world in this genus. In addition to the dorso-ventral peg-and-socket articulation typical of the rhomboid scales, the scales of †*Lepidotes gigas* and the other species in this genus, present two anterior processes involved in an rostro-caudal or longitudinal articulation: the anterior dorsal process and the anterior ventral process (Fig. 16B). Not surprisingly, the same kind of scales are found in the closely related genus †*Scheenstia*, but their distribution is even wider and similar scales are present in †*Callipurbeckia*, †*Araripelepidotes* and †*Masillosteus* (Fig. 15C, F). The cladistic analysis shows that this rostro-caudal mode of scale articulation first appeared in the clade (†Semionotiformes, Lepisosteiformes) and is absent in the most basal ginglymodians. In both lineages, the anterior ventral process is secondarily reduced: in the Lepisoteoidei among lepisosteiforms and in the macrosemiids among semionotiforms.

Jain [74] distinguished three kinds of dentition in “semionotids”: non-tritoral, moderately tritoral, and strongly tritoral. According to Jain [74] tritoral dentition is recognized by the combination of four characters: “Firstly, the width of the tooth relative its height, some non-tritoral teeth do have tumid crowns, but these are set on moderately long pedicles. Secondly, the shape of the crown, those of the tritoral species being typically broad and with a very bluntly conical termination when newly erupted. Thirdly, the relative thickness of the enamel, which is thick in the non-tritoral forms, thin on the tritoral teeth. Fourthly, the wear on the teeth, which is absent in non-tritoral forms, variably developed in tritoral species, perhaps due to different rates of tooth replacement and to types of diet. All four of these characters must be used in deciding on the nature of the inner dentition” ([74]: 30). To distinguish between moderately and strongly tritoral dentitions, Jain ([74]: Table 9) also used other morphological characters of the lower jaw and palate, which he found associated to the type of dentition: the depth of the jaw symphysis, the thickness and relative size of the tooth-bearing area of the coronoid bones, the presence of co-ossified vomers, and the relative length of the tooth-bearing areas on the vomers. The association of these features with one or the other kind of dentition is however ambiguous, and some of them are rarely preserved or visible in the fossils. Although tritoral and semitritoral dentitions may occur together with co-ossified vomers, fishes like †*Lepidotes microrhis* [72] or the basal teleost have co-ossified vomers, but lack tritoral dentition, and fishes like †*Tlayuamichin*, †*Macrosemius* or †*Paralepidotus* have semitritorial dentition and separate paired vomers. The actual shape of the coronoid bones or their tooth-bearing areas, as well as the relative length of the tooth-bearing areas on the vomers are only rarely observable because these bones are usually partially to mostly hidden. However, the depth of the jaw symphysis does seem to be positively correlated with the presence of extremely tritoral dentition. Although †*Scheenstia zappi* has strongly tritoral dentition, but moderately deep jaw symphysis, the other fishes with this kind of dentition (†*Scheenstia maximus*, †*S*. *laevis*, †*S*. *mantelli* and †*Macrosemimimus lennieri*) have very deep mandibular symphyses. Although it is interesting to explore the potential co-occurrence of the four characters proposed by Jain [74] in his Table 9 in further detail, the tooth morphology alone is sufficient to distinguish between a strongly tritoral dentition, in which there is no styliform tooth, and a moderately tritoral dentition, in which the marginal teeth are styliform and the teeth are gradually more tritoral towards the midline.

Woodward [95] and Jain [74] analysed the variation in the shape and proportion of the mouth and jaw bones in several species that at those times were classified in the genus †*Lepidotes*, including several taxa studied for this analysis: †*Lepidotes elvensis* and †*L*. *semiserratus*, †*Callipurbeckia minor*, †*C*. *notopterus*, †*Scheenstia mantelli*, and the type specimens of †”*Lepidotes*” *toombsi* (junior synonym of †*Macrosemimimus lennieri* according to [70]). Woodward proposed the general tendencies through time towards more tritoral dentition, shorter jaws, smaller mouth, increasing number of extrascapulars and suborbitals and a nearly straight inter-frontal suture. After measuring as many specimens as possible, Jain was unable to confirm the first three and the last of these tendencies and I find no clear tendencies regarding those features either. However, the shape of the lower jaw is highly variable among ginglymodians and since variation in the jaw lever ratios has been shown to reflect variation in jaw closing speeds, force and modes of feeding [147], this observed morphological variation is most probably related with differences in the diet (e.g. those fishes with strongly tritoral dentition have very deep coronoid processes and very massive lower jaws). In an attempt to account for such a meaningful morphological variation, biomechanical comparisons were challenged by the difficulties of establishing the proper point of insertion of the ligaments and muscles involved in the lower jaw lever model of fishes in these Mesozoic fossils. Furthermore, many fossil ginglymodians are fully articulated and actually conceal the jaw joint, so that the most anterior tooth is only rarely preserved in situ and complete, making it impossible to measure the out-lever moment arm of the jaw.

Jain [74] agreed with Woodward [95] concerning the putative increase in the number of suborbital and extrascapular bones. I already commented on the variation on the number and arrangement of suborbital bones in my data set, and rather than an increase in number, the observed tendencies relate to the pattern of suborbitals. On the other hand, the variation in the number of extrascapular bones, although taxonomically useful because the number of extrascapulars is stable for a given genus, it shows no phylogenetic signal or temporal correlation. The species of †*Scheenstia*, covering a temporal range from the Late Jurassic (Kimmeridgian) to the Early Cretaceous (Hauterivian-Barremian) have the maximal number of extrascapulars (four pairs or more), as well as the Triassic †*Semiolepis*, the Early Jurassic †*Dapedium* and the Recent *Atractosteus*. A single pair of extrascapular is found from the Triassic †*Sangiorgioichthys aldae*, †*Luoxiongichthys*, †*Paralepidotus* and †*Semionotus bergeri*, the Early Jurassic †*Lepidotes*, and the Late Jurassic callipurbeckiids. Two pairs of extrascapulars are present in most lepisosteoids from the Early Cretaceous to Recent, the Early Cretaceous †*Tlayuamichin*, the Early Jurassic †*Semionotus capensis* and the Middle Triassic †*Sangiorgioichthys sui*.

Finally, the presence of dorsal ridge scales was proposed as a synapomorphy shared by †*Lepidotes* and †*Semionotus* by Olsen & McCune [8]. Thies [19] proposed the presence of inconspicuous dorsal ridge scales, without a posterior spine, as a diagnostic feature of †*Lepidotes*. The present analysis shows that, except for †*Neosemionotus*, the relationships of which are not resolved, conspicuous dorsal ridge scales occur only in semionotiforms. The presence of dorsal ridge scales with a low posterior spine has a rather patchy distribution within this clade (present in †*Sangiorgioichthys*, †*Semiolepis*, †*Callipurbeckia* and †*Tlayuamichin*), but ridge scales with high posterior spines are uniquely derived in †*Semionotus*.

## Conclusions

The Neopterygii are the largest and most important group of fishes, and they also include the teleosts and thus some 50% of modern vertebrate diversity. However, the origin and early evolution of neopterygians is still poorly understood, as are the interrelationships of their main lineages. This situation is the consequence of several interrelated factors: the unresolved question of the out-group to Neopterygii, many problematic cases of homology among basal neopterygians, and the still very incomplete knowledge of several basal lineages, most of them extinct, which is not necessarily due to the lack of well preserved fossils, but rather the paucity of studies. The “semionotiforms” have certainly been one these so far poorly known fossil groups. The main goal of this and my previous taxonomic work on “semionotiforms” [22]–[23], [32], [34], [36]–[37], [70] has been to change this situation providing empirical information as much detailed as possible on the anatomy of these fishes. The phylogenetic relationships obtained through the cladistic analysis presented here, as any other cladogram, only represent a hypothesis that will hopefully be improved by future research.

The question of the living sister group of teleosts is usually referred to as the “gar-*Amia*-teleost” problem (i.e., the “Halecostomi vs. Holostei”), because the living sister group of teleosts would be *Amia* under the Halecostomi hypothesis, or the group (*Amia*, *Lepisosteus*) under the Holostei hypothesis. As a result of my research on “semionotiforms”, I found that the “Halecostome paradigm” has been misled by the erroneous interpretation of the evolution of certain morphological characters. Under the “Halecostome paradigm” interpretations of primary homology and character polarity have mainly been based in comparisons with modern teleosts (e.g. [7], [148], [149]). With the increasing knowledge of the anatomy of Palaeozoic and early Mesozoic actinopterygians, and the incorporation of this information in cladistic analyses, we now begin to understand the right direction of change in the evolution of several morphological characters, which leads many authors to return to the Holostei hypothesis of Huxley [150]. Recent morphological studies like the ones by Hurley et al. [108] and Grande [13] are very important because they have seriously questioned the “Halecostome paradigm”. In particular Grande [13] provided thorough and detailed anatomical information on lepisosteiforms, which is essential and cannot be ignored in future research around the “gar-*Amia*-teleost” problem. This work completes Grande’s study providing anatomical information on basal ginglymodians and, thus, is a contribution to our understanding of the origin and phylogenetic relationships of basal neopterygians. My studies on Mesozoic ginglymodians led me to confirm Patterson’s [7] observation that these fishes show morphological affinities with both halecomorphs and teleosts. Therefore, although the hypothesis of the Holostei is still far from being demonstrated, we are at the beginning of a new and fruitful era in palaeoichthyological research. Detailed anatomical studies of non-teleostean actinopterygians (e.g. [8], [13], [39], [54], [62], [151]–[152]) are going to be the foundation for the re-evaluation of our current hypotheses of homology and the compilation of large data sets, which are the only valid way to test the hypotheses of the Halecostomi vs. the Holostei. In particular, more studies on Triassic and Jurassic Neopterygians are utterly needed.

Except for the classification of gars, which is taken from Grande [13], the following classification of ginglymodians is based on the relationships obtained in this cladistic analysis (Figs. 16, 25).

NEOPTERYGII Regan, 1923 [1].

GINGLYMODI Cope, 1872 [124].

†*Neosemionotus* Bocchino, 1973 [27].

†SEMIONOTIFORMES Arambourg & Bertin, 1958 [38] new usage.

†*Luoxiongichthys* Wen et al., 2012 [71].

†*Sangiorgioichthys* Tintori & Lombardo, 2007 [33].

†SEMIONOTIDAE Woodward, 1890 [14].

†*Semionotus* Agassiz, 1832 [25].

†CALLIPURBECKIIDAE fam. nov.

†*Paralepidotus* Stolley, 1920 [28].

†*Semiolepis* Lombardo & Tintori, 2008 [29].

†*Callipurbeckia* gen. nov.

†*Tlayuamichin* López-Arbarello & Alvarado-Ortega, 2011 [32].

†*Macrosemimimus* Schröder et al., 2012 [70].

†MACROSEMIIDAE Thiollière, 1858 [153] (only the taxa included in the analysis are here classified).

†*Macrosemius* Agassiz, 1834 [58].

†*Propterus* Agassiz, 1834 [58].

†*Notagogus* Agassiz, 1833 [58].

LEPISOSTEIFORMES Hay, 1929 [125].

†*Lepidotes* Agassiz, 1832 [25].

†*Isanichthys* Cavin & Suteethorn 2006 [10].

†*Scheenstia* López-Arbarello & Sferco, 2011 [37].

LEPISOTEOIDEI.

†*Araripelepidotes* Santos, 1990 [24].

†*Pliodetes* Wenz, 1999 [21].

LEPISOSTEOIDEA.

†OBAICHTHYIDAE Grande, 2010 [13].

†*Dentilepisosteus* Grande, 2010 [13].

†*Obaichthys* Wenz & Brito, 1992 [93].

LEPISOSTEIDAE Cuvier, 1825 [154].

*Atractosteus* Rafinesque 1820 [155].

*Lepisosteus* Lacépède, 1803 [156].

†*Masillosteus* Micklich & Klappert, 2001 [94].

The monophyly of Ginglymodi is here confirmed and even better supported than in previous studies. Among the synapomorphies supporting the monophyly of ginglymodians, the presence of anterior infraorbital bones is uniquely derived in this clade and represents the main distinctive feature.

Gars, macrosemiids and “semionotiforms” have been rearranged with respect to previous classifications. Several “semionotiform” or “semionotid” taxa, i.e. †*Lepidotes*, †*Scheenstia*, †*Araripelepidotes* and †*Pliodetes*, are actually more closely related to Lepisosteidae than to †*Semionotus* and are, thus, lepisosteiforms (Figs. 16, 17). The order Lepisosteiformes is thus here expanded, redefined and diagnosed in order to include these taxa, and two subordinal very well supported clades are named: the Lepisosteoidei and the Lepisosteoidea (Fig. 25).

The macrosemiids are neither more derived [8], [9], nor more primitive [12], [13], [149] than “semionotiforms”; they are well nested within the taxa closely related to †*Semionotus* or “semionotids” (Fig. 16). Therefore, macrosemiids are not distinguished as a separate lineage or order Macrosemiiformes, but the family is here referred to the †Semionotiformes.

The order †Semionotiformes is restricted here to the taxa more closely related to †*Semionotus* than to lepisosteids. Similarly, the family †Semionotidae is restricted to the genus †*Semionotus*, and a new semionotiform family is named, the Callipurbeckiidae, for the clade (†*Paralepidotus* (†*Semiolepis* (†*Macrosemimimus* (†*Callipurbeckia minor* new comb., †*Tlayuamichin*)))) (Fig. 16).

Last, but not less important, the monophyly of †*Lepidotes* is here resolved by restricting this genus to several Early Jurassic species known from central Europe. Other species previously referred to †*Lepidotes* have shown different phylogenetic relationships and are here referred to †*Scheenstia*, or the new genus †*Callipurbeckia* (Figs. 16, 25). The other numerous nominal species that have been referred to †*Lepidotes*, pending their taxonomic revisions, should thus be treated as incertae genus (e.g. †”*Lepidotes*” *deccanensis*, †”*Lepidotes*” *latifrons*, †”*Lepidotes*” *ovatus*, †”*Lepidotes*” *pankowskii*, †”*Lepidotes*” *piauhiensis*, †”*Lepidotes*” *souzai*, †”*Lepidotes*” *microrhis*, †”*Lepidotes*” *tanyrhis*, etc.).

## Supporting Information

Table S1 List of synapomorphies.
(DOCX)

Text S1 List of material examined.
(DOCX)

Text S2 Data matrix for phylogenetic analysis.
(DOCX)
