# Supplementary material for: Phylogenetic Interrelationships of Ginglymodian Fishes (Actinopterygii: Neopterygii)
Source: PLoS One. 2012 Jul 11;7(7):e39370. doi: 10.1371/journal.pone.0039370 (PMC3394768; doi:10.1371/journal.pone.0039370)
Supplement: Table S1 — List of synapomorphies. (DOCX) [file pone.0039370.s001.docx]

# List of synapomorphies

| Brunch | Ch. | CI | Direction of change | | |
| --- | --- | --- | --- | --- | --- |
|  |  |  | Unambiguous | ACCTRAN | DELTRAN |
| Ginglymodi | 3 | 1.000 | 0 ==> 1 |  |  |
| (Bremer 4, Bootstrap 76) | 4 | 0.500 | 0 ==> 1 |  |  |
|  | 5 | 1.000 | 0 ==> 1 |  |  |
|  | 17 | 0.600 |  | 0 ---> 1 | 0 ---> 1 |
|  | 28 | 0.500 |  | 0 ---> 1 |  |
|  | 29 | 0.167 |  | 0 ---> 1 |  |
|  | 31 | 0.200 |  | 0 ---> 1 |  |
|  | 34 | 1.000 | 0 ==> 1 |  |  |
|  | 42 | 0.500 |  | 0 ---> 2 | 0 ---> 2 |
|  | 47 | 0.500 | 0 ==> 1 |  |  |
|  | 64 | 0.333 |  | 0 ---> 1 | 0 ---> 1 |
|  | 65 | 0.200 |  | 0 ---> 1 | 0 ---> 1 |
|  | 67 | 0.167 | 0 ==> 1 |  |  |
|  | 70 | 0.667 | 0 ==> 2 |  |  |
|  | 73 | 0.167 |  | 0 ---> 1 |  |
|  | 74 | 0.286 |  | 0 ---> 1 |  |
|  | 79 | 0.200 |  | 0 ---> 1 |  |
| Lepisosteiformes | 10 | 0.250 |  | 0 ---> 1 |  |
| (Bremer 1, Bootstrap 48) | 20 | 0.200 |  | 0 ---> 1 |  |
|  | 28 | 0.500 |  | 1 ---> 0 |  |
|  | 29 | 0.167 |  |  | 0 ---> 1 |
|  | 30 | 0.500 | 0 ==> 1 |  |  |
|  | 31 | 0.500 |  |  | 0 ---> 1 |
|  | 32 | 0.500 | 0 ==> 1 |  |  |
|  | 73 | 0.167 |  |  | 0 ---> 1 |
|  | 74 | 0.286 |  | 1 ---> 2 |  |
|  | 79 | 0.200 |  | 1 ---> 0 |  |
|  | 86 | 0.333 | 0 ==> 2 |  |  |
|  | 88 | 0.400 | 0 ==> 1 |  |  |
| Lepisosteoidei | 1 | 0.429 | 0 ==> 2 |  |  |
| (Bremer >4, Bootstrap 99) | 9 | 0.500 | 0 ==> 1 |  |  |
|  | 10 | 0.250 |  | 0 ---> 1 |  |
|  | 11 | 1.000 |  | 0 ---> 1 |  |
|  | 12 | 0.500 |  | 0 ---> 1 |  |
|  | 13 | 1.000 |  | 0 ---> 1 |  |
|  | 19 | 0.250 | 0 ==> 2 |  |  |
|  | 20 | 0.200 |  |  | 0 ---> 1 |
|  | 22 | 0.222 |  | 0 ---> 1 |  |
|  | 36 | 0.222 | 0 ==> 2 |  |  |
|  | 40 | 0.500 | 0 ==> 1 |  |  |
|  | 42 | 0.500 | 2 ==> 3 |  |  |
|  | 48 | 0.500 |  | 0 ---> 1 |  |
|  | 49 | 1.000 | 0 ==> 1 |  |  |
|  | 50 | 0.667 | 0 ==> 1 |  |  |
|  | 52 | 0.400 | 1 ==> 0 |  |  |

| Brunch | Ch. | CI | Direction of change | | |
| --- | --- | --- | --- | --- | --- |
|  |  |  | Unambiguous | ACCTRAN | DELTRAN |
| Lepisosteoidei | 53 | 0.200 |  | 0 ---> 1 |  |
| (Bremer >4, Bootstrap 99) | 56 | 0.200 | 1 ==> 0 |  |  |
|  | 59 | 0.500 |  | 0 ---> 1 |  |
|  | 60 | 0.500 | 1 ==> 2 |  |  |
|  | 63 | 0.500 | 0 ==> 1 |  |  |
|  | 67 | 0.167 |  | 1 ---> 0 |  |
|  | 69 | 0.200 | 0 ==> 1 |  |  |
|  | 74 | 0.286 |  | 2 ---> 0 |  |
|  | 84 | 0.500 | 0 ==> 1 |  |  |
|  | 86 | 0.333 |  | 2 ---> 1 |  |
|  | 88 | 0.400 | 1 ==> 2 |  |  |
| Lepisosteoidea | 1 | 0.429 | 2 ==> 1 |  |  |
| (Bremer 2, Bootstrap 83) | 2 | 0.500 | 1 ==> 0 |  |  |
|  | 6 | 0.500 | 0 ==> 1 |  |  |
|  | 7 | 0.250 | 0 ==> 1 |  |  |
|  | 8 | 1.000 | 0 ==> 1 |  |  |
|  | 11 | 1.000 |  |  | 0 ---> 1 |
|  | 12 | 0.500 |  |  | 0 ---> 1 |
|  | 13 | 1.000 |  |  | 0 ---> 1 |
|  | 16 | 1.000 | 0 ==> 1 |  |  |
|  | 22 | 0.222 |  | 1 ---> 0 |  |
|  | 31 | 0.200 |  | 1 ---> 0 |  |
|  | 38 | 0.333 |  | 0 ---> 1 |  |
|  | 48 | 0.500 |  |  | 0 ---> 1 |
|  | 53 | 0.200 |  | 1 ---> 0 |  |
|  | 59 | 0.500 |  |  | 0 ---> 1 |
|  | 65 | 0.200 |  | 1 ---> 0 |  |
|  | 71 | 1.000 | 0 ==> 1 |  |  |
|  | 73 | 0.167 |  | 1 ---> 0 |  |
|  | 81 | 1.000 | 0 ==> 1 |  |  |
|  | 85 | 0.333 |  | 0 ---> 1 |  |
|  | 86 | 0.333 |  |  | 2 ---> 1 |
|  | 90 | 0.250 |  | 0 ---> 1 |  |
| Lepisosteidae | 9 | 0.500 | 1 ==> 0 |  |  |
| (Bremer >4, Bootstrap 97) | 19 | 0.250 | 2 ==> 0 |  |  |
|  | 31 | 0.200 |  | 0 ---> 1 |  |
|  | 38 | 0.333 |  | 1 ---> 0 |  |
|  | 39 | 1.000 | 0 ==> 1 |  |  |
|  | 50 | 0.667 | 1 ==> 2 |  |  |
|  | 54 | 1.000 | 0 ==> 1 |  |  |
|  | 61 | 0.500 | 0 ==> 1 |  |  |
|  | 65 | 0.200 |  |  | 1 ---> 0 |
|  | 67 | 0.167 |  |  | 1 ---> 0 |
|  | 68 | 0.333 | 1 ==> 0 |  |  |
|  | 73 | 0.167 |  | 0 ---> 1 |  |
|  | 84 | 0.500 | 1 ==> 0 |  |  |
|  | 85 | 0.333 |  |  | 0 ---> 1 |
|  | 87 | 1.000 | 0 ==> 1 |  |  |
|  | 90 | 0.250 |  |  | 0 ---> 1 |

| Brunch | Ch. | CI | Direction of change | | |
| --- | --- | --- | --- | --- | --- |
|  |  |  | Unambiguous | ACCTRAN | DELTRAN |
| (*Lepisosteus*, *Atractosteus*) | 14 | 1.000 | 0 ==> 1 |  |  |
| (Bremer >4, Bootstrap 100) | 23 | 0.200 | 0 ==> 1 |  |  |
|  | 33 | 1.000 | 0 ==> 1 |  |  |
|  | 37 | 0.250 | 2 ==> 1 |  |  |
|  | 57 | 1.000 | 0 ==> 1 |  |  |
|  | 59 | 0.500 | 1 ==> 0 |  |  |
|  | 89 | 0.250 | 0 ==> 1 |  |  |
| *Scheenstia* | 20 | 0.200 | 1 ==> 2 |  |  |
| (Bremer 2, Bootstrap 87) | 44 | 1.000 | 0 ==> 1 |  |  |
|  | 50 | 0.667 |  | 0 ---> 1 |  |
|  | 51 | 0.250 |  | 0 ---> 1 |  |
|  | 53 | 0.200 |  | 0 ---> 1 |  |
|  | 55 | 0.250 | 0 ==> 2 |  |  |
|  | 72 | 1.000 | 0 ==> 1 |  |  |
|  | 89 | 0.250 | 0 ==> 1 |  |  |
|  | 90 | 0.250 | 0 ==> 1 |  |  |
| (*Lepidotes* *maximus*, *Lepidotes laevis*, | 10 | 0.250 |  |  | 0 ---> 1 |
| *Lepidotes mantelli*) | 23 | 0.167 |  | 0 ---> 1 |  |
| (Bremer 1, Bootstrap 82) | 50 | 0.667 |  |  | 0 ---> 1 |
|  | 51 | 0.250 |  |  | 0 ---> 1 |
|  | 53 | 0.200 |  |  | 0 ---> 1 |
|  | 58 | 0.333 | 0 ==> 1 |  |  |
|  | 65 | 0.200 |  | 1 ---> 0 |  |
|  | 74 | 0.286 |  | 2 ---> 1 |  |
| *Lepidotes* | 20 | 0.200 |  | 1 ---> 0 |  |
| (Bremer 2, Bootstrap 77) | 30 | 0.500 |  | 1 ---> 0 |  |
|  | 35 | 0.500 | 0 ==> 1 |  |  |
|  | 37 | 0.250 | 2 ==> 1 |  |  |
|  | 85 | 0.333 | 0 ==> 1 |  |  |
|  | 90 | 0.250 | 0 ==> 1 |  |  |
| Semionotiformes | 15 | 1.000 | 0 ==> 1 |  |  |
| (Bremer 1, Bootstrap 44) | 23 | 0.200 | 0 ==> 1 |  |  |
|  | 28 | 0.500 |  |  | 0 ---> 1 |
|  | 29 | 0.167 |  | 1 ---> 0 |  |
|  | 31 | 0.200 |  | 1 ---> 0 |  |
|  | 37 | 0.250 | 2 ==> 0 |  |  |
|  | 73 | 0.167 |  | 1 ---> 0 |  |
|  | 74 | 0.286 |  |  | 0 ---> 1 |
|  | 79 | 0.200 |  |  | 0 ---> 1 |
|  | 82 | 1.000 | 0 ==> 1 |  |  |
| (Callipurbeckiidae, *Semionotus*) | 25 | 0.667 | 0 ==> 1 |  |  |
| (Bremer 1, Bootstrap 23) | 42 | 0.500 | 2 ==> 0 |  |  |
|  | 43 | 0.250 | 0 ==> 1 |  |  |
|  | 73 | 0.167 |  | 0 ---> 1 |  |
|  | 86 | 0.333 | 0 ==> 1 |  |  |

| Brunch | Ch. | CI | Direction of change | | |
| --- | --- | --- | --- | --- | --- |
|  |  |  | Unambiguous | ACCTRAN | DELTRAN |
| Callipurbeckiidae | 22 | 0.222 | 0 ==> 2 |  |  |
| (Bremer 2, Bootstrap 33) | 55 | 0.250 | 0 ==> 1 |  |  |
|  | 66 | 0.500 | 0 ==> 1 |  |  |
|  | 73 | 0.167 |  |  | 0 ---> 1 |
|  | 89 | 0.250 |  | 0 ---> 1 |  |
| (*Paralepidotus* (*Macrosemimimus* | 51 | 0.250 |  | 0 ---> 1 |  |
| (*Callipurbeckia*, *Tlayuamichin*))) | 53 | 0.200 | 0 ==> 1 |  |  |
| (Bremer 2, Bootstrap 23) |  |  |  |  |  |
| (*Macrosemimimus* (*Callipurbeckia*, | 42 | 0.500 | 0 ==> 1 |  |  |
| *Tlayuamichin*)) | 74 | 0.286 | 1 ==> 2 |  |  |
| (Bremer 3, Bootstrap 69) | 79 | 0.200 | 1 ==> 0 |  |  |
|  | 80 | 0.333 | 0 ==> 1 |  |  |
|  | 86 | 0.333 | 1 ==> 2 |  |  |
|  | 89 | 0.250 |  |  | 0 ---> 1 |
| (*Callipurbeckia*, Tlayuamichin) | 51 | 0.250 |  |  | 0 ---> 1 |
| (Bremer 2, Bootstrap 77) | 78 | 0.333 | 0 ==> 1 |  |  |
|  | 83 | 0.400 | 0 ==> 1 |  |  |
| *Macrosemimimus* | 10 | 0.250 | 0 ==> 1 |  |  |
| (Bremer 3, Bootstrap 78) | 22 | 0.222 | 2 ==> 0 |  |  |
|  | 25 | 0.667 | 1 ==> 2 |  |  |
|  | 26 | 1.000 | 0 ==> 1 |  |  |
| *Semionotus* | 7 | 0.250 | 0 ==> 1 |  |  |
| (Bremer 2, Bootstrap 73) | 27 | 0.333 |  | 0 ---> 1 |  |
|  | 29 | 0.167 |  | 0 ---> 1 |  |
|  | 78 | 0.333 | 0 ==> 1 |  |  |
|  | 80 | 0.333 | 0 ==> 1 |  |  |
|  | 83 | 0.400 | 0 ==> 2 |  |  |
| (*Luoxiongichthys*, Macrosemiidae) | 1 | 0.429 | 0 ==> 3 |  |  |
| (Bremer 1, Bootstrap 17) | 6 | 0.500 |  | 0 ---> 1 |  |
|  | 21 | 0.500 |  | 0 ---> 1 |  |
|  | 77 | 0.250 | 0 ==> 1 |  |  |
|  | 88 | 0.400 |  | 0 ---> 2 |  |
| Macrosemiidae | 6 | 0.500 |  |  | 0 ---> 1 |
| (Bremer >4, Bootstrap 100) | 19 | 0.250 | 0 ==> 1 |  |  |
|  | 21 | 0.500 |  |  | 0 ---> 1 |
|  | 22 | 0.222 | 0 ==> 2 |  |  |
|  | 25 | 0.667 | 0 ==> 2 |  |  |
|  | 41 | 0.333 | 0 ==> 1 |  |  |
|  | 52 | 0.400 | 1 ==> 0 |  |  |
|  | 56 | 0.200 | 1 ==> 0 |  |  |
|  | 62 | 1.000 | 0 ==> 1 |  |  |
|  | 69 | 0.200 | 0 ==> 1 |  |  |
|  | 75 | 0.333 | 0 ==> 1 |  |  |
|  | 80 | 0.333 | 0 ==> 1 |  |  |
|  | 88 | 0.400 |  |  | 0 ---> 2 |
| (*Macrosemius*, *Notagogus*) | 17 | 0.600 | 1 ==> 2 |  |  |
| (Bremer 2, Bootstrap 83) | 76 | 0.333 | 0 ==> 1 |  |  |

| Brunch | Ch. | CI | Direction of change | | |
| --- | --- | --- | --- | --- | --- |
|  |  |  | Unambiguous | ACCTRAN | DELTRAN |
| *Sangiorgioichthys* | 36 | 0.222 | 0 ==> 2 |  |  |
| (Bremer 3, Bootstrap 81) | 46 | 1.000 | 0 ==> 1 |  |  |
|  | 53 | 0.200 |  | 0 ---> 1 |  |
|  | 74 | 0.286 |  | 1 ---> 2 |  |
|  | 82 | 1.000 | 1 ==> 2 |  |  |
|  | 83 | 0.400 | 0 ==> 1 |  |  |
|  | 89 | 0.250 |  | 0 ---> 1 |  |
