# Supplementary material for: Phylogenetic Interrelationships of Ginglymodian Fishes (Actinopterygii: Neopterygii)
Source: PLoS One. 2012 Jul 11;7(7):e39370. doi: 10.1371/journal.pone.0039370 (PMC3394768; doi:10.1371/journal.pone.0039370)
Supplement: Text S1 — List of material examined. (DOCX) [file pone.0039370.s002.docx]

# Supporting text (Text S1) to:

# Phylogenetic Interrelationships of Ginglymodian Fishes (Actinopterygii: Neopterygii)

Adriana López-Arbarello

Bayerische Staatssammlung für Paläontologie und Geologie, Richard-Wagner-Strasse 10, D-80333 München, Germany. E-mail: a.Lopez-Arbarello@lrz.uni-muenchen.de

# List of material examined

**Institutional abbreviations.** AMNH, American Museum of Natural History, New York, USA; BGS.GSM, British Geological Survey, London, UK; BSPG, Bayerische Staatssammlung für Paläontologie un Geologie, München, Germany; GMPKU, Geological Museum of Peking University, Beijing, China; GZG.V, Geowissenschaftliches Zentrum der Georg-August-Universität, Göttingen, Germany; IGM, Instituto de Geología, Universidad Nacional Autónoma de México; JM, Jura-Museum Eichstätt, Germany; MB, Museum für Naturkunde, Leibniz-Institut für Evolutions- und Biodiversitätsforschung an der Humboldt-Universität, Berlin, Germany; MHIN-UNSL-GEO, Museo de Historia Natural de la Universidad Nacional de San Luis, San Luis, Argentina; MHNH, Muséum d’histoire naturelle, Le Havre, France; MMP, Museo Municipal de Ciencias Naturales de Mar del Plata, Argentina; MNHN, Muséum National d’Histoire Naturelle, Paris, France; NHMUK, Natural History Museum, London, UK; NMB, National Museum, Bloemfontein, South Africa; NMC, Naturkunde-Museum Coburg, Coburg, Germany; SMF, Senckenberg Forschungsinstitut und Naturmuseum, Frankfurt am Main, Germany; SMNS, Staatliches Museum für Naturkunde, Stuttgart, Germany.

*Araripelepidotes temnurus* (Agassiz, 1841) [1]: BSPG 1965-I-132; MNHN BCE-334, 335, 336, 399, and 340; and detailed high quality photogrphas of AMNH 11813, 11832, 11833, 12716, 11614.

*Atractosteus spatula* (Lacépède, 1803) [2]: based on Grande [3].

*Callipurbeckia minor* (Agassiz, 1833) [4] (*Lepidotes minor*): BGS.GSM 27975 (neotype), MBf. 1617–9, NHMUK PV P.1118, P6343, P.8047, P.29398, P28399, 19006, 21349, 21974, 36080, 41157, 44848, 48255, 48341.

*Dentilepisosteus laevis* Wenz and Brito, 1992 [5]: based on Grande [3].

*Isanichthys palustris* Cavin and Suteethorn, 2006 [6]: based on Cavin and Suteethorn [6].

*Lepidotes elvensis* (de Blainville, 1818) [7]: MNHN JRE-250, 251, 254, 545 (holotype).

*Lepidotes gigas* Agassiz, 1832 [8]: BSPG AS-I-625, 1940-I-8, MBf. 1372, 1495, 1519, MNHN HLZ-32, NHMUK PV P.14539.

*Lepidotes semiserratus* Agassiz, 1836 [4]: NHMUK PV P.3528, P.3528a, P.7410, 35556, 62939.

*Lepisosteus* *osseus* (Linnaeus, 1758) [9]: based on Grande [3].

*Luoxiongichthys hyperdorsalis* Wen et al., in press [10]: BSPG 2010-I-131.

*Macrosemimimus fegerti* Schröder et al., 2012 [11]: JME ETT-854 (holotype), JME ETT-67, 77, 172, 244, 853, 888, 978, 1351; *Macrosemimimus* *lennieri* Sauvage, 1893 [12]: MHNH 7267 (holotype of *Lepidotes lennieri*) and *Lepidotes* *toombsi* Jain and Robinson, 1963 [13]: NHMUK PV P.2518 (holotype), P.34511.

*Macrosemius rostratus* Agassiz, 1844 [4]: BSPG 1986-XV-119 and Bartram [14]; *Macrosemius fourneti* (Thiollière, 1858) [15]: BSPG AS-I-768 (holotype) and Bartram [14].

*Masillosteus kelleri* Micklich and Klappert, 2001 [16]: based on Micklich and Klappert [16] and Grande [3]; *Masillosteus janeae* Grande, 2010 [3]: based on Grande [3].

*Neosemionotus puntanus* Bocchino, 1973 [17]: MMP 1114 (holotype); MHIN-UNSL-GEO-V8, V64, V68, V183–185, V187–191.

*Notagogus denticulatus* Agassiz, 1839 [4]: BSPG AS-I-768 (holotype), 1967-I-303, 1986-XV-120 and Bartram [14]; *Notagogus decoratus* Eastman, 1914 [18]: BSPG 1986-XV-119 and Bartram [14].

*Obaichthys decoratus* Wenz and Brito, 1992 [5]: based on Grande [3]; *Obaichthys* *africanus* Grande, 2010 [3]: BSPG 1993-IX-320 (scales).

*Paralepidotus ornatus* (Agassiz, 1833) [4]: BSPG 2003-XXIX-218, 2003-XXIX-632, 2007-I-62 and Tintori [19].

*Pliodetes nigeriensis* Wenz, 1999 [20]: MNHN GDF-1274, 1275 (holotype), 1276–78, 1285, 1288.

*Propterus microstomus* Agassiz, 1834 [4]: AS-VII-268 (holotype), AS-I-637, 2011-I-139 and Bartram [14]; *Propterus elongatus* Wagner, 1863 [21]: BSPG AS-I-767 (holotype), 1963-I-173, 1964-23-145, 1986-XV-121, 2011-I-138 and Bartram [14].

*Sangiorgioichthys aldae* Tintori and Lombardo, 2007 [22]: based on Tintori and Lombardo [19]; *Sangiorgioichthys sui* López-Arbarello et al., 2011 [23]: GMPKU-P-1568 (holotype), GMPKU-P-1357–9, 1472, 1558, 1567-8, 1609, 1636, 1642, 1656, 1661, 1695, 1707, 1973, 1995.

*Scheenstia decoratus* (Wagner, 1863) [21] (*Lepidotes decoratus*): BSPG AS-VI-3 (holotype); *Scheenstia degenhardti* (Branco, 1885) [24] (*Lepidotes degenhardti*): MBf1499 (holotype); *Scheenstia laevis* (Agassiz, 1837) [4] (*Lepidotes laevis*): MNHN-CRN 61; *Scheenstia mantelli* (Agassiz, 1833) [4] (*Lepidotes mantelli*): NHMUK PV 2397, 2401, 2456 (holotype), 3036–7, 3048–49, 3517–8, 4916, P.6336, P.6344–5, P.6348c, P.6362, P.6933, P.7333, P.11832–3, P.14451; *Scheenstia maximus* (Wagner, 1863) [21]: SMF P.2386 (neotype), P.325; *Scheenstia zapi* López-Arbarello and Sferco, 2011 [25]: JME-Scha 80 (holotype).

*Semiolepis brembanus* Lombardo and Tintori, 2008 [26]; *Semiolepis* sp. NHMUK PV P.10943

*Semionotus bergeri* Agassiz, 1832 [10]: BSPG AS-I-512, 1960-I-25, 1960-XVI-2; GZG.V.010.001 (lectotype), GZG.V.010.002-4, MB f. 1518 (4 specimens); NMC 1264, 15128a–b, 15129; SMF P6108; *Semionotus capensis* Woodward, 1888 [27]: BSPG 1908-I-502, MB f. 12240 (9 specimens), photographs of several specimens in the NMB; *Semionotus elegans* (Newberry, 1888) [28]: Olsen and McCune [29].

*Tlayuamichin itztli* López-Arbarello and Alvarado-Ortega, 2011 [30]: IGM 6716 (holotype), IGM 6717–20.

# References

1. Agassiz L (1841) On the fossil fishes found by Mr. Gardner in the Province of Ceará, in the North of Brazil. Edinburgh New Philosophical Journal 30, 82–84.
2. Lacépède BGE (1803) Histoire naturelle des poissons 5. Paris: Plasson. 803 p.
3. Grande L (2010) An empirical synthetic pattern study of gars (Lepisosteiformes) and closely related species, based mostly on skeletal anatomy : the resurrection of Holostei. Am Soc Ichthyol Herpetol, Spec Publ 6: 1-871.
4. Agassiz L (1833-1844) Recherches sur les Poissons Fossiles. Neuchâtel et Soleure: Petitpierre. 1420 p.
5. Wenz S, Brito PM (1992) Primière découverte de Lepisosteidae (Pisces, Actinopterygii) dans le Crétacé inférieur de la Chapada do Araripe (N-E, du Brésil). Conséquences sur la phylogénie des Ginglymodi. Comptes Rendus de l'Académie des Sciences, Paléontologie 314(II): 1519-1525.
6. Cavin L, Suteethorn V (2006) A new semionotiform (Actinopterygii, Neopterygii) from Upper Jurassic - Lower Cretaceous deposits of North-East Thailand, with comments on the relationships of semionotiforms. Palaeont 49(2): 339-353.
7. Blainville HDd (1818) Sur les ichthyolites ou les poissons fossiles. In: Levrault FG, editor. Nouveau dictionnaire d'histoire naturelle, appliquée aux arts, à l'economie rurale et domestique, à la Medicine, etc. Paris: Deterville. pp. 310-395.
8. Agassiz L (1832) Untersuchungen über die fossilen Fische der Lias- Formation. Neues Jahrbuch für Mineralogie, Geognosie, Geologie und Petrefaktenkunde 3: 139–149.
9. Linnaeus C (1758) Systema naturae er regna tria naturae, secundum classes, ordines, genera, species, cum characteribus, differentiis, synonymis, locis. Tomus 1. Editio decima, reformta. Holmiae: 824 pp.
10. Wen W, Zhang QY, Zhou CY, Huang JY, Chen ZQ, et al. (2012) A new genus of basal actinopterygian fish from the Anisian (Middle Triassic) of Luoping, Yunnan Province, Southwest China. Acta Palaeontologica Polonica. 57(1): 149-160.
11. Schröder KM, López-Arbarello A, Ebert M (2012) *Macrosemimimus* gen. nov. (Actinopterygii, Semionotiformes) from the Late Jurassic of Germany, England and France. Journal of Vertebrate Paleontology32(3): 512–529.
12. Sauvage HE (1893) Description de deux espèces nouvelles de Poissons du terrain kimméridgien du Cap de la Hève. Bulletin de la Société Géologique de la Normandie 14: 3-7.
13. Jain SL, Robinson PL (1963) Some new specimens of the fossil fish Lepidotes from the English Upper Juassic. Proceeding of the Royal Society of London 141: 119-135.
14. Bartram AWH (1977) The Macrosemiidae, a Mesozoic family of holostean fishes. Bull British Mus (Nat Hist), Geol 29(2): 137-234.
15. Thiollière V (1858) Note sur les poissons fossiles du Bugey, et sur l'application de la méthode de Cuvier à leur classement. Bulletin de la Société géologique de France 15: 782-793.
16. Micklich N, Klappert G (2001) *Masillosteus kelleri*, a New Gar (Actinopterygii, Lepisosteidae) from the Middle Eocene of Grube Messel (Hessen, Germany). Kaupia 11: 73-81.
17. Bocchino A (1973) Semionotidae (Pisces, Holostei, Semionotiformes) de la Formación Lagarcito (Jurásico Superior?), San Luis, Argentina. Ameghiniana 10(3): 254-268.
18. Eastman CR (1914) Catalog of the fossil fishes in the Carnegie Museum. 4, Descriptive catalog of fossil fishes from the lithographic stone of Solenhofen, Bavaria. Mem. Carneg. Mus., Pittsburgh 6: 389-423.
19. Tintori A (1996) *Paralepidotus ornatus* (Agassiz 1833-43): A semionotid from the Norian (Late Triassic) of Europe. In: Arratia G, Viohl G, eds. Mesozoic Fishes: Systematics and Paleoecology. München: Verlag Dr. Friedrich Pfeil. pp. 167-179.
20. Wenz S (1999) †*Pliodetes* *nigeriensis*, gen. nov. et. sp. nov., a new semionotid fish from the Lower Cretaceous of Gadoufaoua (Niger Republic): phylogenetic comments. In: Arratia G, Schultze H-P, eds. Mesozoic Fishes 2 – Systematics and Fossil Record. München, Germany: Verlag Dr. Friederich Pfeil. pp. 107-120.
21. Wagner A (1863) Monographie der fossilen Fische aus den lithographischen Schiefern Bayern's. Abhandlungen der Königlich Bayerische Akademie der Wissenschaften W. II. CI. IX. Bd. III. Abth.: 138.
22. Tintori A, Lombardo C (2007) A new early Semionotidae (Semionotiformes, Actinopterygii) from the Upper Ladinian of the Monte San Giorgio area (Southern Switzerland and Northern Italy). Riv Ital Paleont Stratigr 113(3): 369-381.
23. López-Arabello A, Sun ZY, Sferco E, Tintori A, Xu GH, et al. (2011) New species of *Sangiogioichthys* Tintori and Lombardo, 2007 (Neopterygii, Semionotiformes) from the Anisian of Luoping (Yunnan Province, South China). Zootaxa 2749: 25-39.
24. Branco W (1885) Ueber eine neue *Lepidotus* - Art aus dem Wealden. Jahrbuch der königlich preussischen geologischen Landesanstalt 1884: 181–200.
25. López-Arbarello A, Sferco E (2011) New semionotiform (Actinopterygii, Neopterygii) from the Late Jurassic of Southern Germany. J Syst Palaeont 9: 197-215.
26. Lombardo C, Tintori A (2008) A new semionotid fish (Actinopterygii, Osteichthyes) from the Late Triassic of the Northern Italy. In: Arratia G, Schultze H-P, Wilson MVH, eds. Mesozoic Fishes 4. München: Verlag Dr. Friedrich Pfeil. pp. 129-142.
27. Woodward AS (1888) On two new lepidotoid ganoids from the Early Mesozoic deposits of Orange Free State, South Africa. Q J Geol Soc 44: 138-143.
28. Newberry JS (1888) Fossil fishes and fossil plants oft he Triassic rocks of New Jersey and the Connecticut Valley. United States Geological Survey, Monograph 14: 1-152.
29. Olsen PE, McCune AR (1991) Morphology of the *Semionotus elegans* species group from the Early Jurassic part of the Newark Supergroup of eastern North America with comments on the Family Semionotidae (Neopterygii). J Vert Paleont 11(3): 269-292.
30. López-Arbarello A, Alvarado-Ortega J (2011) New semionotiform (Neopterygii) from the Tlayúa Quarry (Early Cretaceous, Albian), Mexico. Zootaxa, 2749: 1–24.
