# Supplementary material for: Phylogenetic Interrelationships of Ginglymodian Fishes (Actinopterygii: Neopterygii)
Source: PLoS One. 2012 Jul 11;7(7):e39370. doi: 10.1371/journal.pone.0039370 (PMC3394768; doi:10.1371/journal.pone.0039370)
Supplement: Text S2 — Data matrix for phylogenetic analysis. (DOCX) [file pone.0039370.s003.docx]

# Supporting text (Text S2) to:

# Phylogenetic Interrelationships of Ginglymodian Fishes (Actinopterygii: Neopterygii)

Adriana López-Arbarello

Bayerische Staatssammlung für Paläontologie und Geologie, Richard-Wagner-Strasse 10, D-80333 München, Germany. E-mail: a.Lopez-Arbarello@lrz.uni-muenchen.de

## Data matrix

*Perleidus* 010000?00??0???00?0000000000000000?000000000000??000001000000010??00?000000000?00000000000

*Watsonulus* 010?0?0000000000010002000000100000?100001?????100001000100000010??011100000000?00000000000

*Amia* 3101001000000000010001000000?0??00?120001?????1000010000000100110011110000111?0????0??0200

*Leptolepis* ??00000001?000?030000211000?000000?220000010000??002000100?10020??0100000011000???00??0000

*Pholidophorus* 2100000001?000?0300002010000000000?220000010000??002000100010020??01?0000011000???00000000

*Siemensichthys* 2??????00?00???030000200000??0??00?22?001?????0??001000100?10020??01?0000000000??000000000

*Dapedium* 000000000000??00100201000000000000??20000200000??001?01?01021001?001010002001000000000???0

*Lepisosteus* 10111111001111011001001000001111110210110300001112?0?100100210110000?210100000101000111211

*Atractosteus* 10(01)1111100111101100200100000111111021(01)110300001112?0?100100210110000?210100000001000111211

*Masillosteus* 1?????1?0??010?1?00100000000111101022(01)1103000011?2?0?110001210110000?210100000001000121201

*Obaichthys* 1011111110?110?1002100000000110?0102110103000011110?00000012001110111210000000????0101?200

*Dentilepisosteus* 10111111101110?1102100100000110101?22101030000111101000100120011000112100?0000001001110201

*Pliodetes* 2?????0?10?????01?2101000000111101012001030000111100100000?200111000?2001?0000????01010200

*Araripelepidotes* 21111000???????0??110100000???1?01022001040000?0?1001?0000?2001110111200100000000001020200

*Isanichthys* 1?????0?0??????0??010000000?1111011021?0020000???00100?10001000110110200?????0????00??0100

*Lepidotes maximus* 0?????0?0100???0??020000000?111101002000020100100????02101010001001102?11?0000000000?20?1?

*Lepidotes laevis* 0?????0?0??????0??020010000?111101002000020100?0?11?102101?1000100110??11?00??????00??0111

*Lepidotes mantelli* 0111100?01?0???0??020010000?11110100200002010010?111102101?10001101102011100000000000201?1

*Scheenstia zappi* 0?????0?0?00???0100200000000111001001000020100100????02100010001101102?112?000000000??0111

*Lepidotes semiserratus* 0?11?00001?000?01000000000001?110110100002000010?001001100?100011011020??200??????001201?1

*Lepidotes gigas* 0???1?0?0?0000?010100000000010110110100002000010000100010001000110110200120?000000001201?1

*Neosemionotus* 0?????0?00?????0?01200000001101001001000040000100001000100010001101112?01??001100010??00?0

*Lepidotes minor* 0?11100?0??000?0?00002101001000001000000011000100011101100010001111102001200010101100201?0

*Tlayuamichin* 0?????1?00?0??10?0010210100100000100000004001010001110110001000111110200120001010110?20010

*Macrosemimimus fegerti* 0????00?01?000?0101?10102101000001000000011000100011101100010001111102?01200000101000?0?10

*Macrosemimimus lennieri* 011110000100001010000010210?001001000000011000100001102101010001111102?0120000????00?200?0

*Semionotus bergeri* 0?????1?0??0???0?0000010101?100001010000001000100???000?00010001101102?0?10001?10?20010000

*Semionotus elegans* 0?111?10000000?010100010100110000100000000100010000100010001000110110200010001?10120??0000

*Semionotus capensis* 0?????1?0?0000?010?10010101?0000010?0000001000100?????0100010001101102?01?0001110120010000

*Paralepidotus* 3?????0?00?0???01?0002101001000001010000001000?0????1?1?000100011111020??10010100100010??0

*Semiolepis* 0???????0??000?01?020210100?00000102000000100010000100110001000111111200110000100110010???

*Macrosemius* spp. 3?11110000?00010201?1210200100?0010000001?????1000000010000101011011120001111?110??00002?0

*Notagogus* spp. 3???????0??????0201?1210200?000001000?001?????1000000000000101011011120001(01)110010?00000200

*Propterus* spp. 3????10?00????10101?121020010000010000001?????10000000000001010111111200011010110100000200

*Sangiorgioichthys sui* 0?????0?0?0000?01001(01)01000010000010(12)0000021001100001(01)00100010001101102?0020000100210000010

*Sangiorgioichthys aldae* 0?????0?0???00????00001000010000010200000200111000011?0100010001101102?0??0?00???2100000?0

*Luoxiongichthys* 3???????0??000?01?0??01000111000010100?1020000?0?0110?0100010001??0102????0010?0010000????
